# Effect of acupuncture on menopausal depressive disorder and serum hormone levels: a systematic review and meta-analysis

**DOI:** 10.3389/fpsyt.2025.1591389

**Published:** 2025-07-14

**Authors:** Shunxia He, Zhijie Wang, Shiqiu Dong, Yuzi Diao, Hongwei Qiao, Xiaoguang Lin, Xiao Gao

**Affiliations:** ^1^ Heilongjiang University of Chinese Medicine, Harbin, China; ^2^ Shanxi Province Hospital of Traditional Chinese Medicine, Taiyuan, China; ^3^ Heilongjiang Nursing College, Harbin, China; ^4^ Graduate School, Gachon University, Seongnam-si, Gyeonggi-do, Republic of Korea; ^5^ Heilongjiang Vocational College of Winter Sports, Harbin, China; ^6^ The Fourth Affiliated Hospital of Heilongjiang University of Chinese Medicine, Harbin, China

**Keywords:** acupuncture, menopausal depressive disorder, menopausal syndromes, meta-analysis, systematic review

## Abstract

**Background:**

Menopause, marked by ovarian decline and hormonal shifts, increases vulnerability to depressive disorders, with menopausal depressive disorder (MDD) affecting 33–36% of women via psychosocial-biological interactions. Acupuncture shows promise in improving MDD through neuroendocrine regulation but lacks robust evidence, with unclear links to reproductive hormone modulation; this study evaluates its efficacy and safety.

**Methods:**

A comprehensive database search was conducted using PubMed, Embase, the Cochrane Library, Web of Science, EBSCO, Scopus, Cnki, Wan Fang and VIP Database to identify randomized controlled trials (RCTs) investigating the impact of acupuncture on menopausal depressive disorder. RCTs published until April 21, 2025, that met our predetermined inclusion and exclusion criteria were included. Data extraction, literature review, and assessment of the methodological quality of the trials were performed. The meta-analysis was conducted using Review Manager (RevMan) 5.3 software.

**Results:**

Our findings demonstrate that acupuncture significantly outperforms control interventions in improving clinical effectiveness rates (OR=2.70, 95%CI[1.63,4.48], P=0.0001) and reducing depressive symptoms, as evidenced by HAMD-17 (SMD=-0.28, P<0.0001) and HAMD-24 scores (post-sensitivity SMD=-0.39, P=0.03). Notably, acupuncture also enhanced quality of life (MENQOL: SMD=-0.25, P=0.003), though its effects on sex hormones (FSH, LH, E2) remained nonsignificant (P>0.05). Safety profiles were comparable between groups (OR=0.16, P=0.05), yet sensitivity analysis revealed reduced adverse events in the acupuncture group after excluding outlier studies (OR=0.49, P=0.03). In conclusion, the intervention of acupuncture is beneficial for MDD.

**Conclusion:**

This systematic review demonstrates that acupuncture serves as an effective and safe non-pharmacological intervention for alleviating menopausal depressive symptoms and improving quality of life. While acupuncture did not significantly modulate sex hormone levels, its therapeutic benefits are likely mediated through non-hormonal mechanisms, such as neurotransmitter regulation and neuroendocrine network modulation.

**Systematic review registration:**

https://www.crd.york.ac.uk/prospero/, identifier CRD420251037010.

## 1 Introduction

Menopause, recognized as a biological marker of the termination of female reproductive function (1), is clinically defined as the permanent cessation of menses (typically confirmed after 12 consecutive months of amenorrhea) (2, 3), characterized by endocrine alterations including diminished ovarian function, reduced estrogen levels, and increased gonadotropin (FSH/LH) concentrations (4, 5). As per the STRAW+10 staging system (6), the menopausal transition encompasses both the perimenopausal phase (stages -2 to -1) and early postmenopausal stage (+1a), wherein the final menstrual period (FMP) serves as the primary diagnostic marker. This transitional process typically commences after age 40 and persists for 4–5 years, while related symptoms may endure for several postmenopausal years (1). The average age at natural menopause ranges from 50–51 years in high-income nations to 40–58 years globally (3).

Menopausal depressive disorder(MDD) typically manifests during the transitional period surrounding menopause and is characterized by a constellation of psychological symptoms including low mood, anxiety, and heightened stress, frequently coexisting with physiological alterations linked to endocrine dysfunction, particularly hypogonadism and age-related hormonal changes (5, 7). Epidemiological evidence demonstrates a substantially greater burden of depression among menopausal women relative to other age groups (8). A meta-analysis revealed an aggregate prevalence of depression of 35.6% among menopausal women, comprising 33.9% during perimenopause and 34.9% in postmenopause (9). Longitudinal data indicate a progressive increase in depressive symptom prevalence across menopausal stages, rising from 14.5% at perimenopause to 19.6% at postmenopause (10), underscoring the elevated vulnerability during the menopausal transition, particularly the perimenopausal phase (10).

Worldwide epidemiological data indicate that approximately 33% of menopausal women experience depressive symptoms, with comparable prevalence rates observed during both perimenopausal and postmenopausal stages (9), demonstrating the sustained vulnerability to depression across the entire menopausal transition. The pathogenesis of menopausal depression represents a multifactorial interplay between psychosocial stressors and biological alterations. Predisposing factors including personal history of depression and neurotic personality traits, combined with environmental challenges such as socioeconomic disadvantage and inadequate social support, substantially elevate the risk for developing depressive disorders (11). Concurrently, biological mechanisms such as estrogen level variability, hypothalamic-pituitary-adrenal (HPA) axis dysregulation, vasomotor symptoms, and neuroinflammatory processes collectively contribute to mood disturbance exacerbation (12).

Recent studies have shown that acupuncture presents a multidimensional regulatory role in improving menopausal depression (13). The mechanism involves the synergistic regulation of neuroendocrine networks and cognitive functions: on the one hand, acupuncture activates the PKA/CREB signaling pathway (14) by regulating the metabolism of neurotransmitters such as 5-hydroxytryptamine (5-HT) (15), enhances synaptic plasticity in the hippocampus and inhibits neuroinflammatory responses (16); on the other hand, acupuncture significantly alleviates estrogen fluctuations induced by hypothalamic-pituitary-gonadal axis (HPO axis) dysfunction caused by estrogen fluctuation and improve the negative effects of hormonal imbalance on the central nervous system (17). In addition, acupuncture can reconfigure patients’ embodied cognitive patterns through enhanced somatosensory inputs, breaking the vicious cycle of “hormone-symptom-emotion” and improving sleep quality and cognitive function (17). Clinical evidence shows that acupuncture alone or in combination with antidepressants can significantly reduce depression scale scores, and has long-term efficacy in improving anxiety symptoms and vasodilatory symptoms (e.g., hot flashes), and maintains a stable effect 6 months after treatment (18). Notably, the modulating effect of acupuncture on serum reproductive hormone (e.g., FSH, LH, E_2_) levels may be closely related to its antidepressant efficacy. However, the specific mechanisms by which acupuncture intervenes in hormone dynamics have not been fully elucidated in existing studies.

The menopausal period is considered a critical period in the development of depression in the female life cycle. The prevalence is particularly high during this stage. Given that acupuncture treatment for MDD has fewer side effects, it is gradually gaining recognition in clinical application. However, for the time being, the evidence supporting acupuncture for MDD is insufficient, and the depth of relevant studies is lacking. Therefore, the aim of this study is to investigate the efficacy and safety of acupuncture in the treatment of MDD.

## 2 Materials and methods

### 2.1 Database search protocol

Research studies from the beginning until April 21, 2025, were sought in electronic databases. Acupuncture, a key aspect of traditional Chinese medicine, has a considerable amount of related research that was first published in Chinese. Chinese databases hold a vast array of acupuncture studies, notable for both their quantity and the variety of study designs and populations involved. As a result, these databases were incorporated into the data collection for this research. Six international databases and three Chinese databases were searched: PubMed, Embase, Web of Science, Cochrane Central Register of Controlled Trials, EBSCO, Scopus, China National Knowledge Infrastructure, Wanfang Database and VIP Database for Chinese Technical Periodicals.

The combinations of MeSH terms and keywords include “acupuncture”, “electroacupuncture”, “auricular acupuncture”, “menopause” and “perimenopause”, “menopause”, “depression”, “menopause” and “perimenopause”, and “menopause”. “, “depression” and “depressive disorder”. Search:(acupuncture) OR (electroacupuncture) OR (auricular acupuncture) AND (menopause) OR (menopausal) OR (perimenopausal) AND (depression) OR (depression)). We also screened the reference list of prior SRs associated with perimenopausal depression and acupuncture for eligible trials. There were no language restrictions. For non-Chinese and English literature that met the inclusion criteria, one of the authors, who is proficient in additional languages, handled the translation and data extraction. This approach aligns with common practices in the field, ensuring the completeness and accuracy of data extraction. The translated data were then double-checked by two independent researchers to minimize errors. (See the **Supplementary Document 9** for specific searches of relevant databases).The study protocol (registration No. CRD420251037010) was registered in PROSPERO.

### 2.2 Selection criterion

**Figure 1:**
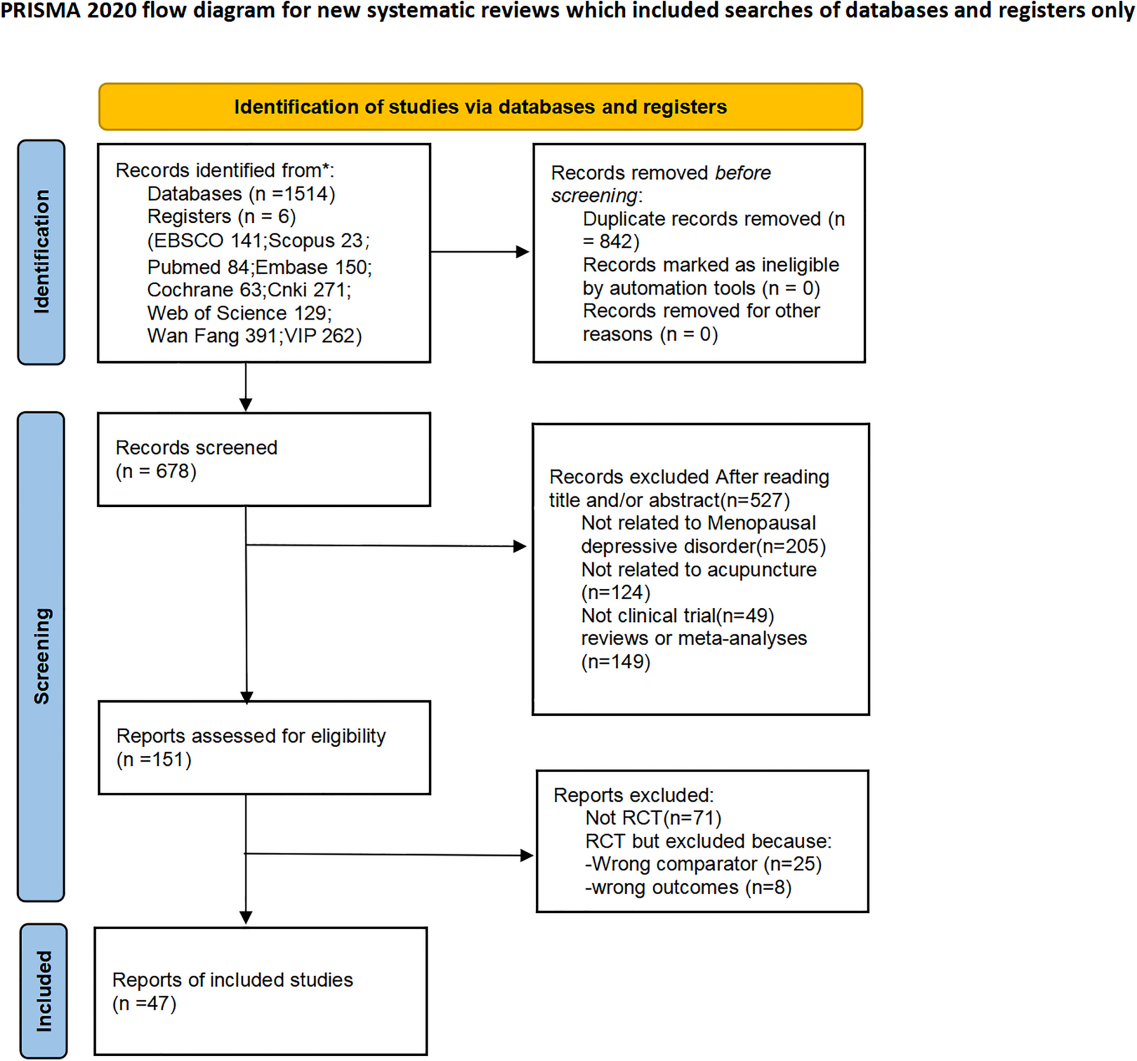
Flow diagram of the included studies.

Inclusion criteria:

This research adhered to the Preferred Reporting Items for Systematic Reviews and Meta-Analyses (PRISMA) guidelines for reviewing literature, as shown in the flowchart in **Figure 1**. The research questions were developed using the PICOS framework.

1. Study design: This review included only randomized controlled trials (RCTs) that evaluated interventions for perimenopausal depression or menopausal depressive disorder in comparison to at least one control intervention.
2. Population: Individuals who have been diagnosed with perimenopausal depression or menopausal depressive disorder.
3. Interventions: Research examining the impact of acupuncture methods (such as electroacupuncture, manual acupuncture, and auricular acupuncture) on depression during the perimenopausal and menopausal stages, along with serum hormone levels. Control interventions consisted of medication, placebo, or sham acupuncture.
4. Outcome indicators: included trials must report at least one of the following forms of outcome: Hamilton Depression Scale (HAMD) (end score or from baseline to end of study), amount of clinical treatment effective, the Self-Rating Depression Scale (SDS), or serum estradiol (E2), luteinizing hormone (LH) or follicle-stimulating hormone (FSH) levels.

Exclusion criteria:

1. non-randomized studies: non-randomized studies, such as observational studies or case reports, were excluded because of confounding factors and bias.
2. non-human studies: studies conducted in animals or *in vitro* experiments were excluded.
3. literature reviews: literature reviews were not included in the analysis because they did not provide original study data.
4. studies that did not comply with the PICOS protocol: studies that did not comply with the PICOS framework established for this review were excluded.
5. studies with insufficient data: studies that did not provide adequate data or assess cognitive parameters and outcome details were excluded.

### 2.3 Study selection and data extraction

Two authors (SXH and ZJW) independently reviewed all articles. Any disagreements were resolved through group discussions and by a third review author (SQD). The flowchart of the study selection process is shown in **Figure 1**. The two review authors also independently extracted data, recording the following information on a data sheet: study details (authors, year of publication, sample size, follow-up), patient characteristics (age range, diagnostic criteria), details of the acupuncture intervention and the control group, endpoints (primary and secondary), discontinuation, and adverse events (AEs). One reviewer reached out to authors via email to obtain any inadequate or missing data. The primary outcomes assessed included the Hamilton Depression Scale (HAMD-17), clinical effectiveness, and the Self-Rating Depression Scale (SDS), while the secondary outcome focused on serum hormone levels.

### 2.4 Quality assessment

Two independent researchers, YZD and HWQ, carried out a two-phase quality evaluation process. Initially, they conducted a preliminary screening using the Jadad Scale (ranging from 0 to 5 points) to evaluate the quality of studies based on three key areas: randomization procedures (0–2 points), implementation of blinding (0–2 points), and reporting of attrition/follow-up (0–1 point). Studies that received a score of 3 points or higher were deemed moderate to high quality and were included for further analysis (19). Following this, the eligible studies underwent a thorough risk-of-bias assessment utilizing the Cochrane Collaboration’s tool (20). The evaluation focused on three categories: low risk of bias, unclear risk of bias, and high risk of bias. The characteristics assessed included random sequence generation (selection bias), allocation concealment (selection bias), blinding of participants and personnel (performance bias), incomplete outcome data (attrition bias), selective reporting (reporting bias), and other biases. These elements were graphed and analyzed using Review Manager 5.3 (Cochrane Collaboration). Any disagreements were resolved through discussions among the authors or with the assistance of a third member (XGL and XG) when necessary.

### 2.5 Statistical analyses

We performed a meta-analysis using Review Manager (RevMan) version 5.3, created by the Cochrane Collaboration. We grouped studies with similar interventions and outcome measures. For continuous data, we computed the Standardized Mean Difference (SMD) along with the 95% confidence interval (CI). We evaluated heterogeneity among the studies using Cochrane’s Q statistic and the I² statistic. If the I² value was below 50%, we used a fixed-effects model; if it was higher, we opted for a random-effects model. A sensitivity analysis was conducted to pinpoint sources of heterogeneity by re-evaluating the pooled effects through a one-by-one elimination approach.

### 2.6 Sensitivity analysis

When heterogeneity was significant, low-quality studies were excluded in turns and meta-analyses were repeated. Results were compared and causes of heterogeneity were discussed.

### 2.7 Assessment of reporting bias

With enough preliminary research, funnel plots were generated to assess potential publication bias qualitatively when at least 10 articles were included. Funnel plots were visually inspected for asymmetry, and Egger test was used to perform sensitivity analysis and statistically assess publication bias. All statistical analyses were performed using RevMan5.3.

## 3 Results

A total of 1,514 studies were initially searched, and six additional articles were identified through manual searching. After evaluation by two independent reviewers (SXH and ZJW), 519 studies were excluded due to duplication. The titles and abstracts of the remaining 678 articles were reviewed, resulting in 151 articles selected for full-text review. Ultimately, 47 randomized controlled trials (RCTs) reported in these 47 articles were included for analysis and evaluated using the Jadad scale, which yielded quality ratings of high (4 studies), moderate (9 studies), and low (24 studies). The quality assessment of the included studies is presented in **Table 1**). After applying predefined inclusion criteria that restricted eligibility to moderate- and high-quality evidence, 13 RCTs were ultimately incorporated into the final analysis (13, 17, 21–31)., and the characteristics of the study selection process are shown in **Figure 1**. The 13 trials analyzed were all randomized controlled trials conducted in China and published in English and Chinese, respectively, between 2007 and 2023. Two of the trials (17, 24) had participants from six different hospitals in China and were multicenter randomized controlled trials, and the remaining trials (13, 21–23, 25–31) were all single-center randomized controlled trials. 1293 patients with MDD ranging in age from 42 to 60 years were included, with sample sizes varying between 58 and 222. **Table 2** shows the detailed characteristics reported in the medical records. Eight studies (13, 17, 21, 22, 24, 26–28, 30) assessed depression-relieving effects by HAMD-17 scale, three studies (25, 29, 31) assessed depression-relieving effects by HAMD-24 scale, seven studies (22, 23, 25, 27–29, 31) assessed treatment effects of MDD by clinical validity, six studies (13, 17, 24, 29–31) assessed follicle-stimulating hormone (FSH) and estradiol (E2) levels, five studies (13, 17, 24, 30, 31) assessed luteinizing hormone (LH),three studies (23, 25, 26) assessed anxiety-relieving effects by SDS scale.

**Table 1:** Quality assessment of included studies.

Authors	Year	Randomization	Blinding	Withdrawals and dropouts	Jadad score
Zhou SH, 2004	2004	1	0	1	2
Guo YM, 2005	2005	1	0	1	2
Ding L, 2007	2007	1	0	0	1
Qian J,2007 (21)	2007	2	0	1	3
Deng AJ, 2008 (22)	2008	2	0	1	3
Qiang BQ, 2008	2008	2	0	0	2
Ma J, 2009	2009	1	0	0	1
Wang XY, 2010	2010	2	0	1	3
Zhang YL, 2010 (10)	2010	2	0	1	3
Zheng SH, 2010	2010	1	0	0	1
Chen Z, 2010 (23)	2010	1	0	0	1
Chi H, 2011	2011	1	0	0	1
Xing K, 2011	2011	2	0	0	2
Ma YB, 2011	2013	1	0	1	2
Zhang YQ, 2013	2013	1	0	0	1
Dong Y, 2015	2015	1	0	1	2
Li HB, 2015	2015	1	0	1	2
Li ZF, 2015 (24)	2015	2	2	1	5
Ning Y, 2015	2015	2	0	0	2
Sun YJ, 2015	2015	1	0	0	1
Wang C, 2015 (25)	2015	2	0	1	3
Zhang J, 2015	2015	0	0	0	0
Niu XS, 2017	2017	2	0	0	2
Li S, 2018 (26)	2018	2	2	1	5
Shi J, 2018	2018	1	0	0	1
Liu HF, 2019	2019	2	0	0	2
Li P, 2020	2020	2	0	0	2
Dai W, 2022	2022	2	0	0	2
Men SJ, 2022	2022	2	0	0	2
Zhou JH, 2022 (17)	2022	2	0	1	3
Zhao FY, 2023	2023	2	2	1	5
Liang ZQ, 2024 (7)	2024	2	0	0	2
Shi XL, 2010	2010	1	0	0	1
Chen GZ, 2010 (23)	2010	2	0	1	3
Shi XL, 2011	2011	1	0	0	1
Li N, 2012	2012	1	0	1	2
Xie YQ, 2013	2013	1	0	0	1
Huang HL, 2016	2016	1	0	0	1
Huang HL, 2017	2017	2	0	0	2
Sui L, 2019	2019	1	0	1	2
Tang NL, 2019 (10)	2019	1	0	0	1
Che JX, 2020	2020	1	0	0	1
Gu T, 2020 (27)	2020	2	0	1	3
Pan L, 2021	2021	1	0	0	1
Wu Y, 2022	2022	2	0	0	2
Liu XY, 2022	2022	2	0	1	3
Wang J, 2023 (28)	2023	2	1	1	4

**Table 2:** Characteristics of included studies.

Study	Time	Diagnostic criteria	Experimental group				Control group				Event	Period of time	Follow-up	AEs
			Sample size	Age	Interventions	Time, frequency	Sample size	Age	Interventions	Time, frequency				
Qian 2007 (21)	2007	CCDM-e	33	45-60	MA Feishu (BL13), Xinshu (BL15), Ganshu (BL18), Pishu (BL20), Shenshu (BL23), and Geshu (BL17).	25min/d, 5d/w	30	46-60	Fluoxetine	20mg/d	HAMD-17 TESS	6w	NA	11
Deng 2008 (22)	2008	ICD-10	30	50.03 ± 4.43	MA(Abdominal Acupuncture) Zhongwan (RN12), Xiawan (RN10), Qihai (RN6), Guanyuan (RN4), Zhongji (RN3), Xiafengshidian (EX-LE5, bilateral), Shangqu (KI17, left), and Qipang (EX-CA4, left)	20–30 min Daily×3d → q3d	30	48.70 ± 4.93	Flupentixol Melitracen Tablets	20mg/d	HAMD-17 KI 5-HT	4w	1m	NA
Wang 2010 (29)	2010	CCMD-3	30	49.60 ± 4.3	MA (Abdominal Acupuncture) Zhongwan (RN12), Xiawan (RN10), Qihai (RN6), Guanyuan (RN4), Shangqu (KI17, left), Zhongji (RN3)	20–30 min Daily×3d → q3d	30	48.3 ± 4.7	Flupentixol Melitracen Tablets	1 tablet/day	HAMD-17	4w	4w	NA
Zhang 2010 (30)	2010	CCMD-3	52	48.48 ± 5.39	EA 6V,8-9mA Group A: Baihui (DU20), Neiguan (PC6), Taichong (LR3), Taixi (KI3), and Sanyinjiao (SP6); Group B: Feishu (BL13), Xinshu (BL15), Ganshu (BL18), Pishu (BL20), and Shenshu (BL23).	Each group was applied on alternate days 30min/d, 5d/w	52	48.48 ± 5.39	Nilestriol Fluoxetine Hydrochloride Capsules	Nilestriol (2mg/tablet): 1 tablet orally every 2 weeks;Fluoxetine Hydrochloride Capsules (20mg/capsule): 1 capsule orally once daily in the morning	HAMD-24 KMI FSH,E2,LH	12w	NA	NA
Chen 2010 (23)	2010	DSM-IV	30	48.1 ± 4.8	MA+CHM MA: Shenshu(BL23),Ganshu(BL18),Xinshu (BL15),Zusanli(ST36),Sanyinjiao(SP6), Shenting(DU24),Benshen(GB13),Sishencong(EX-HN1),Neiguan(PC6) CHM: Zishen Shugan Ningxin Formula	MA:30min/d CHM:once daily	30	48.1 ± 4.8	Zishen Shugan Ningxin Formula	once daily	HAMD-24 FSH,E2 5-HIAA NE DA	8w	NA	NA
Li 2015 (24)	2015	STRAW-10 ICD-10	30	49.80 + 3.39	EA dense-spare waves,10/50Hz,0.5-1.0mA Guanyuan (RN4),Zigong (EX-CA1)Tianshu(ST25),Sanyinjiao (SP6),Hegu(LI4),Taichong(LR3),Baihui(DU20),and Yintang (EX-HN3)	30min/d,3d/w	30	49.90 + 2.98	escitalopram	10mg/d	HAM-D17 MENQ0L E2,FSH,LH AST/ALT/TBIL BUN/Cr	12w	12w	27
Wang 2015 (25)	2015	CCMD-3	35	48.72 ± 4.21	MA Lieque (LU7, left) and Zhaohai (KI6, right); Neiguan (PC6, right) and Gongsun (SP4, left)	30min/d 3d/w	35	48.64 ± 4.82	Wuling Capsules	a dosage of 3 capsules per administration Three times daily	SDS MENQOL	8w	12w	NA
Li 2018 (26)	2018	DSM-5 ICD-10	116	49.83 ± 3.1	EA dilatational wave,50 HZ,0.5-1mA. Guanyuan(RN4), Zigong(EX-CA1, bilateral), Tianshu(ST25, bilateral), Sanyingjiao (SP6, bilateral), Hegu (LI4,bilateral), Taichong (LR3, bilateral), Baihui (DU20), and Yintang (EX-HN3).	30min/d,3d/w	106	49.93 ± 3.1	escitalopram	10mg/d	HAMD-17 MENQOL FSH,LH,E2	12w	12w	32
Gu 2020 (27)	2020	DSM-5 ICD-10	30	49 ± 3	MA+CHM Acupoints: Shuigou (GV26), Shaoshang (LU11), Yinbai (SP1), Daling (PC7), Shenmai (BL62), Jiache (ST6), Chengjiang (CV24), Laogong (PC8), Shangxing (GV23), Quchi (LI11) CHM: kaixin powder	MA:3d/w CHM:1 dose/d, divided bid	28	50 ± 3	kaixin powder	1 dose/day, divided bid (morning & evening)	HAMD-24 SDS KI	12w	1m	NA
Zhou 2022 (17)	2022	STRAW-10 DSM-5	108	45-55	EA dense-spare waves,50Hz,0.5-10mA Baihui(DU20), Yinngtang(EX-NH3), Guanyuan(RN4), Zigong(EX-CA1,bilateral), Tianshu(ST25,bilateral), Hegu(L14,bilateral), Taichong(LR3,bilateral), and Sanyinjiao(SP6,bilateral).	30min, 3d/w	104	45-55	escitalopram	10mg/d or 5mg/d	HAMD-17 MENQOL FSH,LH,E2	12w	13-24w	NA
Liu 2022 (31)	2022	CCMD-3	32	49.88 ± 3.56	MA+CHM MA: Baihui (DU20), Huangshu (KI16, bilateral), Danzhong (RN17), Sanyinjiao (SP6, bilateral) CHM: Buyang Huanwu Decoction	MA:20 min/d,6 consecutive daysecutiv break CHM:1 dose/day, divided bid (morning & evening), taken warm,Administered for 1 month → 2-day break	32	48.97 ± 2.68	Buyang Huanwu Decoction	1 dose/day, divided bid (morning & evening), taken warm Cycle: Administered for 1 month → 2-day break	HAMD-17 KI hs-CRP TCM syndrome score clinical efficacy	2m	NA	NA
Zhao 2023	2023	STRAW ICD-10 ICSD-3	35	48.94 ± 2.25	MA Yintang (EX-HN3), Baihui (GV20), Guanyuan(CV4), Yinjiao (CV7) and bilateral Neiguan (PC6), Taixi (KI3),Taichong (LR3), Sanyinjiao (SP6), and Zigong (EX-CA1).	30min,three sessions per week for the first 3 weeks, two sessions per week for the next 3 weeks, and one session per week for the final 2 weeks	35	48.80 ± 2.07	SA Zhouliao(LI12),Shouwuli(LI13),Tiaokou(ST38), Yangfu(GB38), Xuanzhong(GB39), Sanyangluo(TE8), and Sidu(TE9).	30min,three sessions per week forthe first 3 weeks, two sessions per week for the next 3 weeks, and one session per week for the final 2 weeks	HAM-D17 PSQI FSH,LH,E2 KI	8w	16w	NA
Wang 2023 (28)	2023	DSM-IV	31	49.71 ± 4.29	MA(Abdominal Acupuncture)+CHM MA: Zhongwan(RN12), Xiawan(RN10),Qihai(RN6), Guanyuan(RN4), Shangqu(KL17), Shangfengshi Point(extra-point) and Qipang (extra-point) CHM: BushenTiaogan (BSTG) formula	MA:20 min, Dailyant)q3d CHM: two packs daily	32	51.16 ± 3.99	CHM+SA	SA:20 min, Dailyant)q3d CHM:two packs daily	GCS SDS SAD	8W	12W	5

HAMD, the Hamilton Depression Rating Scale; MENQOL, the menopause-specific quality of life scale; SDS, self-rating depression scale; KI, Kupperman Index; PSQI, Pittsburgh Sleep Quality Index; FSH, Follicle-stimulating hormone; LH, luteinizing hormone;E2,estrogen;STRAW, Stages of Reproductive Aging Workshop;ICD-10,The International Classification of Diseases-Ten Edition;ICSD-3,International Classification of Sleep Disorders Third Edition; CHM, Chinese herbal medicine; MA, Manual Acupuncture; EA, Electroacupuncture; SA, Sham Acupuncture; NA, Not Applicable.

### 3.1 Risk of bias

**Figure 2:**
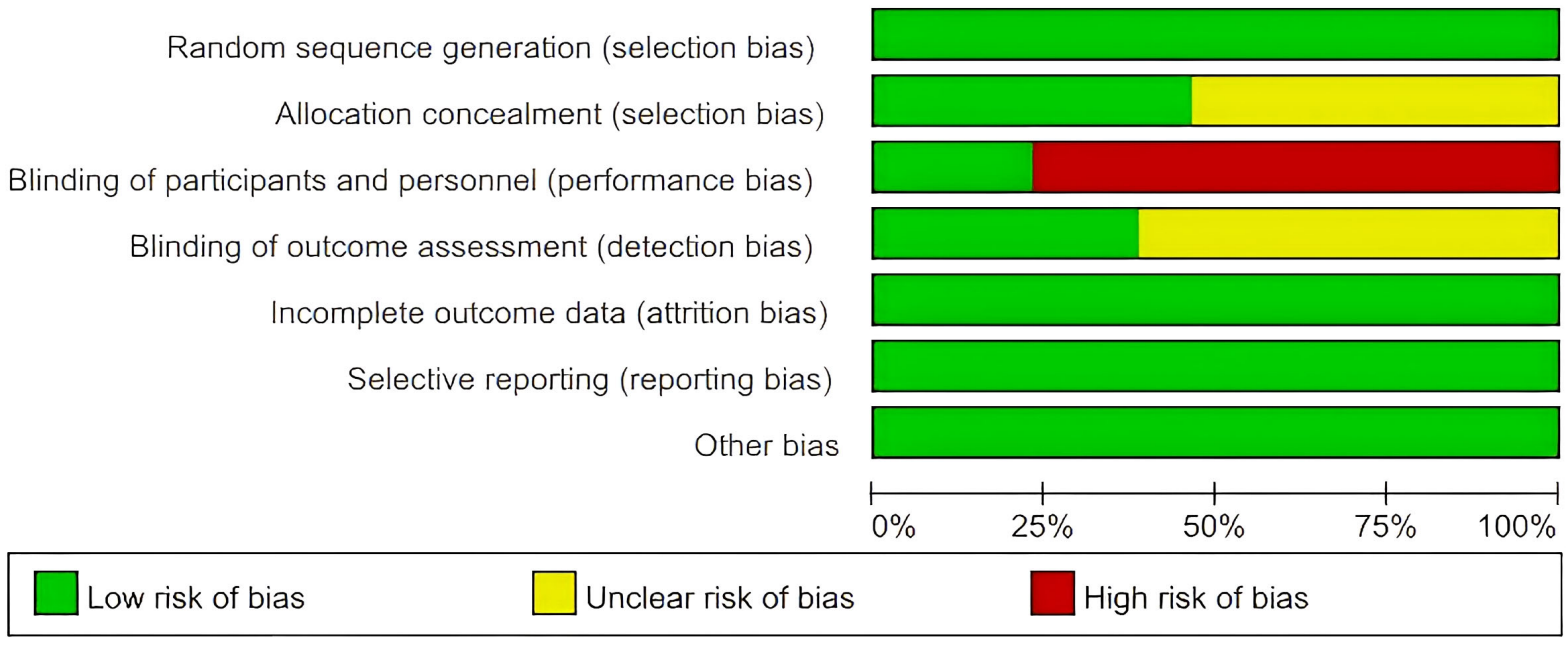
A summary of the overall RoB assessment.

**Figure 3:**
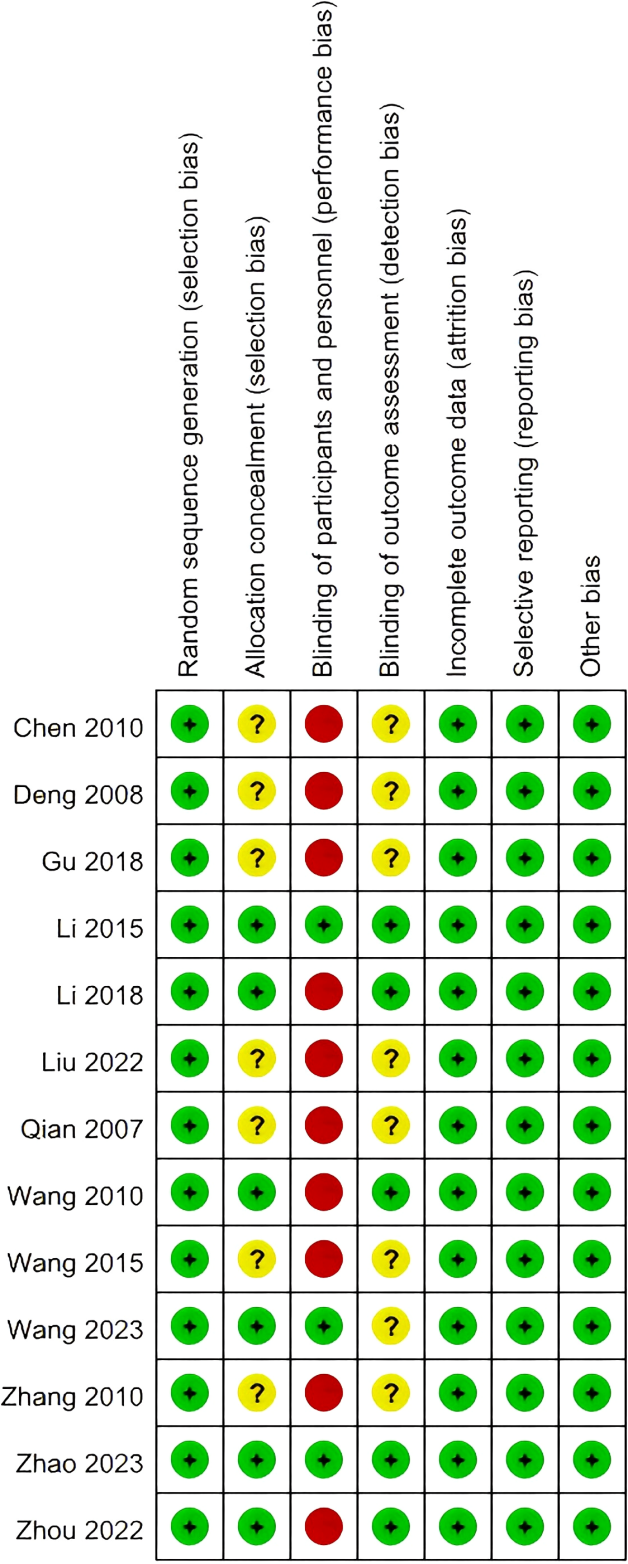
Details of the RoB assessment.

All studies (13, 17, 21–31) employed adequate methods for random sequence generation using a randomization table, and thus were rated as low risk for selection bias regarding random sequence generation. Six studies (13, 17, 24, 26, 27, 30) reported the use of opaque envelopes for allocation concealment, while seven studies (21–23, 25, 28, 29, 31) were rated as having an unclear risk of selection bias concerning allocation concealment, as they did not provide detailed information about the random number generation process. Three studies (13, 26, 30) utilized sham acupuncture in the control group, resulting in a low risk of performance bias. In contrast, the other studies (17, 21–25, 27–29, 31) were rated as high risk, as they were rated as being at low risk. Others studies (17, 21–25, 27–29, 31)were high risk as all were open-label studies without sham acupuncture, making participant blinding impossible by design. Five studies (13, 17, 24, 27, 30) reported blinding of outcome assessment, while the others did not; therefore, only these five studies were rated as low risk for detection bias, while the remaining studies were deemed to have an unclear risk. All studies reported all expected outcomes and data, leading to an evaluation of low risk for attrition bias. Additionally, all studies provided detailed information regarding reasons for dropouts, resulting in a judgment of low risk for reporting bias. Other sources of bias were considered low risk for all studies, as baseline information, findings, ethical approval, and additional details were fully reported. A summary of the overall risk of bias (RoB) assessment is presented in **Figure 2**, while the details of the RoB assessment are shown in **Figure 3**.

### 3.2 Primary outcome

#### 3.2.1 Clinical effectiveness rate

Among the included studies, seven investigations (22, 23, 25, 27–29, 31) evaluated the treatment effects of Major Depressive Disorder (MDD) based on clinical validity, encompassing a total of 457 cases: 228 in the experimental group and 229 in the control group. The heterogeneity test showed I² = 0% and a Q test P-value of 0.0001, indicating low inter-study heterogeneity. Using a fixed-effect model, the difference was found to be statistically significant (OR = 2.70, 95% CI [1.63, 4.48], Z = 3.86, P = 0.0001), suggesting that the efficacy of the experimental group was superior to that of the control group (**Supplementary 1**; **Figure 1**).

#### 3.2.2 HAMD-17

Eight studies (13, 17, 21, 22, 24, 26–28, 30) utilized the Hamilton Depression Rating Scale (HAMD-17) as an outcome measure, encompassing a total of 808 cases: 413 in the experimental group and 395 in the control group. The heterogeneity test yielded an I² of 0% and a Q test result of P < 0.00001, indicating low inter-study heterogeneity. The difference was statistically significant using the fixed-effect model (SMD=-0.28,95% CI[-0.42, -0.14], Z=3.92, P<0.0001), indicating that the efficacy of the experimental group was superior to that of the control group (**Supplementary 2**; **Figure 1**).

#### 3.2.3 HAMD-24

Three studies (25, 29, 31) utilized the Hamilton Depression Rating Scale (HAMD-24) as an outcome measure, encompassing a total of 208 cases, with 104 participants in the experimental group and 104 in the control group. The heterogeneity test yielded an I² of 68% and a Q test P-value of 0.01, indicating a high level of heterogeneity among the studies. The difference was statistically significant when applying the random effects model (SMD = -0.64, 95% CI [-1.15, 0.13], Z = 2.47, P = 0.01), suggesting that the efficacy of the experimental group was greater than that of the control group (**Supplementary 3**; **Figure 1**).

Sensitivity analysis indicated that after excluding the study by Gu (2018) (27), the remaining two studies included a total of 150 participants (74 in the intervention group and 76 in the control group). Heterogeneity among the studies was significantly reduced (I² = 23%, P = 0.03), which justified the application of a fixed-effect model (**Table 3A**; Sensitivity analysis). The meta-analysis revealed a statistically significant difference in HAMD-24 scores between the intervention group and the control group (SMD = -0.39, 95% CI [-0.72, -0.07], P = 0.03). The consistency between the fixed-effect and random-effects models further supported the robustness of this finding.

**Table 3:** Sensitivity analysis.

A: Sensitivity analysis results of HAMD-24.				
Eliminated articles	I²(%)	SMD	95%CI	P-valued
Chen 2010 (23)	84	-0.67	[-1.54,0.20]	0.13
Gu 2018	23	-0.39	[-0.72,-0.07]	0.03
Zhang 2010 (30)	41	-0.86	[-1.24,-0.48]	<0.00001
B: Sensitivity analysis results of SDS.				
Eliminated articles	I²(%)	SMD	95%CI	P-valued
Gu 2018	73	-1.50	[-2.26,-0.74]	0.0001
Wang 2015 (25)	98	-3.9	[-7.00,0.83]	0.12
Wang 2023 (28)	96	-3.47	[-6.62,0.31]	0.03
C: Sensitivity analysis results of Adverse reactions.				
Eliminated articles	I²(%)	OR	95%CI	P-valued
Li 2015 (24)	34	0.42	[0.15,1.13]	0.08
Li 2018 (26)	65	0.08	[0.01,0.51]	0.007
Qian 2007 (21)	88	0.15	[0.01,2.05]	0.16
Wang 2023 (28)	89	0.13	[0.01,1.28]	0.08
D: Sensitivity analysis results of KI.				
Eliminated articles	I²(%)	SMD	95%CI	P-valued
Deng 2008 (22)	83	-0.54	[-1.25.0.17]	0.14
Gu 2018	28	-0.23	[-0.51,0.06]	0.12
Liu 2022 (31)	83	-0.46	[-1.19,0.27]	0.22
Zhao 2023	67	-0.65	[-1.18,-0.13]	0.02

#### 3.2.4 SDS

Three studies (23, 25, 26) included a total of 188 cases, with 94 participants in the experimental group and 94 in the control group. The heterogeneity test yielded an I² of 95% and a Q test result of P = 0.004, indicating a high level of heterogeneity among the studies. The difference was statistically significant when analyzed using the random effects model (SMD = -2.64, 95% CI [-4.44, -0.84], Z = 2.83, P = 0.004), suggesting that the efficacy of the experimental group was greater than that of the control group (**Supplementary 4**; **Figure 1**).

Sensitivity analysis was conducted to investigate the sources of heterogeneity and evaluate the stability of the results for the SDS index. As shown in the sensitivity analysis table (**Table 3B**), eliminating individual studies did not reduce the heterogeneity to a low-level range (I² < 50%), indicating that the high heterogeneity among the studies was relatively robust (**Table 3B**; Sensitivity analysis).

### 3.3 Secondary endings

#### 3.3.1 Sexual hormones

Regarding the assessment of serum sex hormone components (FSH, LH, E2) as secondary outcome indicators. Six studies (13, 17, 24, 29–31) assessed follicle-stimulating hormone (FSH) and estradiol (E2) and Five studies (3, 17, 24, 30, 31) assessed luteinizing hormone (LH), FSH and E2 levels totaling 713 cases, 363 in the experimental group and 350 in the control group. FSH levels heterogeneity test I^2^ = 0%,Q test P=0.59, suggesting low inter-study heterogeneity, and the difference was not statistically significant using a fixed-effects model (SMD=-0.04,95% CI[-0.19,-0.11], Z=0.53,P=0.59). (**Supplementary 5**; **Figure 1**) E2 levels heterogeneity test I^2^ = 0%,Q test P=0.89, suggesting low inter-study heterogeneity, and the difference was not statistically significant using a fixed-effects model (SMD=-0.01,95% CI [-0.16, 0.14],Z=0.14,P=0.89). (**Supplementary 5**; **Figure 2**) LH levels totaling 653 cases, 333 in the experimental group and 320 in the control group. heterogeneity test I^2^ = 0%,Q test P=0.86, suggesting low inter-study heterogeneity, and the difference was not statistically significant using a fixed-effects model (SMD = -0.01,95% CI [-0.14, 0.17], Z=0.18, P=0.86).FSH, LH, E2 indicating that the efficacy of the experimental group was not statistically significant compared with that of the control group (**Supplementary 5**; **Figure 3**).

#### 3.3.2 Adverse reactions

Among the studies included in the literature review, four studies (21, 24, 26, 30) reported a total of 414 adverse reactions in patients: 211 cases in the experimental group and 203 cases in the control group. The heterogeneity test yielded an I² of 83% and a Q test P-value of 0.05, indicating high inter-study heterogeneity. The difference in efficacy between the experimental and control groups was not statistically significant when analyzed using the random effects model (OR = 0.16, 95% CI [0.03, 0.98], Z = 1.99, P = 0.05). This suggests that there was no statistically significant difference in efficacy between the experimental group and the control group (**Supplementary 6**; **Figure 1**).

Sensitivity analysis excluding Li (2015) revealed reduced heterogeneity (I² = 34%, P = 0.08) among the remaining three studies (181 interventions vs. 173 controls). Fixed-effect model analysis indicated no statistically significant difference in adverse events (OR = 0.42, 95% CI [-0.15, 1.13], P = 0.08). Further exclusion of Li (2018) resulted in two studies (94 interventions vs. 92 controls) with decreased heterogeneity (I² = 65%, P = 0.007), demonstrating a statistically significant reduction in adverse events for the intervention group (OR = 0.08, 95% CI [0.01, 0.51], P = 0.007). Consistency between fixed-effect and random-effects models supported the robustness of this finding (**Table 3C**; Sensitivity Analysis).

#### 3.3.3 MENQOL

Four studies (17, 23, 24, 30) evaluated the anxiety-relieving effects using the MENQOL scale, encompassing a total of 560 cases: 287 in the experimental group and 273 in the control group. The heterogeneity test yielded I² = 0% and a Q test P-value of 0.003, indicating low heterogeneity among the studies. The difference was statistically significant when applying the fixed-effect model (SMD = -0.25, 95% CI [-0.42, -0.09], Z = 2.98, P = 0.003), suggesting that the efficacy of the experimental group was greater than that of the control group (**Supplementary 7**; **Figure 1**).

#### 3.3.4 KI

Among the studies included in the literature review, four studies (13, 22, 25, 28) reported a total of 250 cases of KI, with 126 cases in the experimental group and 124 cases in the control group. The heterogeneity test yielded an I² of 75% and a Q test P-value of 0.07, indicating high inter-study heterogeneity. The difference in efficacy between the experimental and control groups was not statistically significant when analyzed using the random effects model (SMD = -0.47, 95% CI [-0.98, 0.05], Z = 1.79, P = 0.07). This suggests that there was no statistically significant difference in efficacy between the experimental group and the control group(**Supplementary 8**; **Figure 1**).

Sensitivity analysis indicated that after excluding the study by Gu (2015) (27), the heterogeneity index decreased to I² = 28%, indicating low heterogeneity. However, the result remained statistically non-significant. Conversely, when removing Wang (2023) (13), the heterogeneity index decreased to I² = 67% (still indicating high heterogeneity), but the result became statistically significant (SMD = −0.65, 95% CI[−1.18, −0.13], Z = 2.42, P = 0.02) (**Table 3D**). This suggests that the Wang study had a notable impact on the statistical significance of the results, possibly due to differences in its control group interventions compared to those in other studies (**Table 3D**; Sensitivity analysis).

### 3.4 Subgroup analysis

We conducted subgroup analyses for three categorical indicators: A) the type of acupuncture in the experimental group, B) the type of control group, and C) the acupuncture sites, as shown in **Table 4**.

**Table 4:** Results of the analysis of individual outcome indicators and their subgroups.

Subgroup analysis dimensions		Outcomes		Group	Number of comparison	Total number of participants	WMD/SMD	Effect size		P	Heterogeneity
								95%CI			I²(%)
The type of acupuncture in the experimental group.		HAMD-24		Over Analysis	3	208	SMD=-0.64	[-1.15	-0.13]	0.010	68.00%
				EA	1	90	SMD=-0.24	[-0.66,	0.17]	0.250	NA
				MA	2	118	SMD=-0.87	[-1.36,	-0.37]	0.001	41.00%
		Adverse reactions		Over Analysis	4	414	SMD=0.16	[0.03,	0.98]	0.050	83.00%
				EA	2	288	SMD=0.11	[-0.00,	5.18]	0.260	94.00%
				MA	2	126	SMD=0.19	[0.05,	-0.73]	0.020	0.00%
		FSH		Over Analysis	6	713	SMD=-0.04	[-0.19,	0.11]	0.590	0.00%
				EA	4	583	SMD=0.00	[-0.16,	0.17]	0.970	0.00%
				MA	2	130	SMD=-0.24	[-0.58,	0.11]	0.180	0.00%
		E2		Over Analysis	6	713	SMD=-0.01	[-0.16,	0.14]	0.890	0.00%
				EA	4	583	SMD=-0.04	[-0.20,	0.12]	0.640	0.00%
				MA	2	130	SMD=0.12	[-0.23,	0.46]	0.510	26.00%
		LH		Over Analysis	5	653	SMD=0.01	[-0.14,	0.17]	0.860	0.00%
				EA	4	583	SMD=0.01	[-0.15,	0.18]	0.870	0.00%
				MA	1	70	SMD=0.02	[-0.44,	0.49]	0.920	NA
Control Group Type		HAMD-24		Over Analysis	3	208	SMD=-0.64	[-1.15,	-0.13]	0.010	68.00%
				CHM	2	118	SMD=-0.87	[-1.36,	-0.37]	0.001	41.00%
				other medications	1	90	SMD=0.24	[-0.66,	0.17]	0.250	NA
		Adverse reactions		Over Analysis	4	414	OR=0.16	[0.03,	0.98]	0.050	83.00%
				CHM	1	63	OR=0.32	[0.03,	3.28]	0.340	NA
				other medications	3	351	OR=0.13	[0.01,	1.28]	0.080	89.00%
		KI		Over Analysis	4	250	SMD=-0.47	[-0.98	-0.05]	0.070	75.00%
				CHM	2	122	SMD=-0.85	[-1.53,	-0.16]	0.020	70.00%
				other medications	1	58	SMD=-0.28	[-0.80,	-0.24]	0.290	NA
				SA	1	70	SMD=0.07	[-0.40,	-0.54]	0.780	NA
		FSH		Over Analysis	6	713	SMD=-0.04	[-0.19,	0.11]	0.590	0.00%
				CHM	1	60	SMD=-0.37	[-0.88,	0.14]	0.150	NA
				other medications	4	583	SMD=0.00	[-0.16,	0.17]	0.970	0.00%
				SA	1	70	SMD=-0.12	[-0.59,	0.35]	0.610	NA
		E2		Over Analysis	6	713	SMD=-0.01	[-0.16,	0.14]	0.890	0.00%
				CHM	1	60	SMD=0.34	[-0.17,	0.85]	0.190	NA
				other medications	4	583	SMD=-0.04	[-0.20,	0.12]	0.640	0.00%
				SA	1	70	SMD=-0.07	[-0.54,	0.40]	0.760	NA
		LH		Over Analysis	5	653	SMD=0.01	[-0.14,	0.17]	0.860	0.00%
				other medications	4	583	SMD=0.01	[-0.15,	0.18]	0.870	0.00%
				SA	1	70	SMD=0.02	[-0.44,	0.49]	0.920	NA
Acupuncture sites		SDS		Over Analysis	3	188	SMD=-2.64	[-4.44,	-0.84]	0.004	95.00%
				Simple abdominal acupuncture	1	63	SMD=-1.12	[-1.65,	-0.58]	<0.0001	NA
				Non-simple abdominal acupuncture	2	125	SMD=-3.47	[-6.62,	-0.31]	0.030	96.00%
		Adverse reactions		Over Analysis	4	414	OR=0.16	[0.03,	0.98]	0.050	83.00%
				Simple abdominal acupuncture	1	63	OR=0.32	[0.03,	3.28]	0.340	NA
				Non-simple abdominal acupuncture	3	351	OR=0.13	[0.01,	1.28]	0.080	89.00%
		KI		Over Analysis	4	250	SMD=-0.47	[-0.98,	-0.05]	0.070	75.00%
				Simple abdominal acupuncture	1	58	SMD=-0.28	[-0.80,	-0.24]	0.290	NA
				Non-simple abdominal acupuncture	3	192	SMD=-0.54	[-1.25,	-0.17]	0.140	83.00%

HAMD, the Hamilton Depression Rating Scale; SDS, self-rating depression scale; KI, Kupperman Index; CHM, Chinese herbal medicine; NA, Not Applicable.

#### 3.4.1 Subgroup analysis of HAMD-24

The type of acupuncture used in the experimental group.

The pooled analysis for the EA subgroup revealed no significant difference between the experimental group and the control group (SMD = -0.24, 95% CI [-0.66, 0.17], P = 0.25), and heterogeneity was not applicable. In contrast, the pooled results for the MA subgroup indicated a significant effect favoring the experimental group (SMD = -0.87, 95% CI [-1.36, -0.37], P = 0.0006), with low heterogeneity (I² = 41%). The test for subgroup differences demonstrated a non-significant trend toward a difference (I² = 72.4%, P = 0.06).

Control Group Type

The pooled analysis of the CHM subgroup revealed a significant effect favoring the experimental group (SMD = -0.87, 95% CI [-1.36, -0.37], P = 0.0006), with low heterogeneity (I² = 41%). In the subgroup of other medications, no significant difference was observed between the experimental group and the controls (SMD = -0.24, 95% CI [-0.66, 0.17], P = 0.25), and heterogeneity was not applicable. The test for subgroup differences indicated a trend toward a difference that was not statistically significant (I² = 72.4%, P = 0.06).

#### 3.4.2 Subgroup analysis of SDS

Acupuncture Points

The pooled analysis of the simple abdominal acupuncture subgroup revealed a significant effect favoring the experimental group (SMD = -1.12, 95% CI [-1.65, -0.58], P < 0.0001), with no observed heterogeneity (I² = 0%). In contrast, the pooled results for the non-simple abdominal acupuncture subgroup also indicated a significant effect in favor of the experimental group (SMD = -3.47, 95% CI [-6.62, -0.31], P = 0.03), but exhibited high heterogeneity (I² = 96%). The test for subgroup differences indicated no statistically significant difference (I² = 51.8%, P = 0.15).

#### 3.4.3 Subgroup analysis of adverse reactions

The type of acupuncture used in the experimental group.

The meta-analysis of the EA subgroup, which included two studies, demonstrated significant heterogeneity (I² = 94%). The results from the analysis using a random-effects model indicated no significant difference in efficacy between the EA group and the conventional drug group for the condition (OR = 0.11, 95% CI [0.00, 5.18], P = 0.26). In contrast, the MA subgroup, comprising two studies, showed no statistical heterogeneity (I² = 0%). The results from the fixed-effects model indicated a significant difference in efficacy between the MA group and the conventional medication group for the condition (OR = 0.19, 95% CI [0.05, 0.73], P = 0.02). Furthermore, the test for subgroup differences revealed no statistically significant difference between the subgroups (I² = 0%, P = 0.78).

Acupuncture Points

In the subgroup analysis of simple abdominal acupuncture, the study found no significant difference (OR = 0.32, 95% CI [0.03, 3.28], P = 0.34), and there was no evidence of heterogeneity. In the non-simple abdominal acupuncture subgroup, the pooled results suggested a non-significant trend (OR = 0.13, 95% CI [0.01, 1.28], P = 0.08), but this subgroup exhibited high heterogeneity (I² = 89%). The test for subgroup differences indicated no significant variation (I² = 0%, P = 0.58).

Control Group Type

The results of the subgroup analysis, categorized by the types of control groups, were consistent with those based on the acupuncture sites.

#### 3.4.4 Subgroup analysis of KI

Control Group Type

The pooled analysis for the CHM subgroup demonstrated significantly superior efficacy of the intervention group compared to the control group (SMD = -0.85, 95% CI [-1.53, -0.06], P = 0.02), with moderate heterogeneity (I² = 70%). In the other medicine subgroup, the intervention group showed no significant difference (SMD = -0.28, 95% CI [-0.80, 0.24], P = 0.29), and heterogeneity was not applicable. In the sham acupuncture subgroup, the intervention group also exhibited no significant difference (SMD = 0.07, 95% CI [-0.40, 0.54], P = 0.78), and heterogeneity was not applicable. The overall analysis indicated no significant effect of the intervention (SMD = -0.47, 95% CI [-0.98, 0.05], P = 0.07), with significant heterogeneity (I² = 75%). Subgroup differences were not statistically significant (I² = 57.2%, P = 0.10).

Acupuncture Points

The pooled analysis of the simple abdominal acupuncture subgroup revealed no significant difference in efficacy between the intervention group and the control group (SMD = -0.28, 95% CI [-0.80, 0.24], P = 0.29), and heterogeneity was not applicable. In the non-simple abdominal acupuncture subgroup, the intervention group exhibited a non-significant trend toward superiority (SMD = -0.54, 95% CI [-1.25, 0.17], P = 0.14) with high heterogeneity (I² = 83%). The overall analysis indicated no significant effect of the intervention (SMD = -0.47, 95% CI [-0.98, 0.05], P = 0.07), but significant heterogeneity was present (I² = 75%). Subgroup differences were not statistically significant (I² = 0%, P = 0.57).

#### 3.4.5 Subgroup analysis of sexual hormones

In the subgroup analyses of follicle-stimulating hormone (FSH), estradiol (E2), and luteinizing hormone (LH) in the meta-analysis, regardless of whether the subgrouping was based on the type of acupuncture in the experimental group or the type of control group, the heterogeneity was found to be 0%, and the p-values were all greater than 0.05. The results of the fixed-effects modeling indicated that the efficacy of the experimental group was not statistically significant when compared to that of the control group.

## 4 Discussion

### 4.1 Main findings

This systematic review presents a meta-analysis evaluating the efficacy of acupuncture for menopausal depressive disorder (MDD). Following PRISMA guidelines, our findings indicate that acupuncture significantly outperforms control interventions in improving clinical effectiveness rates (OR=2.70, 95% CI [1.63, 4.48], P=0.0001) and in reducing depressive symptoms, as evidenced by HAMD-17 (SMD=-0.28, P<0.0001) and HAMD-24 scores (post-sensitivity SMD=-0.39, P=0.03). Notably, acupuncture also improved quality of life, as measured by the Menopause Quality of Life Questionnaire (MENQOL) (SMD=-0.25, P=0.003), although its effects on sex hormones (FSH, LH, E2) were not statistically significant (P>0.05). The safety profiles were comparable between groups (OR=0.16, P=0.05); however, sensitivity analysis revealed a reduction in adverse events in the acupuncture group after excluding outlier studies (OR=0.49, P=0.03). In conclusion, the ins is a beneficial intervention MDD.

Acupuncture, as a non-pharmacological intervention, avoids many potential side effects associated with drug therapies and offers a novel treatment approach for patients experiencing menopausal depression. Among the 13 studies included in this review, 11 (84.6%) were conducted after 2010, indicating a growing trend in acupuncture research in recent years. This trend reflects a shift in current clinical needs: while existing guidelines recommend antidepressants and psychotherapy as first-line treatments (11), conventional medicine primarily utilizes estrogen replacement therapy, oral antidepressants, or a combination of both. Although these therapies have demonstrated proven efficacy for menopausal depressive disorder (MDD), long-term use may increase the risks of breast cancer, endometrial cancer, and cardiovascular diseases (32–34), adversely affecting patients’ quality of life and reducing medication compliance. Given that menopause represents a high-risk period for depressive episodes in women, intervention studies targeting this population hold significant clinical importance.

### 4.2 Analysis of sources of heterogeneity and discussion of results from subgroup analysis

#### 4.2.1 HAMD-24

The heterogeneity observed in the HAMD-24 outcomes may primarily stem from significant variations in acupoint selection across trials and limitations in sample size. Although subgroup analysis isolated the study by Zhang (2010) (30) due to discrepancies in control group design, their findings nonetheless demonstrated the superior efficacy of electroacupuncture (EA) over conventional pharmacotherapy in alleviating core HAMD-24 factors such as anxiety/somatization and cognitive impairment (P < 0.01) (30), suggesting symptom-specific therapeutic advantages of EA. Sensitivity analysis revealed a marked reduction in heterogeneity (I² decreased from 68% to 23% following the exclusion of Gu et al. (2022) (27). Upon reviewing the entire text, we found that this study (27) employed distinct acupoints, notably the “Thirteen Ghost Points” (e.g., GV26 and LU11), which are hypothesized to modulate the neuro-endocrine-immune network and rebalance neurotransmitter levels. In contrast, the other two trials (23, 30) utilized traditional acupoints associated with visceral regulation (e.g., GV20 and SP6), focusing on neuroendocrine system modulation and holistic functional improvement through endocrine balance and monoaminergic enhancement. These discrepancies in therapeutic mechanisms—rooted in divergent acupoint selection—likely contributed to the variability in outcomes. Furthermore, the limited number of included studies (n=3) and small sample sizes may have exacerbated methodological heterogeneity. Future investigations should prioritize standardized acupoint protocols and larger cohorts to minimize confounding factors and enhance the generalizability of results.

#### 4.2.2 SDS

The sensitivity analysis of the SDS indicates that the high heterogeneity observed was relatively stable and not solely attributable to any single study. Notably, after excluding Gu 2018 (27), the heterogeneity decreased from 95% to 75%, yet it remained high. This suggests that the elevated heterogeneity may not be due to bias from a single study, but rather could stem from small sample sizes or other contributing factors. It is also noteworthy that the three studies (25, 27, 28) exhibiting high heterogeneity all utilized a combination of acupuncture and medication, in contrast to the control group, which relied solely on traditional Chinese medicine. Despite the persistent heterogeneity, the statistical significance of the results was partially maintained, indicating that the conclusion—that the experimental group demonstrated greater efficacy than the control group—exhibited a certain degree of stability. This implies that, even in the presence of heterogeneity, the overall trend of the experimental group showing superior efficacy compared to the control group remained consistent across the analyses. However, the high heterogeneity still necessitates caution in interpreting the results and underscores the need for further research to elucidate the underlying reasons.

#### 4.2.3 Adverse reactions

The analysis of adverse reactions shows notable differences between electroacupuncture (EA) and manual acupuncture (MA), which are closely related to the nature of these treatments. EA uses electrical currents to activate acupoints, and while factors like intensity and frequency can be modified, inconsistencies in these parameters across various studies can affect treatment effectiveness and increase variability. On the other hand, manual acupuncture depends on the techniques used by the practitioner. Although there are individual differences in technique, it allows for more flexibility and is not constrained by fixed parameters, leading to less variability. Additionally, variations in patients’ sensitivity to EA’s electrical stimulation—such as discomfort from excessive stimulation—can further increase variability. In contrast, the gentler needle stimulation of manual acupuncture is more adaptable and tends to result in fewer adverse reactions.

#### 4.2.4 KI

The differences seen in the KI scale could be due to the varying acupuncture locations and the types of control groups utilized. In the research by Gu et al., the acupuncture points were chosen based on Sun Simiao’s Thirteen Ghost Points, which specifically help in emotional regulation. This approach contrasts with the other three studies, which focused on acupuncture points aimed at balancing Qi and blood, harmonizing the zang-fu organs, and addressing menopausal symptoms in general. When Gu et al.’s study is excluded, the other three studies show more uniformity in both the acupuncture points used and their theoretical underpinnings, leading to a decrease in heterogeneity. Subgroup analysis indicates that when the control group involves medication, the intervention group demonstrates better efficacy. However, no statistical significance is found when comparing the acupuncture group with the sham acupuncture group. Further analysis reveals that the study by Zhao et al. (2023) focused on a single condition and did not cover somatic symptoms included in the KI scale, such as vasomotor symptoms, leading to insignificant improvements in KI. In contrast, the other three studies directly targeted core symptoms assessed by the KI scale, such as hot flashes and physical discomfort, and enhanced efficacy through multi-target regulation (acupuncture plus herbal medicine), indicating that the alignment of intervention measures with the KI assessment and the use of integrated treatment approaches are crucial factors influencing efficacy.

#### 4.2.5 Sexual hormones

Although the forest plots for FSH, E2, and LH demonstrated negligible heterogeneity (I² = 0%), subgroup analyses were conducted to investigate whether potential biases in the included studies influenced these outcomes. Subgroup stratification based on acupuncture type (e.g., MA vs. EA) and control group interventions (e.g., CHM vs. other medications) consistently revealed no statistically significant differences between the intervention and control groups across all three hormonal markers (P > 0.05). These findings preliminarily suggest that the lack of intergroup differences is unlikely attributable to variations in acupuncture protocols or control group designs. Further exploration is warranted to elucidate the underlying mechanisms contributing to the nonsignificant outcomes.

It has been shown that fluctuations in FSH and LH may lead to changes in hot flashes, bones, vascular endothelium, atherosclerosis, and lipid metabolism in women, which in turn affects quality of life (35). Although depression is a common clinical condition in menopausal women, not all women develop depression, and women with significant hormonal fluctuations and sensitivity to hormonal changes are more likely to develop depression (36, 37). According to second-generation cognitive theory, environment, physiology, and mood are recognized as important factors influencing cognitive functioning, which, in turn, directly affects the development of depression (38, 39). The dominant factor in menopausal depression is changes in cognitive functioning, influenced by changes in hormones, clinical symptoms and quality of life. This influence may dominate the onset and progression of depression. Although the beneficial effects of acupuncture on menopausal depression may be related to the modulation of sex hormones, this hypothesis still needs to be tested by a more rigorous experimental design.

There are several reasons why no significant differences were observed in FSH, E2, and LH levels between the intervention and control groups. First, regarding efficacy equivalence, EA, Western medications (hormone replacement + antidepressants), and traditional Chinese medicine all regulate sex hormone levels with comparable effects, showing no statistical differences. The direct impact of Western drugs (e.g., fluoxetine, nilestriol) on hormone levels may counteract intergroup differences (23, 30). Second, regarding non-hormone-dependent mechanisms, EA may improve depression by regulating neurotransmitters (such as 5-HT, NE) or brain neural circuits (HPA axis) rather than directly acting on hormones (15, 24, 26). Acupuncture may alleviate depression indirectly by improving quality of life (MENQOL), sleep, or anxiety symptoms, with hormones serving only as “initiating factors” (13, 17). Third, due to physiological irreversibility, the decline in E2 and elevation in FSH/LH caused by perimenopausal ovarian dysfunction are natural processes that interventions cannot reverse (13, 26). Traditional Chinese medicine theory emphasizes that acupuncture helps establish a new balance through “harmonizing yin and yang” rather than opposing physiological trends (13, 40). Fourth, limitations in study design, such as small sample sizes or short follow-up periods (e.g., 8–12 weeks), make it difficult to capture subtle hormonal changes (17, 23). Multicenter trials or differences in menopausal stages (early/late transition) may lead to data dispersion in hormone levels (26), while limited hormone detection time points (e.g., only baseline and post-treatment) may miss fluctuating effects (13). Finally, mediating variable effects suggest that hormonal fluctuations induce depression indirectly by reducing quality of life (e.g., hot flashes, insomnia) rather than through direct associations (17), and the placebo effect of sham acupuncture may partially offset intergroup differences in hormones (13).

### 4.3 Research strengths and limitations

This meta-analysis presents several key strengths that enhance its scientific rigor and translational value. First, by extending systematic database searches through 2025, it incorporates the most up-to-date evidence on menopausal depression, surpassing prior meta-analyses (41, 42) that typically capped data retrieval at 2021. This temporal expansion ensures the inclusion of recent advancements in diagnostic criteria, intervention modalities, and outcome measures, providing a contemporary synthesis of the evolving field. Second, methodological quality was prioritized using the JADAD scale, with only moderate-to-high-quality studies (tu on the 5-point scale) included. This effectively minimizes bias from low-quality designs (e.g., non-randomized trials, unclear blinding). This rigorous selection criterion strengthens the reliability of pooled estimates compared to earlier analyses with heterogeneous study quality. Third, the study broadens the traditional focus on perimenopausal depression to encompass the entire menopausal spectrum, including perimenopausal, menopausal, and postmenopausal stages. This inclusive approach captures the diverse presentation of depression across reproductive aging phases, aligning with clinical guidelines that recognize menopausal depression as a multistage condition. Collectively, these strengths position the analysis as a robust, evidence-driven resource, combining temporal relevance, methodological rigor, and analytical depth to inform clinical practice and future research in menopausal mental health.

This meta-analysis has several limitations. First, the relatively small number of included trials (n = 13) and participants (N = 1,293) may reduce statistical power and limit the generalizability of the findings.

Second, despite subgroup analyses stratified by acupuncture sites, significant heterogeneity in intervention protocols (e.g., electroacupuncture parameters, needle retention time) and control group designs (e.g., sham acupuncture versus pharmacotherapy) may obscure true effect sizes. This variability likely reflects divergent biological responses to different therapeutic modalities rather than random error, complicating the interpretation of pooled results.

## 5 Conclusions

This systematic review indicates that acupuncture is a safe and effective non-drug treatment for reducing depressive symptoms during menopause and enhancing quality of life. While acupuncture did not significantly change sex hormone levels, its positive effects are likely due to non-hormonal processes, such as the regulation of neurotransmitters and the modulation of the neuroendocrine system. However, the review has limitations, including a small number of studies, methodological differences (like variations in acupoint selection and stimulation techniques), and small participant groups, which may limit the applicability of the results. Future studies should focus on large, standardized trials that include objective biomarkers and long-term follow-up to better understand the mechanisms involved and improve clinical practices, ultimately offering safer and more varied treatment options for menopausal depression.

## Data Availability

The original contributions presented in the study are included in the article/**Supplementary Material**. Further inquiries can be directed to the corresponding author.

## Supplementary material

Supplementary 1 Clinical effectiveness rate.

Supplementary 2 HAMD-17.

Supplementary 3 HAMD-24.

Supplementary 4 SDS.

Supplementary 5 Sexual hormones.

Supplementary 6 Adverse reactions.

Supplementary 7 MENQOL.

Supplementary 8 KI.

Supplementary 9 Search strategy.
