# Supplementary material for: Effect of acupuncture on menopausal depressive disorder and serum hormone levels: a systematic review and meta-analysis
Source: Front Psychiatry. 2025 Jul 14;16:1591389. doi: 10.3389/fpsyt.2025.1591389 (PMC12301320; doi:10.3389/fpsyt.2025.1591389)

Supplementary 1

Figure 1: The forest plot of clinical effectiveness rate

Figure 2: The forest plot of clinical effectiveness ratesub group analysis （The type of acupuncture in the experimental group）

Acupuncture sites

Figure 3: The forest plot of clinical effectiveness ratesub group analysis （Control Group Type）

Figure 4: The forest plot of clinical effectiveness ratesub group analysis （Acupuncture sites）

1

	Experimental		Control			Odds Ratio
Study or Subgroup	Events	Total	Events	Total	Weight	M-H, Fixed, 95% CI
Chen 2010	29	30	24	30	4.2%	7.25 [0.82, 64.46]
Deng 2008	26	29	22	29	11.8%	2.76 [0.64, 11.96]
Gu 2018	27	30	22	28	11.8%	2.45 [0.55, 10.96]
Liu 2022	29	32	23	32	11.2%	3.78 [0.92, 15.60]
Wang 2010	21	30	15	30	23.4%	2.33 [0.81, 6.73]
Wang 2015	30	33	23	34	10.7%	4.78 [1.19, 19.15]
Zhang 2010	38	44	39	46	27.0%	1.14 [0.35, 3.69]
**Total (95% CI)**		**228**		**229**	**100.0%**	**2.70 [1.63, 4.48]**
Total events	200		168			

Heterogeneity: $Chi^2 = 3.82$, df = 6 (P = 0.70); $I^2 = 0\%$

Test for overall effect: Z = 3.86 (P = 0.0001)

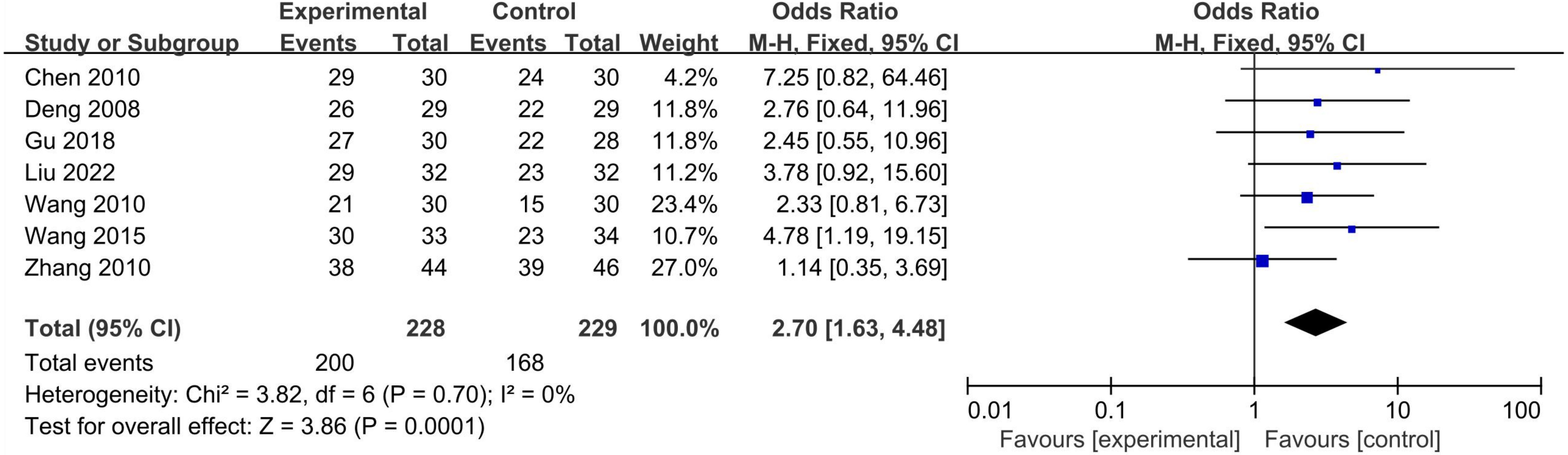

2

Study or Subgroup	Experimental Events	Experimental Total	Control Events	Control Total	Weight	Odds Ratio M-H, Fixed, 95% CI
**3.3.1 EA**						
Zhang 2010	38	44	39	46	27.0%	1.14 [0.35, 3.69]
**Subtotal (95% CI)**		**44**		**46**	**27.0%**	**1.14 [0.35, 3.69]**
Total events	38		39			
Heterogeneity: Not applicable						
Test for overall effect: Z = 0.21 (P = 0.83)						
**3.3.2 MA**						
Chen 2010	29	30	24	30	4.2%	7.25 [0.82, 64.46]
Deng 2008	26	29	22	29	11.8%	2.76 [0.64, 11.96]
Gu 2018	27	30	22	28	11.8%	2.45 [0.55, 10.96]
Liu 2022	29	32	23	32	11.2%	3.78 [0.92, 15.60]
Wang 2010	21	30	15	30	23.4%	2.33 [0.81, 6.73]
Wang 2015	30	33	23	34	10.7%	4.78 [1.19, 19.15]
**Subtotal (95% CI)**		**184**		**183**	**73.0%**	**3.28 [1.86, 5.79]**
Total events	162		129			
Heterogeneity: Chi² = 1.42, df = 5 (P = 0.92); I² = 0%						
Test for overall effect: Z = 4.11 (P < 0.0001)						
**Total (95% CI)**		**228**		**229**	**100.0%**	**2.70 [1.63, 4.48]**
Total events	200		168			
Heterogeneity: Chi² = 3.82, df = 6 (P = 0.70); I² = 0%						
Test for overall effect: Z = 3.86 (P = 0.0001)						
Test for subgroup differences: Chi² = 2.52, df = 1 (P = 0.11), I² = 60.4%						

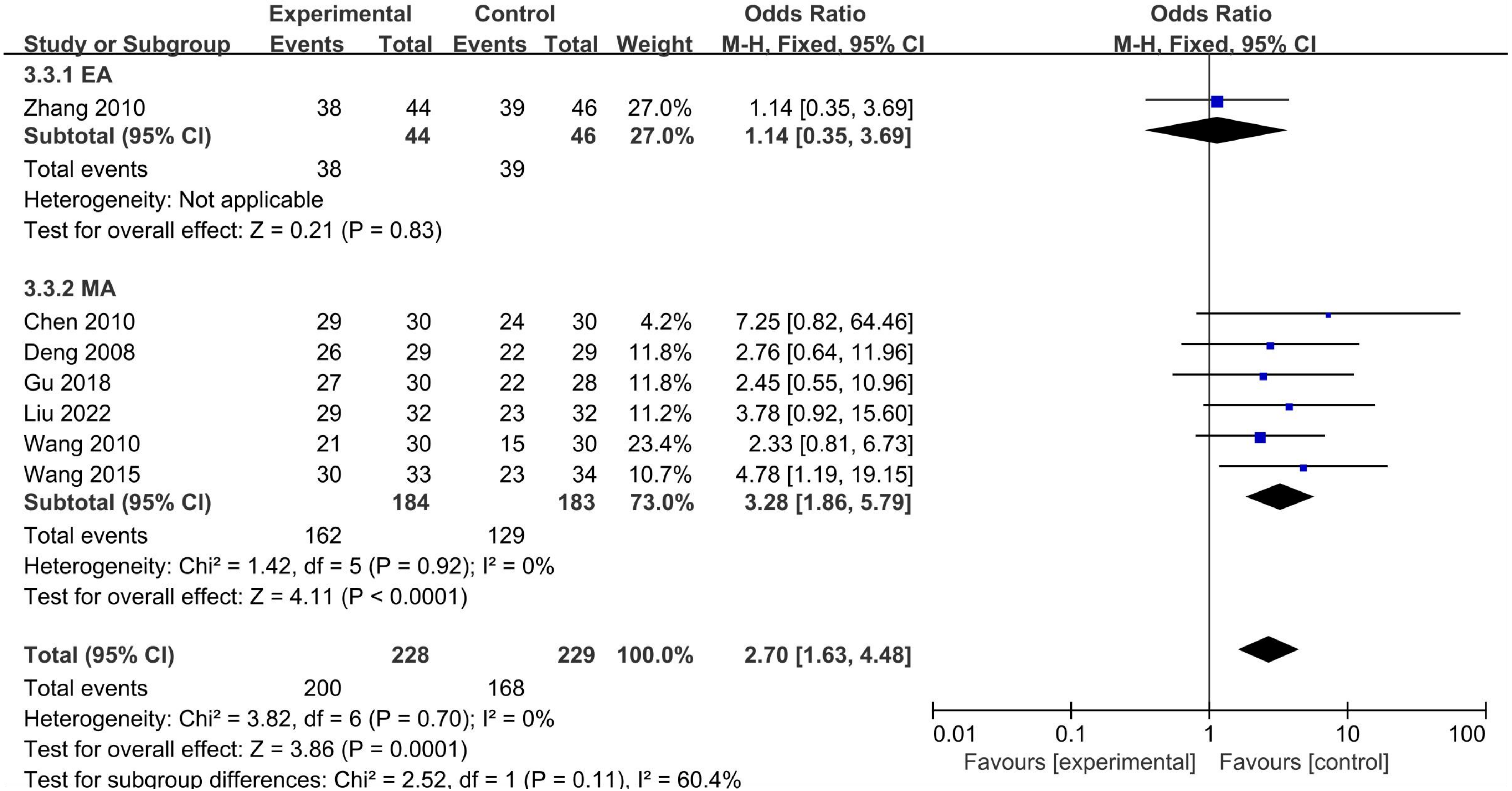

3

Study or Subgroup	Experimental Events	Experimental Total	Control Events	Control Total	Weight	Odds Ratio M-H, Fixed, 95% CI
**3.5.1 CHM**						
Chen 2010	29	30	24	30	4.2%	7.25 [0.82, 64.46]
Gu 2018	27	30	22	28	11.8%	2.45 [0.55, 10.96]
Liu 2022	29	32	23	32	11.2%	3.78 [0.92, 15.60]
Wang 2015	30	33	23	34	10.7%	4.78 [1.19, 19.15]
**Subtotal (95% CI)**		**125**		**124**	**37.8%**	**4.03 [1.88, 8.66]**
Total events	115		92			
Heterogeneity: Chi² = 0.77, df = 3 (P = 0.86); I² = 0%						
Test for overall effect: Z = 3.57 (P = 0.0004)						
**3.5.2 other medications**						
Deng 2008	26	29	22	29	11.8%	2.76 [0.64, 11.96]
Wang 2010	21	30	15	30	23.4%	2.33 [0.81, 6.73]
Zhang 2010	38	44	39	46	27.0%	1.14 [0.35, 3.69]
**Subtotal (95% CI)**		**103**		**105**	**62.2%**	**1.89 [0.95, 3.77]**
Total events	85		76			
Heterogeneity: Chi² = 1.12, df = 2 (P = 0.57); I² = 0%						
Test for overall effect: Z = 1.82 (P = 0.07)						
**Total (95% CI)**		**228**		**229**	**100.0%**	**2.70 [1.63, 4.48]**
Total events	200		168			
Heterogeneity: Chi² = 3.82, df = 6 (P = 0.70); I² = 0%						
Test for overall effect: Z = 3.86 (P = 0.0001)						
Test for subgroup differences: Chi² = 2.07, df = 1 (P = 0.15), I² = 51.7%						

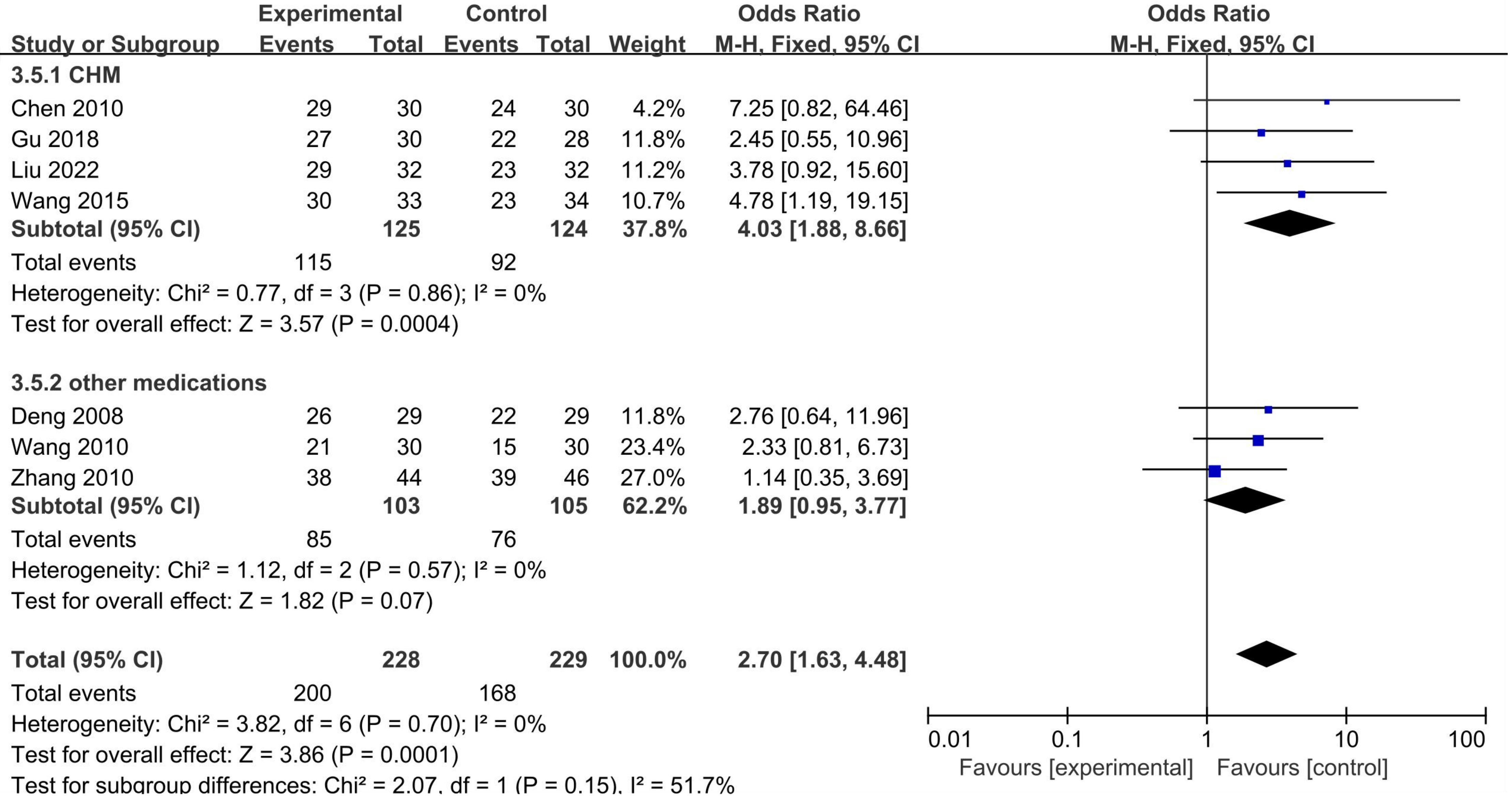

4

Study or Subgroup	Experimental Events	Experimental Total	Control Events	Control Total	Weight	Odds Ratio M-H, Fixed, 95% CI
**3.4.1 Simple abdominal acupuncture**						
Deng 2008	26	29	22	29	11.8%	2.76 [0.64, 11.96]
Wang 2010	30	33	23	34	10.7%	4.78 [1.19, 19.15]
**Subtotal (95% CI)**		**62**		**63**	**22.5%**	**3.72 [1.37, 10.13]**
Total events	56		45			
Heterogeneity: Chi² = 0.29, df = 1 (P = 0.59); I² = 0%						
Test for overall effect: Z = 2.57 (P = 0.01)						
**3.4.2 Non - simple abdominal acupuncture**						
Chen 2010	29	30	24	30	4.2%	7.25 [0.82, 64.46]
Gu 2018	27	30	22	28	11.8%	2.45 [0.55, 10.96]
Liu 2022	29	32	23	32	11.2%	3.78 [0.92, 15.60]
Wang 2015	21	30	15	30	23.4%	2.33 [0.81, 6.73]
Zhang 2010	38	44	39	46	27.0%	1.14 [0.35, 3.69]
**Subtotal (95% CI)**		**166**		**166**	**77.5%**	**2.41 [1.34, 4.33]**
Total events	144		123			
Heterogeneity: Chi² = 2.93, df = 4 (P = 0.57); I² = 0%						
Test for overall effect: Z = 2.94 (P = 0.003)						
**Total (95% CI)**		**228**		**229**	**100.0%**	**2.70 [1.63, 4.48]**
Total events	200		168			
Heterogeneity: Chi² = 3.82, df = 6 (P = 0.70); I² = 0%						
Test for overall effect: Z = 3.86 (P = 0.0001)						
Test for subgroup differences: Chi² = 0.54, df = 1 (P = 0.46), I² = 0%						

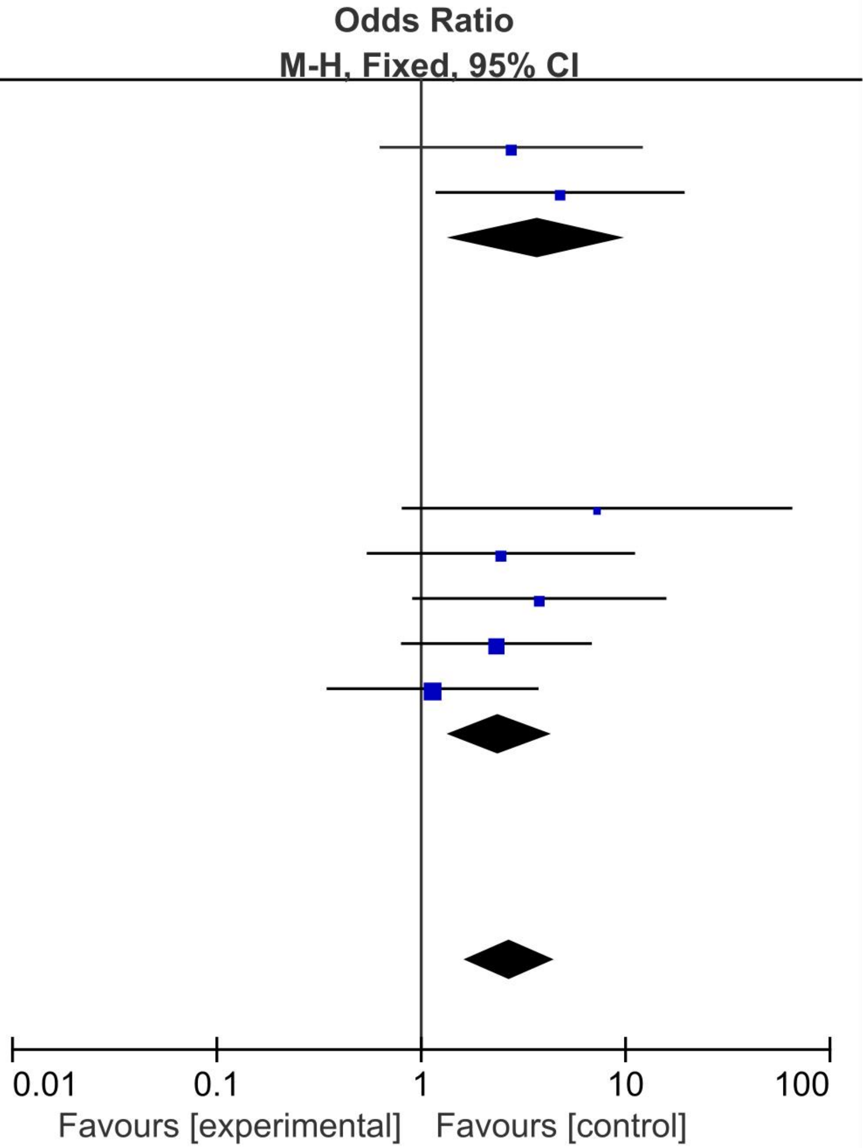

Supplement: Supplementary 1 — Clinical effectiveness rate. [file SupplementaryFile1.zip › Supplementary material 1.PDF]
