# Supplementary material for: Effect of acupuncture on menopausal depressive disorder and serum hormone levels: a systematic review and meta-analysis
Source: Front Psychiatry. 2025 Jul 14;16:1591389. doi: 10.3389/fpsyt.2025.1591389 (PMC12301320; doi:10.3389/fpsyt.2025.1591389)
Supplement: Supplementary 1 — Clinical effectiveness rate. [file SupplementaryFile1.zip › Supplementary table 4.DOCX]

| Table 4：Results of the analysis of individual outcome indicators and their subgroups. | | | | | | | | | | | |
| --- | --- | --- | --- | --- | --- | --- | --- | --- | --- | --- | --- |
| Subgroup Analysis  Dimensions | | Outcomes | | Group | Number of | Total number of | WMD/SMD | Effect Size | | P | Heterogeneity |
|  |  |  |  |  | comparison | participants |  | 95%CI | |  | I²(%) |
| The type of  acupuncture in the  experimental group. | | HAMD-24 | | Over Analysis | 3 | 208 | SMD=-0.64 | [-1.15 | -0.13] | 0.010 | 68.00% |
|  |  |  |  |  |  |  |  |  |  |  |  |
|  |  |  |  | EA | 1 | 90 | SMD=-0.24 | [-0.66, | 0.17] | 0.250 | NA |
|  |  |  |  |  |  |  |  |  |  |  |  |
|  |  |  |  | MA | 2 | 118 | SMD=-0.87 | [-1.36, | -0.37] | 0.001 | 41.00% |
|  |  |  |  |  |  |  |  |  |  |  |  |
|  |  | Adverse reactions | | Over Analysis | 4 | 414 | SMD=0.16 | [0.03, | 0.98] | 0.050 | 83.00% |
|  |  |  |  |  |  |  |  |  |  |  |  |
|  |  |  |  | EA | 2 | 288 | SMD=0.11 | [-0.00, | 5.18] | 0.260 | 94.00% |
|  |  |  |  |  |  |  |  |  |  |  |  |
|  |  |  |  | MA | 2 | 126 | SMD=0.19 | [0.05, | -0.73] | 0.020 | 0.00% |
|  |  |  |  |  |  |  |  |  |  |  |  |
|  |  | FSH | | Over Analysis | 6 | 713 | SMD=-0.04 | [-0.19, | 0.11] | 0.590 | 0.00% |
|  |  |  |  |  |  |  |  |  |  |  |  |
|  |  |  |  | EA | 4 | 583 | SMD=0.00 | [-0.16, | 0.17] | 0.970 | 0.00% |
|  |  |  |  |  |  |  |  |  |  |  |  |
|  |  |  |  | MA | 2 | 130 | SMD=-0.24 | [-0.58, | 0.11] | 0.180 | 0.00% |
|  |  |  |  |  |  |  |  |  |  |  |  |
|  |  | E2 | | Over Analysis | 6 | 713 | SMD=-0.01 | [-0.16, | 0.14] | 0.890 | 0.00% |
|  |  |  |  |  |  |  |  |  |  |  |  |
|  |  |  |  | EA | 4 | 583 | SMD=-0.04 | [-0.20, | 0.12] | 0.640 | 0.00% |
|  |  |  |  |  |  |  |  |  |  |  |  |
|  |  |  |  | MA | 2 | 130 | SMD=0.12 | [-0.23, | 0.46] | 0.510 | 26.00% |
|  |  |  |  |  |  |  |  |  |  |  |  |
|  |  | LH | | Over Analysis | 5 | 653 | SMD=0.01 | [-0.14, | 0.17] | 0.860 | 0.00% |
|  |  |  |  |  |  |  |  |  |  |  |  |
|  |  |  |  | EA | 4 | 583 | SMD=0.01 | [-0.15, | 0.18] | 0.870 | 0.00% |
|  |  |  |  |  |  |  |  |  |  |  |  |
|  |  |  |  | MA | 1 | 70 | SMD=0.02 | [-0.44, | 0.49] | 0.920 | NA |
|  |  |  |  |  |  |  |  |  |  |  |  |
| Control Group Type | | HAMD-24 | | Over Analysis | 3 | 208 | SMD=-0.64 | [-1.15, | -0.13] | 0.010 | 68.00% |
|  |  |  |  |  |  |  |  |  |  |  |  |
|  |  |  |  | CHM | 2 | 118 | SMD=-0.87 | [-1.36, | -0.37] | 0.001 | 41.00% |
|  |  |  |  |  |  |  |  |  |  |  |  |
|  |  |  |  | other medications | 1 | 90 | SMD=0.24 | [-0.66, | 0.17] | 0.250 | NA |
|  |  |  |  |  |  |  |  |  |  |  |  |
|  |  | Adverse reactions | | Over Analysis | 4 | 414 | OR=0.16 | [0.03, | 0.98] | 0.050 | 83.00% |
|  |  |  |  |  |  |  |  |  |  |  |  |
|  |  |  |  | CHM | 1 | 63 | OR=0.32 | [0.03, | 3.28] | 0.340 | NA |
|  |  |  |  |  |  |  |  |  |  |  |  |
|  |  |  |  | other medications | 3 | 351 | OR=0.13 | [0.01, | 1.28] | 0.080 | 89.00% |
|  |  |  |  |  |  |  |  |  |  |  |  |
|  |  | KI | | Over Analysis | 4 | 250 | SMD=-0.47 | [-0.98 | -0.05] | 0.070 | 75.00% |
|  |  |  |  |  |  |  |  |  |  |  |  |
|  |  |  |  | CHM | 2 | 122 | SMD=-0.85 | [-1.53, | -0.16] | 0.020 | 70.00% |
|  |  |  |  |  |  |  |  |  |  |  |  |
|  |  |  |  | other medications | 1 | 58 | SMD=-0.28 | [-0.80, | -0.24] | 0.290 | NA |
|  |  |  |  |  |  |  |  |  |  |  |  |
|  |  |  |  | SA | 1 | 70 | SMD=0.07 | [-0.40, | -0.54] | 0.780 | NA |
|  |  |  |  |  |  |  |  |  |  |  |  |
|  |  | FSH | | Over Analysis | 6 | 713 | SMD=-0.04 | [-0.19, | 0.11] | 0.590 | 0.00% |
|  |  |  |  |  |  |  |  |  |  |  |  |
|  |  |  |  | CHM | 1 | 60 | SMD=-0.37 | [-0.88, | 0.14] | 0.150 | NA |
|  |  |  |  |  |  |  |  |  |  |  |  |
|  |  |  |  | other medications | 4 | 583 | SMD=0.00 | [-0.16, | 0.17] | 0.970 | 0.00% |
|  |  |  |  |  |  |  |  |  |  |  |  |
|  |  |  |  | SA | 1 | 70 | SMD=-0.12 | [-0.59, | 0.35] | 0.610 | NA |
|  |  |  |  |  |  |  |  |  |  |  |  |
|  |  | E2 | | Over Analysis | 6 | 713 | SMD=-0.01 | [-0.16, | 0.14] | 0.890 | 0.00% |
|  |  |  |  |  |  |  |  |  |  |  |  |
|  |  |  |  | CHM | 1 | 60 | SMD=0.34 | [-0.17, | 0.85] | 0.190 | NA |
|  |  |  |  |  |  |  |  |  |  |  |  |
|  |  |  |  | other medications | 4 | 583 | SMD=-0.04 | [-0.20, | 0.12] | 0.640 | 0.00% |
|  |  |  |  |  |  |  |  |  |  |  |  |
|  |  |  | | SA | 1 | 70 | SMD=-0.07 | [-0.54, | 0.40] | 0.760 | NA |
|  |  |  |  |  |  |  |  |  |  |  |  |
|  |  | LH | | Over Analysis | 5 | 653 | SMD=0.01 | [-0.14, | 0.17] | 0.860 | 0.00% |
|  |  |  |  |  |  |  |  |  |  |  |  |
|  |  |  |  | other medications | 4 | 583 | SMD=0.01 | [-0.15, | 0.18] | 0.870 | 0.00% |
|  |  |  |  |  |  |  |  |  |  |  |  |
|  |  |  |  | SA | 1 | 70 | SMD=0.02 | [-0.44, | 0.49] | 0.920 | NA |
|  |  |  |  |  |  |  |  |  |  |  |  |
| Acupuncture sites | | SDS | | Over Analysis | 3 | 188 | SMD=-2.64 | [-4.44, | -0.84] | 0.004 | 95.00% |
|  |  |  |  |  |  |  |  |  |  |  |  |
|  |  |  |  | Simple abdominal acupuncture | 1 | 63 | SMD=-1.12 | [-1.65, | -0.58] | <0.0001 | NA |
|  |  |  |  |  |  |  |  |  |  |  |  |
|  |  |  |  | Non-simple abdominal acupuncture | 2 | 125 | SMD=-3.47 | [-6.62, | -0.31] | 0.030 | 96.00% |
|  |  |  |  |  |  |  |  |  |  |  |  |
|  |  | Adverse reactions | | Over Analysis | 4 | 414 | OR=0.16 | [0.03, | 0.98] | 0.050 | 83.00% |
|  |  |  |  |  |  |  |  |  |  |  |  |
|  |  |  |  | Simple abdominal acupuncture | 1 | 63 | OR=0.32 | [0.03, | 3.28] | 0.340 | NA |
|  |  |  |  |  |  |  |  |  |  |  |  |
|  |  |  |  | Non-simple abdominal acupuncture | 3 | 351 | OR=0.13 | [0.01, | 1.28] | 0.080 | 89.00% |
|  |  |  |  |  |  |  |  |  |  |  |  |
|  |  | KI | | Over Analysis | 4 | 250 | SMD=-0.47 | [-0.98, | -0.05] | 0.070 | 75.00% |
|  |  |  |  |  |  |  |  |  |  |  |  |
|  |  |  |  | Simple abdominal acupuncture | 1 | 58 | SMD=-0.28 | [-0.80, | -0.24] | 0.290 | NA |
|  |  |  |  |  |  |  |  |  |  |  |  |
|  |  |  |  | Non-simple abdominal acupuncture | 3 | 192 | SMD=-0.54 | [-1.25, | -0.17] | 0.140 | 83.00% |
|  |  |  |  |  |  |  |  |  |  |  |  |
| HAMD：the Hamilton Depression Rating Scale SDS:self-rating depression scale  KI:Kupperman Index CHM:Chinese herbal medicine NA:Not Applicable | | | | | | | | | | | |
