# Supplementary material for: Effect of acupuncture on menopausal depressive disorder and serum hormone levels: a systematic review and meta-analysis
Source: Front Psychiatry. 2025 Jul 14;16:1591389. doi: 10.3389/fpsyt.2025.1591389 (PMC12301320; doi:10.3389/fpsyt.2025.1591389)
Supplement: Supplementary 1 — Clinical effectiveness rate. [file SupplementaryFile1.zip › Supplementary table 3.DOCX]

| Table3:Sensitivity analysis | | | | |
| --- | --- | --- | --- | --- |
| A:Sensitivity analysis results of HAMD-24. | | | | |
| Eliminated articles | I²(%) | SMD | 95%CI | P-valued |
| Chen 2010 | 84 | -0.67 | [-1.54,0.20] | 0.13 |
| Gu 2018 | 23 | -0.39 | [-0.72,-0.07] | 0.03 |
| Zhang 2010 | 41 | -0.86 | [-1.24,-0.48] | ＜0.00001 |
| B:Sensitivity analysis results of SDS. | | | | |
| Eliminated articles | I²(%) | SMD | 95%CI | P-valued |
| Gu 2018 | 73 | -1.50 | [-2.26,-0.74] | 0.0001 |
| Wang 2015 | 98 | -3.9 | [-7.00,0.83] | 0.12 |
| Wang 2023 | 96 | -3.47 | [-6.62,0.31] | 0.03 |
| C:Sensitivity analysis results of Adverse reactions. | | | | |
| Eliminated articles | I²(%) | OR | 95%CI | P-valued |
| Li 2015 | 34 | 0.42 | [0.15,1.13] | 0.08 |
| Li 2018 | 65 | 0.08 | [0.01,0.51] | 0.007 |
| Qian 2007 | 88 | 0.15 | [0.01,2.05] | 0.16 |
| Wang 2023 | 89 | 0.13 | [0.01,1.28] | 0.08 |
| D:Sensitivity analysis results of KI. | | | | |
| Eliminated articles | I²(%) | SMD | 95%CI | P-valued |
| Deng 2008 | 83 | -0.54 | [-1.25.0.17] | 0.14 |
| Gu 2018 | 28 | -0.23 | [-0.51,0.06] | 0.12 |
| Liu 2022 | 83 | -0.46 | [-1.19,0.27] | 0.22 |
| Zhao 2023 | 67 | -0.65 | [-1.18,-0.13] | 0.02 |
