# Supplementary material for: Effect of acupuncture on menopausal depressive disorder and serum hormone levels: a systematic review and meta-analysis
Source: Front Psychiatry. 2025 Jul 14;16:1591389. doi: 10.3389/fpsyt.2025.1591389 (PMC12301320; doi:10.3389/fpsyt.2025.1591389)
Supplement: Supplementary 1 — Clinical effectiveness rate. [file SupplementaryFile1.zip › Supplementary table 2.DOCX]

| Table2：Characteristics of Included Studies | | | | | | | | | | | | | | |
| --- | --- | --- | --- | --- | --- | --- | --- | --- | --- | --- | --- | --- | --- | --- |
| study | time | Diagnostic Criteria | experimental group | | | | control group | | | | event | period of time | Follow-up | AEs |
|  |  |  | sample size | age | interventions | Time, Frequency | sample size | age | interventions | Time, Frequency |  |  |  |  |
| Qian 2007 | 2007 | CCDM-Ⅲ | 33 | 45-60 | MA Feishu (BL13), Xinshu (BL15), Ganshu (BL18), Pishu (BL20), Shenshu (BL23), and Geshu (BL17). | 25min/d，5d/w | 30 | 46-60 | Fluoxetine | 20mg/d | HAMD-17 TESS | 6w | NA | 11 |
| Deng 2008 | 2008 | ICD-10 | 30 | 50.03±4.43 | MA(Abdominal Acupuncture) Zhongwan (RN12), Xiawan (RN10), Qihai (RN6), Guanyuan (RN4), Zhongji (RN3), Xiafengshidian (EX-LE5, bilateral), Shangqu (KI17, left), and Qipang (EX-CA4, left) | 20–30 min Daily×3d → q3d | 30 | 48.70 ±4.93 | Flupentixol Melitracen Tablets | 20mg/d | HAMD-17 KI 5-HT | 4w | 1m | NA |
| Wang 2010 | 2010 | CCMD-3 | 30 | 49.60±4.3 | MA (Abdominal Acupuncture) Zhongwan (RN12), Xiawan (RN10), Qihai (RN6), Guanyuan (RN4), Shangqu (KI17, left), Zhongji (RN3) | 20–30 min Daily×3d → q3d | 30 | 48.3 ±4.7 | Flupentixol Melitracen Tablets | 1 tablet/day | HAMD-17 | 4w | 4w | NA |
| Zhang 2010 | 2010 | CCMD-3 | 52 | 48.48±5.39 | EA 6V,8-9mA Group A: Baihui (DU20), Neiguan (PC6), Taichong (LR3), Taixi (KI3), and Sanyinjiao (SP6); Group B: Feishu (BL13), Xinshu (BL15), Ganshu (BL18), Pishu (BL20), and Shenshu (BL23). | Each group was applied on alternate days 30min/d，5d/w | 52 | 48.48±5.39 | Nilestriol Fluoxetine Hydrochloride Capsules | Nilestriol (2mg/tablet): 1 tablet orally every 2 weeks;Fluoxetine Hydrochloride Capsules (20mg/capsule): 1 capsule orally once daily in the morning | HAMD-24 KMI FSH,E2,LH | 12w | NA | NA |
| Chen 2010 | 2010 | DSM－IV | 30 | 48.1 ±4.8 | MA+CHM MA:Shenshu(BL23),Ganshu(BL18),Xinshu (BL15),Zusanli(ST36),Sanyinjiao(SP6), Shenting(DU24),Benshen(GB13),Sishencong(EX-HN1),Neiguan(PC6) CHM:Zishen Shugan Ningxin Formula | MA:30min/d CHM:once daily | 30 | 48.1 ±4.8 | Zishen Shugan Ningxin Formula | once daily | HAMD-24 FSH,E2 5-HIAA NE DA | 8w | NA | NA |
| Li 2015 | 2015 | STRAW-10 ICD-10 | 30 | 49.80+3.39 | EA dense-spare waves,10/50Hz,0.5-1.0mA Guanyuan (RN4),Zigong (EX-CA1)Tianshu(ST25),Sanyinjiao (SP6),Hegu(LI4),Taichong(LR3),Baihui(DU20),and Yintang (EX-HN3) | 30min/d,3d/w | 30 | 49.90+2.98 | escitalopram | 10mg/d | HAM-D17 MENQ0L E2,FSH,LH AST/ALT/TBIL BUN/Cr | 12w | 12w | 27 |
| Wang 2015 | 2015 | CCMD-3 | 35 | 48.72 ± 4.21 | MA Lieque (LU7, left) and Zhaohai (KI6, right); Neiguan (PC6, right) and Gongsun (SP4, left) | 30min/d 3d/w | 35 | 48.64 ± 4.82 | Wuling Capsules | a dosage of 3 capsules per administration Three times daily | SDS MENQOL | 8w | 12w | NA |
| Li  2018 | 2018 | DSM-5 ICD-10 | 116 | 49.83±3.1 | EA dilatational wave,50 HZ,0.5-1mA. Guanyuan(RN4), Zigong(EX-CA1, bilateral), Tianshu(ST25, bilateral), Sanyingjiao (SP6, bilateral), Hegu (LI4,bilateral), Taichong (LR3, bilateral), Baihui (DU20), and Yintang (EX-HN3). | 30min/d,3d/w | 106 | 49.93±3.1 | escitalopram | 10mg/d | HAMD-17 MENQOL FSH,LH,,E2 | 12w | 12w | 32 |
| Gu 2020 | 2020 | DSM-5 ICD-10 | 30 | 49±3 | MA+CHM Acupoints: Shuigou (GV26), Shaoshang (LU11), Yinbai (SP1), Daling (PC7), Shenmai (BL62), Jiache (ST6), Chengjiang (CV24), Laogong (PC8), Shangxing (GV23), Quchi (LI11) CHM: kaixin powder | MA：3d/w CHM：1 dose/d, divided bid | 28 | 50±3 | kaixin powder | 1 dose/day, divided bid (morning & evening) | HAMD-24 SDS KI | 12w | 1m | NA |
| Zhou 2022 | 2022 | STRAW-10 DSM-5 | 108 | 45-55 | EA dense-spare waves,50Hz,0.5-10mA Baihui(DU20), Yinngtang(EX-NH3), Guanyuan(RN4), Zigong(EX-CA1,bilateral), Tianshu(ST25,bilateral), Hegu(L14,bilateral), Taichong(LR3,bilateral), and Sanyinjiao(SP6,bilateral). | 30min, 3d/w | 104 | 45-55 | escitalopram | 10mg/d or 5mg/d | HAMD-17 MENQOL FSH,LH,E2 | 12w | 13-24w | NA |
| Liu 2022 | 2022 | CCMD-3 | 32 | 49.88±3.56 | MA+CHM MA:Baihui (DU20), Huangshu (KI16, bilateral), Danzhong (RN17), Sanyinjiao (SP6, bilateral) CHM:Buyang Huanwu Decoction | MA:20 min/d,6 consecutive days→1-day break  CHM:1 dose/day, divided bid (morning & evening), taken warm,Administered for 1 month → 2-day break | 32 | 48.97±2.68 | Buyang Huanwu Decoction | 1 dose/day, divided bid (morning & evening), taken warm Cycle: Administered for 1 month → 2-day break | HAMD-17 KI hs-CRP TCM syndrome score clinical efficacy | 2m | NA | NA |
| Zhao 2023 | 2023 | STRAW ICD-10 ICSD-3 | 35 | 48.94 ± 2.25 | MA Yintang (EX-HN3), Baihui (GV20), Guanyuan(CV4), Yinjiao (CV7) and bilateral Neiguan (PC6), Taixi (KI3),Taichong (LR3), Sanyinjiao (SP6), and Zigong (EX-CA1). | 30min,three sessions per week forthe first 3 weeks,two sessions per week for the next 3 weeks,and one session per week for the final 2 weeks | 35 | 48.80 ± 2.07 | SA Zhouliao(LI12),Shouwuli(LI13),Tiaokou(ST38), Yangfu(GB38), Xuanzhong(GB39), Sanyangluo(TE8), and Sidu(TE9). | 30min,three sessions per week forthe first 3 weeks,two sessions per week for the next 3 weeks,and one session per week for the final 2 weeks | HAM-D17 PSQI FSH,LH,,E2 KI | 8w | 16w | NA |
| Wang 2023 | 2023 | DSM-IV | 31 | 49.71 ± 4.29 | MA(Abdominal Acupuncture)+CHM MA:Zhongwan(RN12), Xiawan(RN10),Qihai(RN6), Guanyuan(RN4), Shangqu(KL17), Shangfengshi Point(extra-point) and Qipang (extra-point) CHM:BushenTiaogan (BSTG) formula | MA:20 min,Daily×3d→q3d CHM:two packs daily | 32 | 51.16 ± 3.99 | CHM+SA | SA:20 min,Daily×3d→q3d CHM:two packs daily | GCS SDS SAD | 8W | 12W | 5 |
| HAMD:the Hamilton Depression Rating Scale;MENQOL:the menopause-specific quality of life scale;SDS:self-rating depression scale;KI:Kupperman Index;PSQI:Pittsburgh Sleep Quality Index;FSH:Follicle-stimulating hormone;LH:luteinizing hormone;E2:estrogen;STRAW:Stages of Reproductive Aging Workshop;ICD-10:The International Classification of Diseases-Ten Edition;ICSD-3:International Classification of Sleep Disorders Third Edition;CHM:Chinese herbal medicine;MA:Manual Acupuncture;EA:Electroacupuncture;SA:Sham Acupuncture;NA:Not Applicable | | | | | | | | | | | | | | |
|  |  |  |  |  |  |  |  |  |  |  |  |  |  |  |
