# Supplementary material for: Effect of acupuncture on menopausal depressive disorder and serum hormone levels: a systematic review and meta-analysis
Source: Front Psychiatry. 2025 Jul 14;16:1591389. doi: 10.3389/fpsyt.2025.1591389 (PMC12301320; doi:10.3389/fpsyt.2025.1591389)
Supplement: Supplementary 1 — Clinical effectiveness rate. [file SupplementaryFile1.zip › Supplementary table 1.DOCX]

| Table 1：Quality assessment of included studies | | | | | |
| --- | --- | --- | --- | --- | --- |
| **Authors** | **Year** | **Randomization** | **Blinding** | **Withdrawals and dropouts** | **Jadad score** |
| Zhou SH,2004 | 2004 | 1 | 0 | 1 | 2 |
| Guo YM,2005 | 2005 | 1 | 0 | 1 | 2 |
| Ding L,2007 | 2007 | 1 | 0 | 0 | 1 |
| Qian J,2007 | 2007 | 2 | 0 | 1 | 3 |
| Deng AJ,2008 | 2008 | 2 | 0 | 1 | 3 |
| Qiang BQ,2008 | 2008 | 2 | 0 | 0 | 2 |
| Ma J,2009 | 2009 | 1 | 0 | 0 | 1 |
| Wang XY,2010 | 2010 | 2 | 0 | 1 | 3 |
| Zhang YL,2010 | 2010 | 2 | 0 | 1 | 3 |
| Zheng SH,2010 | 2010 | 1 | 0 | 0 | 1 |
| Chen Z,2010 | 2010 | 1 | 0 | 0 | 1 |
| Chi H,2011 | 2011 | 1 | 0 | 0 | 1 |
| Xing K,2011 | 2011 | 2 | 0 | 0 | 2 |
| Ma YB,2011 | 2013 | 1 | 0 | 1 | 2 |
| Zhang YQ,2013 | 2013 | 1 | 0 | 0 | 1 |
| Dong Y,2015 | 2015 | 1 | 0 | 1 | 2 |
| Li HB,2015 | 2015 | 1 | 0 | 1 | 2 |
| Li ZF,2015 | 2015 | 2 | 2 | 1 | 5 |
| Ning Y,2015 | 2015 | 2 | 0 | 0 | 2 |
| Sun YJ,2015 | 2015 | 1 | 0 | 0 | 1 |
| Wang C,2015 | 2015 | 2 | 0 | 1 | 3 |
| Zhang J,2015 | 2015 | 0 | 0 | 0 | 0 |
| Niu XS,2017 | 2017 | 2 | 0 | 0 | 2 |
| Li S,2018 | 2018 | 2 | 2 | 1 | 5 |
| Shi J,2018 | 2018 | 1 | 0 | 0 | 1 |
| Liu HF,2019 | 2019 | 2 | 0 | 0 | 2 |
| Li P,2020 | 2020 | 2 | 0 | 0 | 2 |
| Dai W,2022 | 2022 | 2 | 0 | 0 | 2 |
| Men SJ,2022 | 2022 | 2 | 0 | 0 | 2 |
| Zhou JH,2022 | 2022 | 2 | 0 | 1 | 3 |
| Zhao FY,2023 | 2023 | 2 | 2 | 1 | 5 |
| Liang ZQ,2024 | 2024 | 2 | 0 | 0 | 2 |
| Shi XL,2010 | 2010 | 1 | 0 | 0 | 1 |
| Chen GZ,2010 | 2010 | 2 | 0 | 1 | 3 |
| Shi XL,2011 | 2011 | 1 | 0 | 0 | 1 |
| Li N,2012 | 2012 | 1 | 0 | 1 | 2 |
| Xie YQ,2013 | 2013 | 1 | 0 | 0 | 1 |
| Huang HL,2016 | 2016 | 1 | 0 | 0 | 1 |
| Huang HL,2017 | 2017 | 2 | 0 | 0 | 2 |
| Sui L,2019 | 2019 | 1 | 0 | 1 | 2 |
| Tang NL,2019 | 2019 | 1 | 0 | 0 | 1 |
| Che JX,2020 | 2020 | 1 | 0 | 0 | 1 |
| Gu T,2020 | 2020 | 2 | 0 | 1 | 3 |
| Pan L,2021 | 2021 | 1 | 0 | 0 | 1 |
| Wu Y,2022 | 2022 | 2 | 0 | 0 | 2 |
| Liu XY,2022 | 2022 | 2 | 0 | 1 | 3 |
| Wang J,2023 | 2023 | 2 | 1 | 1 | 4 |

Jadad Scale (Oxford Quality Score):
This tool assesses methodological quality of randomized controlled trials (RCTs) across three domains (total score: 0–5):

Randomization:1 point for mentioning "randomization," +1 for valid methods (e.g., random number table).

Double-blinding:1 point for mentioning "blinding," +1 for detailed procedures (e.g., placebo).

Withdrawals:1 point for reporting dropouts and appropriate handling (e.g., ITT analysis).
Scores classify studies as high- (>3), moderate- (2), or low-quality (0–1).
