# Supplementary material for: Effect of acupuncture on menopausal depressive disorder and serum hormone levels: a systematic review and meta-analysis
Source: Front Psychiatry. 2025 Jul 14;16:1591389. doi: 10.3389/fpsyt.2025.1591389 (PMC12301320; doi:10.3389/fpsyt.2025.1591389)
Supplement: Supplementary 1 — Clinical effectiveness rate. [file SupplementaryFile1.zip › Supplementary material 9.PDF]

**EMbase**

'acupuncture therapy' OR 'shonishin' OR 'acupuncture' OR 'acupuncture, electric' OR 'electric acupuncture' OR 'electrical acupoint stimulation' OR 'electrical acupuncture' OR 'electro-acupuncture' OR 'electrode acupuncture' OR 'electronic acupuncture' OR 'electroacupuncture' OR 'acupuncture, ear' OR 'acupuncture, earlobe' OR 'auriculo-acupuncture' OR 'auriculoacupuncture' OR 'auriculotherapy' OR 'ear acupuncture' OR 'earlobe acupuncture' OR 'auricular acupuncture'

AND

'menopausal age'/exp OR 'menopausal age' OR 'menopausal female'/exp OR 'menopausal female' OR 'menopausal woman'/exp OR 'menopausal woman' OR 'menopause age'/exp OR 'menopause age' OR 'menopauze'/exp OR 'menopauze' OR 'menopause'/exp OR 'menopause' OR 'climacteric':ab,ti OR 'menopausal transition':ab,ti OR 'perimenopausal female':ab,ti OR 'perimenopausal woman':ab,ti OR 'perimenopause':ab,ti OR 'climacterium':ab,ti

AND

'central depression'/exp OR 'central depression' OR (('central'/exp OR central) AND ('depression'/exp OR depression)) OR 'clinical depression'/exp OR 'clinical depression' OR 'depressive disease'/exp OR 'depressive disease' OR 'depressive disorder'/exp OR 'depressive disorder' OR 'depressive episode'/exp OR 'depressive episode' OR 'depressive illness'/exp OR 'depressive illness' OR 'depressive personality disorder'/exp OR 'depressive personality disorder' OR 'depressive state'/exp OR 'depressive state' OR 'depressive symptom'/exp OR 'depressive symptom' OR 'depressive syndrome'/exp OR 'depressive syndrome' OR 'depressivity'/exp OR 'depressivity' OR 'mental depression'/exp OR 'mental depression' OR 'parental depression'/exp OR 'parental depression' OR 'depression'/exp OR 'depression'

**Cochrace**

ID Search Hits

#1 (Treatment, Acupuncture):ti,ab,kw OR (Therapy, Acupuncture):ti,ab,kw OR (Acupuncture Treatment):ti,ab,kw OR (Acupuncture Treatments):ti,ab,kw OR (Acupuncture Anesthesia):ti,ab,kw 17455

#2 (Anesthesia, Acupuncture):ti,ab,kw OR (Analgesia, Acupuncture):ti,ab,kw 2029

#3 acupuncture 23295

#4 electroacupuncture 4005

#5 ear Acupuncture 1068

#6 (Acupunctures, Auricular):ti,ab,kw OR (Acupuncture, Auricular):ti,ab,kw OR (Auricular Acupuncture):ti,ab,kw OR (Auricular Acupunctures):ti,ab,kw OR (Ear Acupuncture):ti,ab,kw 1481

#7 (Ear Acupunctures):ti,ab,kw OR (Acupunctures, Ear):ti,ab,kw 1

#8 #1 OR #2 OR #3 OR #4 OR #5 OR #6 OR #7 24226

#9 Perimenopause 440

#10 (Pre-Menopause):ti,ab,kw OR (Pre-menopausal Period):ti,ab,kw OR (Period, Pre-menopausal):ti,ab,kw OR (Period, Post-menopausal):ti,ab,kw OR (Post menopausal Period):ti,ab,kw 1296

#11 (Post-Menopause):ti,ab,kw OR (Post Menopause):ti,ab,kw OR (Post-menopausal Period):ti,ab,kw OR (Post-Menopauses):ti,ab,kw OR (Premature Menopause):ti,ab,kw 2562

#12 Climacteric 1985
#13 menopause 9338
#14 #9 OR #10 OR #11 OR #12 OR #13 11563
#15 (Emotional Depression):ti,ab,kw OR (Depressive Symptoms):ti,ab,kw OR (Depressive Symptom):ti,ab,kw OR (Symptom, Depressive):ti,ab,kw OR (Depression, Emotional):ti,ab,kw 30719
#16 Depression 113456
#17 depressive disorder 27208
#18 (Melancholia):ti,ab,kw OR (Melancholias):ti,ab,kw OR (Depressive Neurosis):ti,ab,kw OR (Neuroses, Depressive):ti,ab,kw OR (Depressive Neuroses):ti,ab,kw 482
#19 (Disorder, Depressive):ti,ab,kw OR (Depressive Disorders):ti,ab,kw OR (Disorders, Depressive):ti,ab,kw OR (Neurosis, Depressive):ti,ab,kw OR (Endogenous Depression):ti,ab,kw 30353
#20 (Endogenous Depressions):ti,ab,kw OR (Depression, Endogenous):ti,ab,kw OR (Depressions, Endogenous):ti,ab,kw OR (Depressive Syndrome):ti,ab,kw OR (Syndrome, Depressive):ti,ab,kw 4065
#21 (Syndromes, Depressive):ti,ab,kw OR (Depressive Syndromes):ti,ab,kw OR (Unipolar Depressions):ti,ab,kw OR (Unipolar Depression):ti,ab,kw OR (Depression, Unipolar):ti,ab,kw 1530
#22 (Depressions, Unipolar):ti,ab,kw OR (Depressions, Neurotic):ti,ab,kw OR (Depression, Neurotic):ti,ab,kw OR (Neurotic Depression):ti,ab,kw OR (Neurotic Depressions):ti,ab,kw 285
#23 #15 OR #16 OR #17 OR #18 OR #19 OR #20 OR #21 OR #22 119493
#24 #8 AND #14 AND #23 80

**EBSCO**

('acupuncture therapy' OR 'shonishin' OR 'acupuncture' OR 'acupuncture, electric' OR 'electric acupuncture' OR 'electrical acupoint stimulation' OR 'electrical acupuncture' OR 'electro-acupuncture' OR 'electrode acupuncture' OR 'electronic acupuncture' OR 'electroacupuncture' OR 'acupuncture, ear' OR 'acupuncture, earlobe' OR 'auriculo-acupuncture' OR 'auriculoacupuncture' OR 'auriculotherapy' OR 'ear acupuncture' OR 'earlobe acupuncture' OR 'auricular acupuncture') AND ('menopausal age'/exp OR 'menopausal age' OR 'menopausal female'/exp OR 'menopausal female' OR 'menopausal woman'/exp OR 'menopausal woman' OR 'menopause age'/exp OR 'menopause age' OR 'menopauze'/exp OR 'menopauze' OR 'menopause'/exp OR 'menopause' OR 'climacteric':ab,ti OR 'menopausal transition':ab,ti OR 'perimenopausal female':ab,ti OR 'perimenopausal woman':ab,ti OR 'perimenopause':ab,ti OR 'climacterium':ab,ti) AND ('central depression'/exp OR 'central depression' OR (('central'/exp OR central) AND ('depression'/exp OR depression)) OR 'clinical depression'/exp OR 'clinical depression' OR 'depressive disease'/exp OR 'depressive disease' OR 'depressive disorder'/exp OR 'depressive disorder' OR 'depressive episode'/exp OR 'depressive episode' OR 'depressive illness'/exp OR 'depressive illness' OR 'depressive personality disorder'/exp OR 'depressive personality disorder' OR 'depressive state'/exp OR 'depressive state' OR 'depressive symptom'/exp OR 'depressive symptom' OR 'depressive syndrome'/exp OR 'depressive syndrome' OR 'depressivity'/exp OR 'depressivity' OR 'mental depression'/exp OR 'mental depression' OR 'parental depression'/exp OR 'parental depression' OR 'depression'/exp OR 'depression')

**PubMed**
("acupunctural"[All Fields] OR "acupuncture"[MeSH Terms] OR "acupuncture"[All Fields] OR "acupuncture therapy"[MeSH Terms] OR ("acupuncture"[All Fields] AND "therapy"[All Fields]) OR "acupuncture therapy"[All Fields] OR "acupuncture s"[All Fields] OR "acupunctured"[All Fields] OR "acupunctures"[All Fields] OR "acupuncturing"[All Fields] OR ("electroacupuncture"[MeSH Terms] OR "electroacupuncture"[All Fields] OR "electroacupuncturing"[All Fields]) OR ("acupuncture, ear"[MeSH Terms] OR ("acupuncture"[All Fields] AND "ear"[All Fields]) OR "ear acupuncture"[All Fields] OR ("ear"[All Fields] AND "acupuncture"[All Fields]))) AND ("climacteric"[MeSH Terms] OR "climacteric"[All Fields] OR "climacterics"[All Fields] OR "climacterical"[All Fields] OR "menopause"[MeSH Terms] OR "menopause"[All Fields] OR ("menopause"[MeSH Terms] OR "menopause"[All Fields] OR "menopausal"[All Fields] OR "menopaused"[All Fields] OR "menopauses"[All Fields]) OR ("perimenopausal"[All Fields] OR "perimenopausally"[All Fields] OR "perimenopause"[MeSH Terms] OR "perimenopause"[All Fields])) AND ("depressive disorder"[MeSH Terms] OR ("depressive"[All Fields] AND "disorder"[All Fields]) OR "depressive disorder"[All Fields] OR ("depressed"[All Fields] OR "depression"[MeSH Terms] OR "depression"[All Fields] OR "depressions"[All Fields] OR "depression s"[All Fields] OR "depressive disorder"[MeSH Terms] OR ("depressive"[All Fields] AND "disorder"[All Fields]) OR "depressive disorder"[All Fields] OR "depressivity"[All Fields] OR "depressive"[All Fields] OR "depressively"[All Fields] OR "depressiveness"[All Fields] OR "depressives"[All Fields]))

**Web of science**
(TS=(climacteric) or TS=(climacterics) or TS=(climacterical) or TS=(menopause) or TS=(menopausal) or TS=(menopaused) or TS=(perimenopausal) or TS=(menopauses) or TS=(perimenopausTSy) or TS=(perimenopause)) and (TS=(acupunctural) or TS=(acupuncture) or TS=(acupuncture therapy) or TS=(acupunctures) or TS=(acupuncturing) or TS=(electroacupuncture) or TS=(electroacupuncturing) or TS=(ear acupuncture)) and (TS=(depressive disorder) or TS=(depressive) or TS=(depressed) or TS=(depression) or TS=(depressions) or TS=(depressivity) or TS=(depressively) or TS=(depressives) or TS=(depressiveness))

**Scopus**
climacteric OR climacterics OR climacterical OR menopause OR menopausal OR menopaused OR perimenopausal OR menopauses OR perimenopaustsy OR perimenopause
AND
acupunctural OR acupuncture OR acupuncture AND therapy OR acupunctures OR acupuncturing OR electroacupuncture OR electroacupuncturing OR ear AND acupuncture
AND
depressive AND disorder OR depressive OR depressed OR depression OR depressions OR depressivity OR depressively OR depressives OR depressiveness

**Wanfang:**
(acupuncture OR auricular acupuncture OR electroacupuncture) AND (depression OR

depressive disorder OR depressive state OR depressive symptoms OR major depressive disorder OR depressive-like behavior) AND (menopause OR perimenopause OR postmenopause)

**VIP：**

(all fields:acupuncture OR all fields:"auricular acupuncture" OR all fields:electroacupuncture) AND(all fields:depression OR all fields:"depressive disorder" OR all fields:"depressive state" OR all fields:"depressive syndrome" OR all fields:"depressive disease" OR all fields:"major depressive disorder" OR all fields:"depressive symptoms" OR all fields:"depressive-like behavior")AND(all fields:menopause OR all fields:perimenopause OR all fields:postmenopause)

**CNKI：**

(title/abstract/keywords:(acupuncture OR electroacupuncture OR auricular acupuncture)) AND(title/abstract/keywords:(depression OR depressive disorder OR depressive state OR depressive syndrome OR depressive disease OR major depressive disorder OR depressive symptoms OR depressive-like behavior))AND(title/abstract/keywords:(menopause OR perimenopause OR postmenopause))
