# Supplementary material for: Effect of acupuncture on menopausal depressive disorder and serum hormone levels: a systematic review and meta-analysis
Source: Front Psychiatry. 2025 Jul 14;16:1591389. doi: 10.3389/fpsyt.2025.1591389 (PMC12301320; doi:10.3389/fpsyt.2025.1591389)

Supplementary 8

Figure 1: The forest plot of KI

Figure 2-5: The forest plot of KI sensitivity analysis

Figure 6: The forest plot of HAMD-24 group analysis （Control Group Type）

Figure 7: The forest plot of HAMD-24 group analysis （Acupuncture sites）

1

	Experimental			Control				Std. Mean Difference
Study or Subgroup	Mean	SD	Total	Mean	SD	Total	Weight	IV, Random, 95% CI
Deng 2008	13.17	5.65	29	15.41	9.68	29	24.9%	-0.28 [-0.80, 0.24]
Gu 2018	14.28	3.21	30	18.82	4.17	28	23.8%	-1.21 [-1.77, -0.65]
Liu 2022	13.22	2.87	32	14.75	3.08	32	25.3%	-0.51 [-1.01, -0.01]
Zhao 2023	13.79	4.38	35	13.54	2.67	35	26.0%	0.07 [-0.40, 0.54]
**Total (95% CI)**			**126**			**124**	**100.0%**	**-0.47 [-0.98, 0.05]**

Heterogeneity: $Tau^2 = 0.21$; $Chi^2 = 12.11$, df = 3 (P = 0.007); $I^2 = 75\%$

Test for overall effect: Z = 1.79 (P = 0.07)

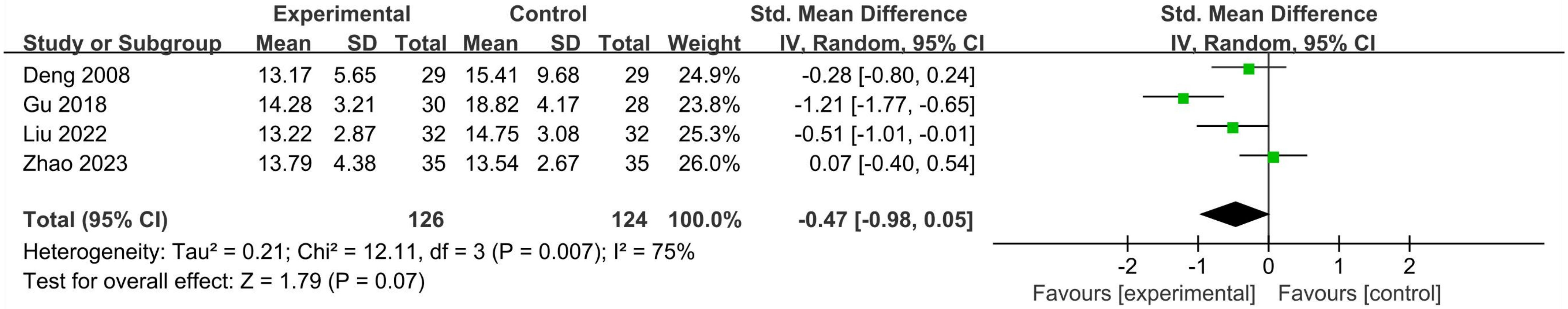

2

	Experimental			Control				Std. Mean Difference
Study or Subgroup	Mean	SD	Total	Mean	SD	Total	Weight	IV, Random, 95% CI
Deng 2008	13.17	5.65	29	15.41	9.68	29	0.0%	-0.28 [-0.80, 0.24]
Gu 2018	14.28	3.21	30	18.82	4.17	28	32.1%	-1.21 [-1.77, -0.65]
Liu 2022	13.22	2.87	32	14.75	3.08	32	33.6%	-0.51 [-1.01, -0.01]
Zhao 2023	13.79	4.38	35	13.54	2.67	35	34.3%	0.07 [-0.40, 0.54]
**Total (95% CI)**			**97**			**95**	**100.0%**	**-0.54 [-1.25, 0.17]**

Heterogeneity: $Tau^2 = 0.33$; $Chi^2 = 11.70$, df = 2 (P = 0.003); $I^2 = 83\%$

Test for overall effect: Z = 1.48 (P = 0.14)

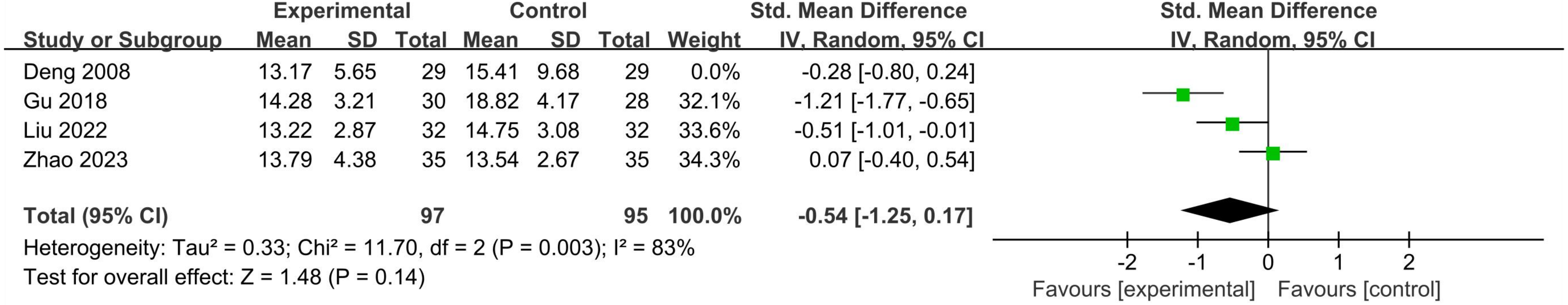

3

	Experimental			Control				Std. Mean Difference
Study or Subgroup	Mean	SD	Total	Mean	SD	Total	Weight	IV, Random, 95% CI
Deng 2008	13.17	5.65	29	15.41	9.68	29	31.2%	-0.28 [-0.80, 0.24]
Gu 2018	14.28	3.21	30	18.82	4.17	28	0.0%	-1.21 [-1.77, -0.65]
Liu 2022	13.22	2.87	32	14.75	3.08	32	32.9%	-0.51 [-1.01, -0.01]
Zhao 2023	13.79	4.38	35	13.54	2.67	35	35.9%	0.07 [-0.40, 0.54]
**Total (95% CI)**			**96**			**96**	**100.0%**	**-0.23 [-0.57, 0.11]**

Heterogeneity: Tau² = 0.02; Chi² = 2.78, df = 2 (P = 0.25); I² = 28%

Test for overall effect: Z = 1.34 (P = 0.18)

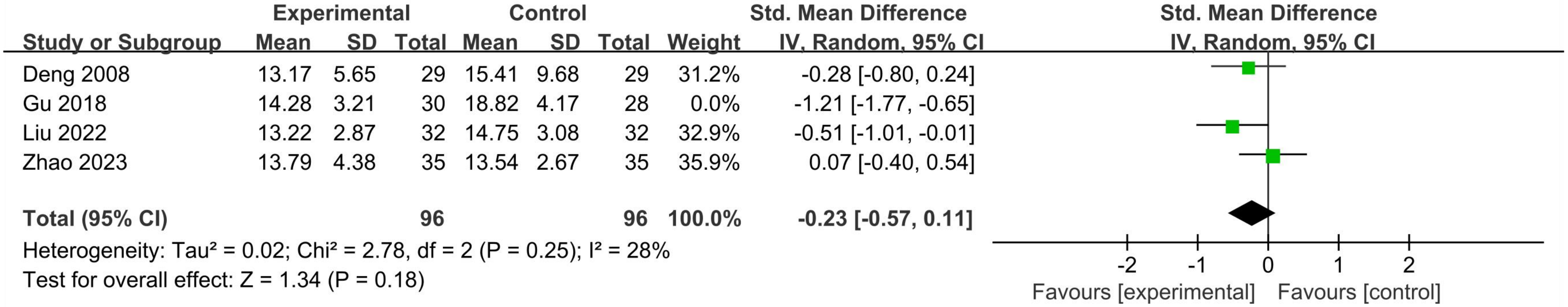

4

Study or Subgroup	Experimental Mean	Experimental SD	Experimental Total	Control Mean	Control SD	Control Total	Weight	Std. Mean Difference IV, Random, 95% CI
Deng 2008	13.17	5.65	29	15.41	9.68	29	33.3%	-0.28 [-0.80, 0.24]
Gu 2018	14.28	3.21	30	18.82	4.17	28	32.3%	-1.21 [-1.77, -0.65]
Liu 2022	13.22	2.87	32	14.75	3.08	32	0.0%	-0.51 [-1.01, -0.01]
Zhao 2023	13.79	4.38	35	13.54	2.67	35	34.4%	0.07 [-0.40, 0.54]
**Total (95% CI)**			**94**			**92**	**100.0%**	**-0.46 [-1.19, 0.27]**

Heterogeneity: $Tau^2 = 0.34$; $Chi^2 = 11.97$, $df = 2$ ($P = 0.003$); $I^2 = 83\%$

Test for overall effect: $Z = 1.24$ ($P = 0.22$)

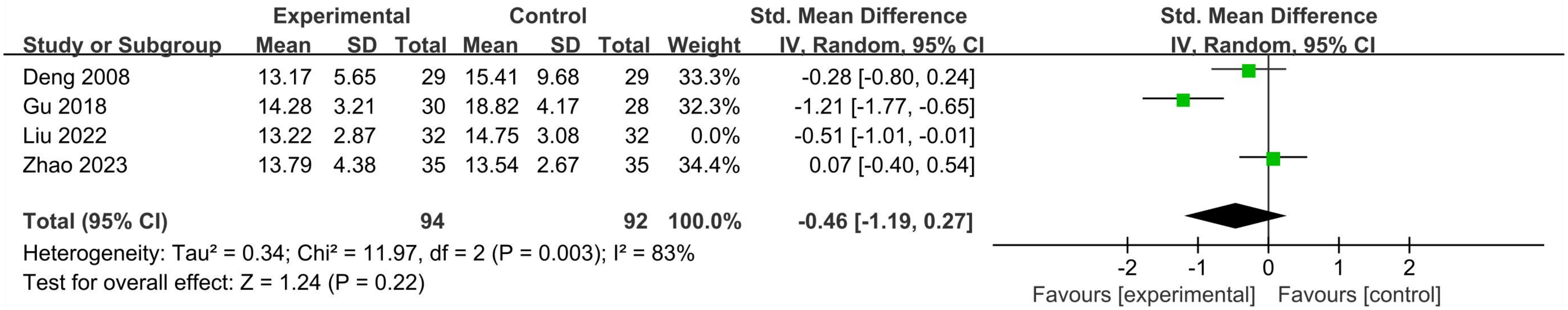

5

	Experimental			Control				Std. Mean Difference
Study or Subgroup	Mean	SD	Total	Mean	SD	Total	Weight	IV, Random, 95% CI
Deng 2008	13.17	5.65	29	15.41	9.68	29	33.7%	-0.28 [-0.80, 0.24]
Gu 2018	14.28	3.21	30	18.82	4.17	28	31.8%	-1.21 [-1.77, -0.65]
Liu 2022	13.22	2.87	32	14.75	3.08	32	34.5%	-0.51 [-1.01, -0.01]
Zhao 2023	13.79	4.38	35	13.54	2.67	35	0.0%	0.07 [-0.40, 0.54]
**Total (95% CI)**			**91**			**89**	**100.0%**	**-0.65 [-1.18, -0.13]**

Heterogeneity: $Tau^2 = 0.15$; $Chi^2 = 6.06$, df = 2 (P = 0.05); $I^2 = 67\%$

Test for overall effect: Z = 2.42 (P = 0.02)

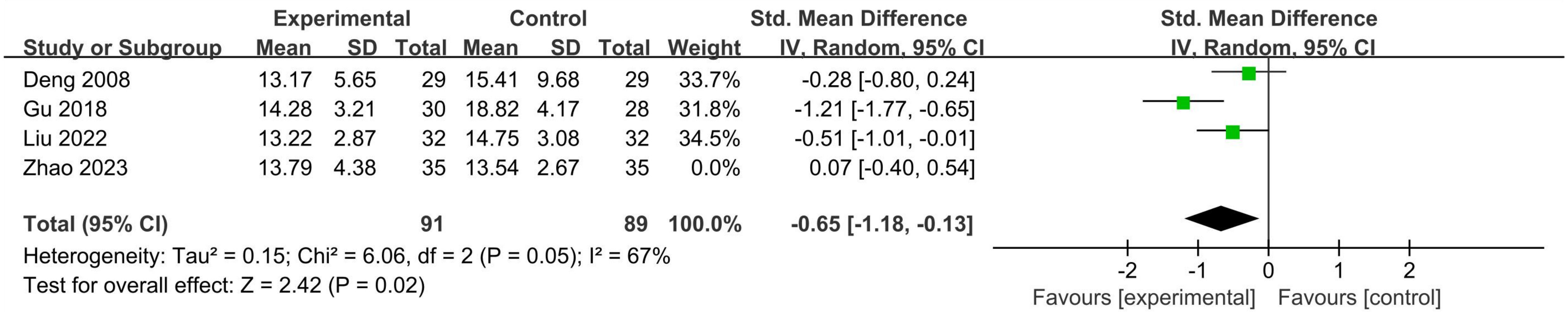

6

Study or Subgroup	Experimental Mean	Experimental SD	Experimental Total	Control Mean	Control SD	Control Total	Weight	Std. Mean Difference IV, Random, 95% CI
**6.2.1 CHM**								
Gu 2018	14.28	3.21	30	18.82	4.17	28	23.8%	-1.21 [-1.77, -0.65]
Liu 2022	13.22	2.87	32	14.75	3.08	32	25.3%	-0.51 [-1.01, -0.01]
**Subtotal (95% CI)**			**62**			**60**	**49.1%**	**-0.85 [-1.53, -0.16]**
Heterogeneity: Tau² = 0.17; Chi² = 3.34, df = 1 (P = 0.07); I² = 70%								
Test for overall effect: Z = 2.41 (P = 0.02)								
**6.2.2 other medicine**								
Deng 2008	13.17	5.65	29	15.41	9.68	29	24.9%	-0.28 [-0.80, 0.24]
**Subtotal (95% CI)**			**29**			**29**	**24.9%**	**-0.28 [-0.80, 0.24]**
Heterogeneity: Not applicable								
Test for overall effect: Z = 1.06 (P = 0.29)								
**6.2.3 sham acupuncture**								
Zhao 2023	13.79	4.38	35	13.54	2.67	35	26.0%	0.07 [-0.40, 0.54]
**Subtotal (95% CI)**			**35**			**35**	**26.0%**	**0.07 [-0.40, 0.54]**
Heterogeneity: Not applicable								
Test for overall effect: Z = 0.29 (P = 0.78)								
**Total (95% CI)**			**126**			**124**	**100.0%**	**-0.47 [-0.98, 0.05]**
Heterogeneity: Tau² = 0.21; Chi² = 12.11, df = 3 (P = 0.007); I² = 75%								
Test for overall effect: Z = 1.79 (P = 0.07)								
Test for subgroup differences: Chi² = 4.67, df = 2 (P = 0.10), I² = 57.2%								

Std. Mean Difference
IV, Random, 95% CI

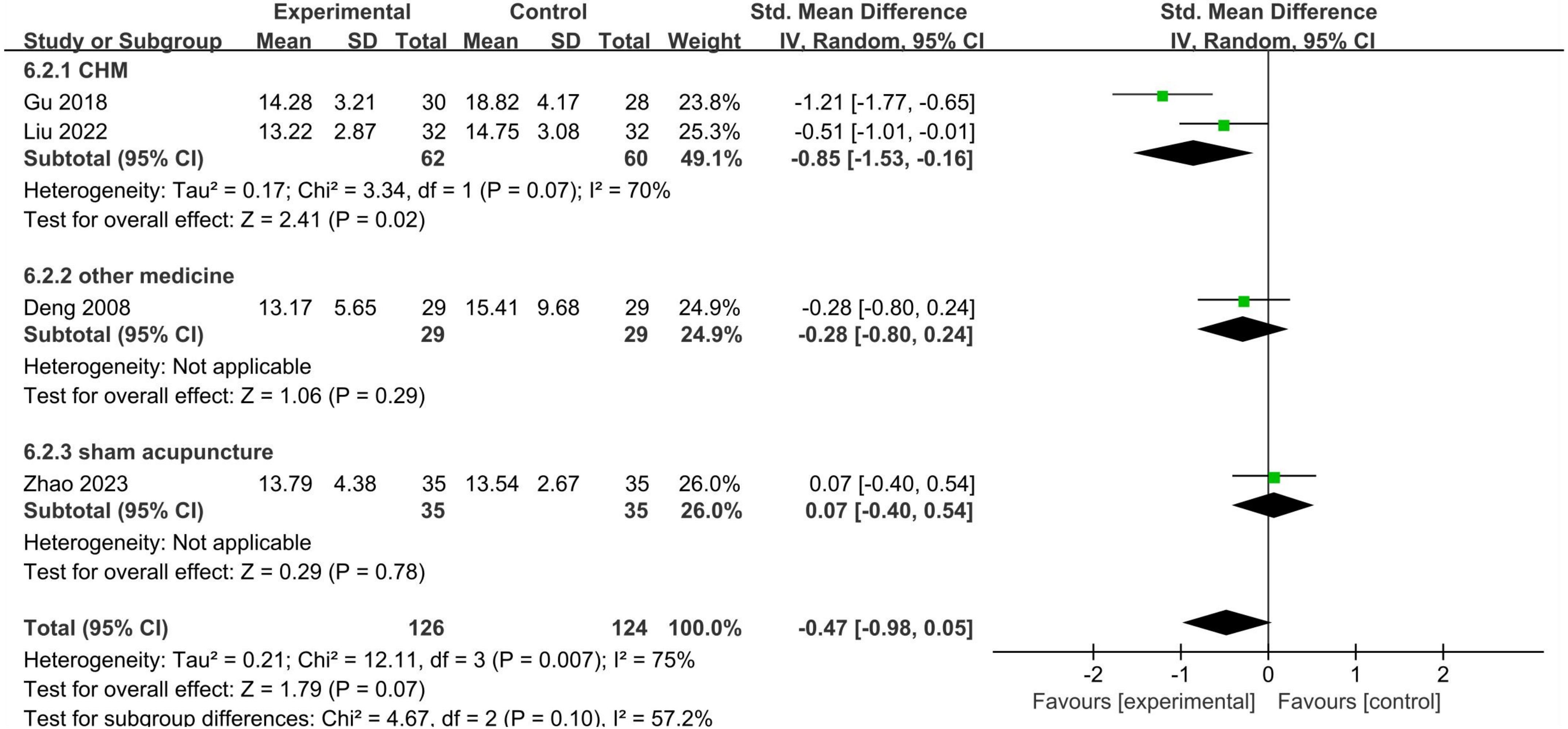

7

Study or Subgroup	Experimental Mean	Experimental SD	Experimental Total	Control Mean	Control SD	Control Total	Weight	Std. Mean Difference IV, Random, 95% CI
**6.3.1 Simple abdominal acupuncture**								
Deng 2008	13.17	5.65	29	15.41	9.68	29	24.9%	-0.28 [-0.80, 0.24]
**Subtotal (95% CI)**			**29**			**29**	**24.9%**	**-0.28 [-0.80, 0.24]**
Heterogeneity: Not applicable								
Test for overall effect: Z = 1.06 (P = 0.29)								
**6.3.2 Non-simple abdominal acupuncture**								
Gu 2018	14.28	3.21	30	18.82	4.17	28	23.8%	-1.21 [-1.77, -0.65]
Liu 2022	13.22	2.87	32	14.75	3.08	32	25.3%	-0.51 [-1.01, -0.01]
Zhao 2023	13.79	4.38	35	13.54	2.67	35	26.0%	0.07 [-0.40, 0.54]
**Subtotal (95% CI)**			**97**			**95**	**75.1%**	**-0.54 [-1.25, 0.17]**
Heterogeneity: Tau² = 0.33; Chi² = 11.70, df = 2 (P = 0.003); I² = 83%								
Test for overall effect: Z = 1.48 (P = 0.14)								
**Total (95% CI)**			**126**			**124**	**100.0%**	**-0.47 [-0.98, 0.05]**
Heterogeneity: Tau² = 0.21; Chi² = 12.11, df = 3 (P = 0.007); I² = 75%								
Test for overall effect: Z = 1.79 (P = 0.07)								
Test for subgroup differences: Chi² = 0.33, df = 1 (P = 0.57), I² = 0%								

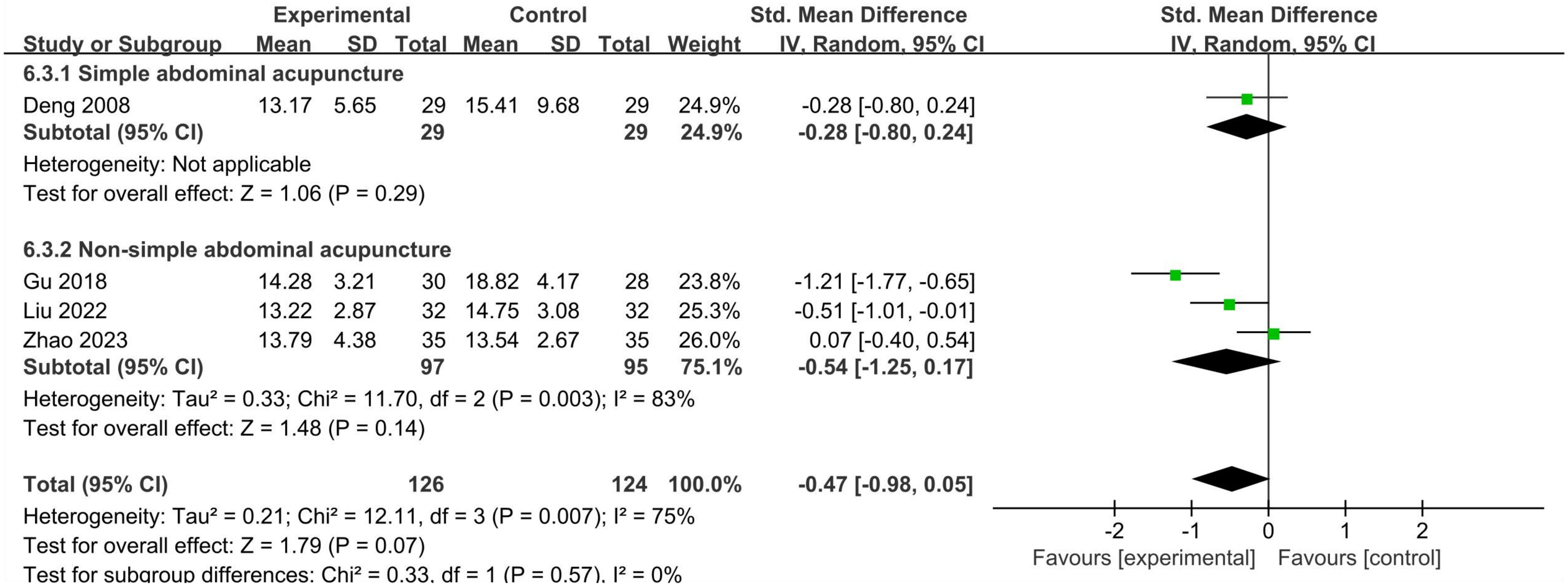

Supplement: Supplementary 1 — Clinical effectiveness rate. [file SupplementaryFile1.zip › Supplementary material 8.PDF]
