# Supplementary material for: Effect of acupuncture on menopausal depressive disorder and serum hormone levels: a systematic review and meta-analysis
Source: Front Psychiatry. 2025 Jul 14;16:1591389. doi: 10.3389/fpsyt.2025.1591389 (PMC12301320; doi:10.3389/fpsyt.2025.1591389)

Supplementary 7

Figure 1: The forest plot of MENQOL

Figure 2: The forest plot of MENQOLgroup analysis （The type of acupuncture in the experimental group）

Figure 3: The forest plot of MENQOL group analysis （Control Group Type）

1

Study or Subgroup	Experimental Mean	Experimental SD	Experimental Total	Control Mean	Control SD	Control Total	Weight	Std. Mean Difference IV, Fixed, 95% CI
Li 2015	3.94	0.71	30	4.37	0.85	30	10.4%	-0.54 [-1.06, -0.03]
Li 2018	34.89	28.28	116	39.55	30.17	105	39.7%	-0.16 [-0.42, 0.11]
Wang 2015	34.94	4.4	33	36.59	5	34	11.9%	-0.35 [-0.83, 0.14]
Zhou 2022	35.5	23.39	108	40.72	18.93	104	38.0%	-0.24 [-0.51, 0.03]
**Total (95% CI)**			**287**			**273**	**100.0%**	**-0.25 [-0.42, -0.09]**

Heterogeneity: $Chi^2 = 1.84$, df = 3 (P = 0.61); $I^2 = 0\%$

Test for overall effect: Z = 2.98 (P = 0.003)

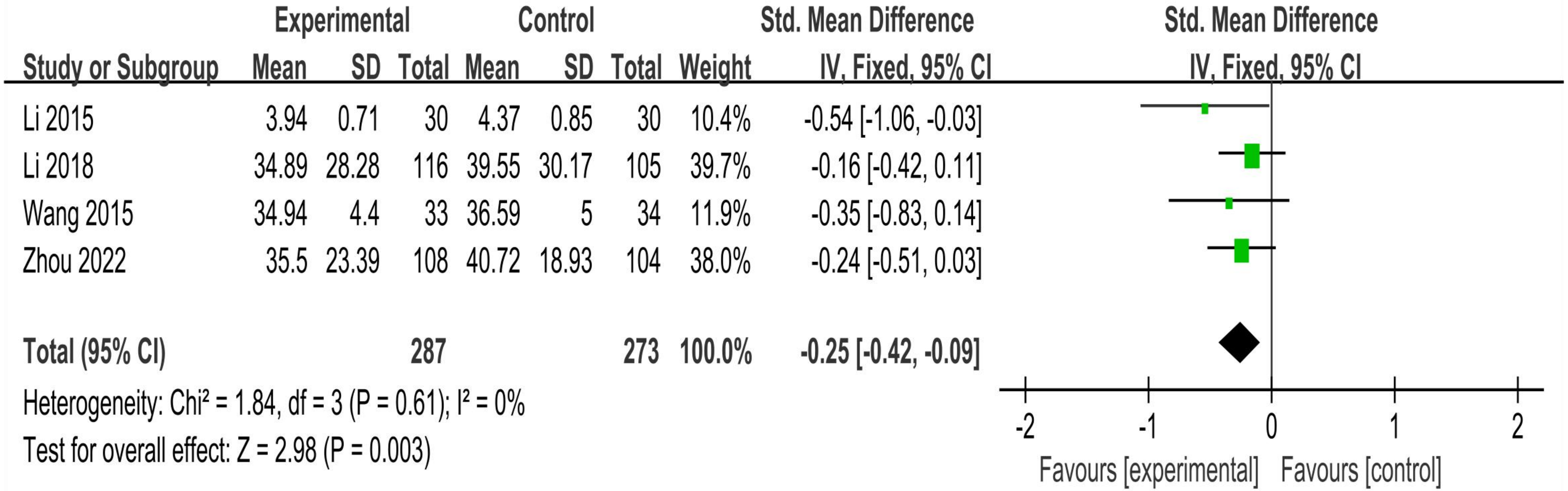

2

Study or Subgroup	Experimental Mean	Experimental SD	Experimental Total	Control Mean	Control SD	Control Total	Weight	Std. Mean Difference IV, Fixed, 95% CI
**5.2.1 EA**								
Li 2015	3.94	0.71	30	4.37	0.85	30	10.4%	-0.54 [-1.06, -0.03]
Li 2018	34.89	28.28	116	39.55	30.17	105	39.7%	-0.16 [-0.42, 0.11]
Zhou 2022	35.5	23.39	108	40.72	18.93	104	38.0%	-0.24 [-0.51, 0.03]
**Subtotal (95% CI)**			**254**			**239**	**88.1%**	**-0.24 [-0.42, -0.06]**
Heterogeneity: Chi² = 1.68, df = 2 (P = 0.43); I² = 0%								
Test for overall effect: Z = 2.66 (P = 0.008)								
**5.2.2 MA**								
Wang 2015	34.94	4.4	33	36.59	5	34	11.9%	-0.35 [-0.83, 0.14]
**Subtotal (95% CI)**			**33**			**34**	**11.9%**	**-0.35 [-0.83, 0.14]**
Heterogeneity: Not applicable								
Test for overall effect: Z = 1.40 (P = 0.16)								
**Total (95% CI)**			**287**			**273**	**100.0%**	**-0.25 [-0.42, -0.09]**
Heterogeneity: Chi² = 1.84, df = 3 (P = 0.61); I² = 0%								
Test for overall effect: Z = 2.98 (P = 0.003)								
Test for subgroup differences: Chi² = 0.16, df = 1 (P = 0.69), I² = 0%								

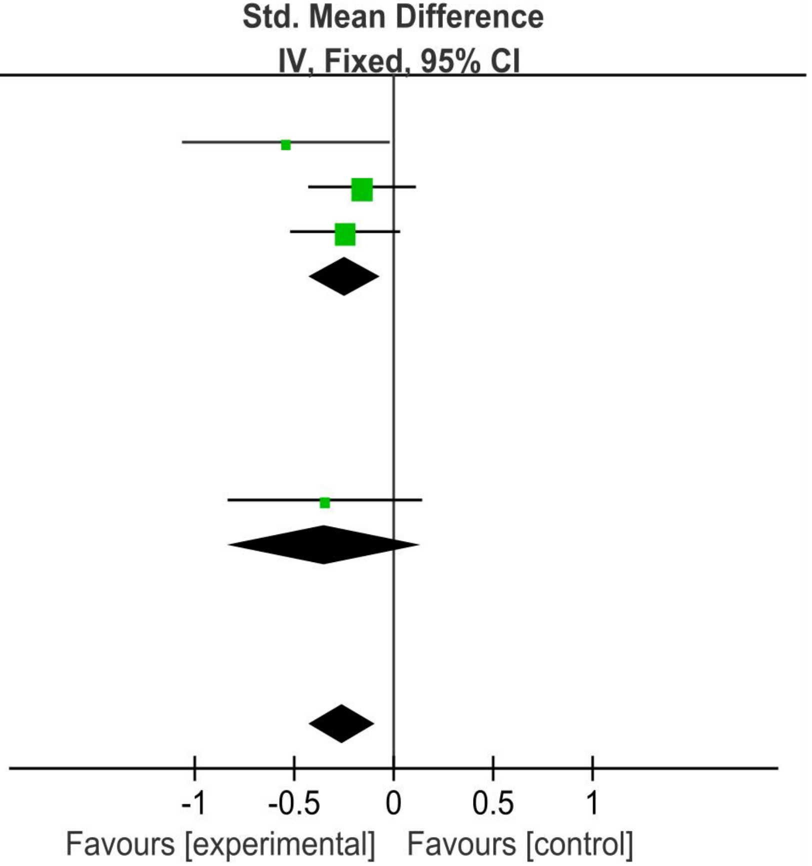

3

Study or Subgroup	Experimental Mean	Experimental SD	Experimental Total	Control Mean	Control SD	Control Total	Weight	Std. Mean Difference IV, Fixed, 95% CI
**5.2.1 other medications**								
Li 2015	3.94	0.71	30	4.37	0.85	30	10.4%	-0.54 [-1.06, -0.03]
Li 2018	34.89	28.28	116	39.55	30.17	105	39.7%	-0.16 [-0.42, 0.11]
Zhou 2022	35.5	23.39	108	40.72	18.93	104	38.0%	-0.24 [-0.51, 0.03]
**Subtotal (95% CI)**			**254**			**239**	**88.1%**	**-0.24 [-0.42, -0.06]**
Heterogeneity: Chi² = 1.68, df = 2 (P = 0.43); I² = 0%								
Test for overall effect: Z = 2.66 (P = 0.008)								
**5.2.2 CHM**								
Wang 2015	34.94	4.4	33	36.59	5	34	11.9%	-0.35 [-0.83, 0.14]
**Subtotal (95% CI)**			**33**			**34**	**11.9%**	**-0.35 [-0.83, 0.14]**
Heterogeneity: Not applicable								
Test for overall effect: Z = 1.40 (P = 0.16)								
**Total (95% CI)**			**287**			**273**	**100.0%**	**-0.25 [-0.42, -0.09]**
Heterogeneity: Chi² = 1.84, df = 3 (P = 0.61); I² = 0%								
Test for overall effect: Z = 2.98 (P = 0.003)								
Test for subgroup differences: Chi² = 0.16, df = 1 (P = 0.69), I² = 0%								

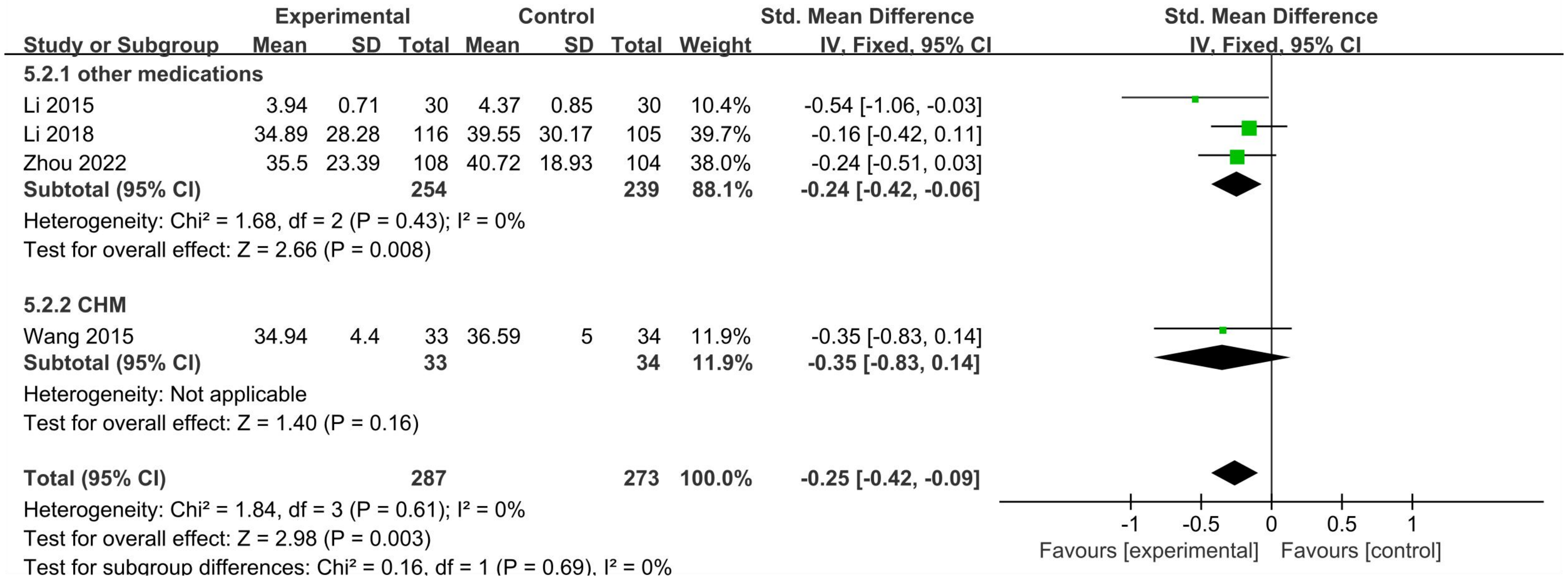

Supplement: Supplementary 1 — Clinical effectiveness rate. [file SupplementaryFile1.zip › Supplementary material 7.PDF]
