# Supplementary material for: Effect of acupuncture on menopausal depressive disorder and serum hormone levels: a systematic review and meta-analysis
Source: Front Psychiatry. 2025 Jul 14;16:1591389. doi: 10.3389/fpsyt.2025.1591389 (PMC12301320; doi:10.3389/fpsyt.2025.1591389)

Supplementary 6

Figure 1: The forest plot of Adverse reactions

Figure 2: The forest plot of Adverse reactions sensitivity analysis

Figure 3: The forest plot of Adverse reactions group analysis（The type of acupuncture in the experimental group）

Figure 4: The forest plot of Adverse reactions group analysis（Control Group Type）

Figure 5: The forest plot of Adverse reactions group analysis（Acupuncture sites）

1

	Experimental		Control			Odds Ratio
**Study or Subgroup**	**Events**	**Total**	**Events**	**Total**	**Weight**	**M-H, Random, 95% CI**
Li 2015	2	30	25	30	24.5%	0.01 [0.00, 0.08]
Li 2018	14	117	18	111	29.7%	0.70 [0.33, 1.49]
Qian 2007	2	33	9	30	25.1%	0.15 [0.03, 0.77]
Wang 2023	1	31	3	32	20.8%	0.32 [0.03, 3.28]
**Total (95% CI)**		**211**		**203**	**100.0%**	**0.16 [0.03, 0.98]**
Total events	19		55			

Heterogeneity: $Tau^2 = 2.78$; $Chi^2 = 17.81$, df = 3 (P = 0.0005); $I^2 = 83\%$

Test for overall effect: Z = 1.99 (P = 0.05)

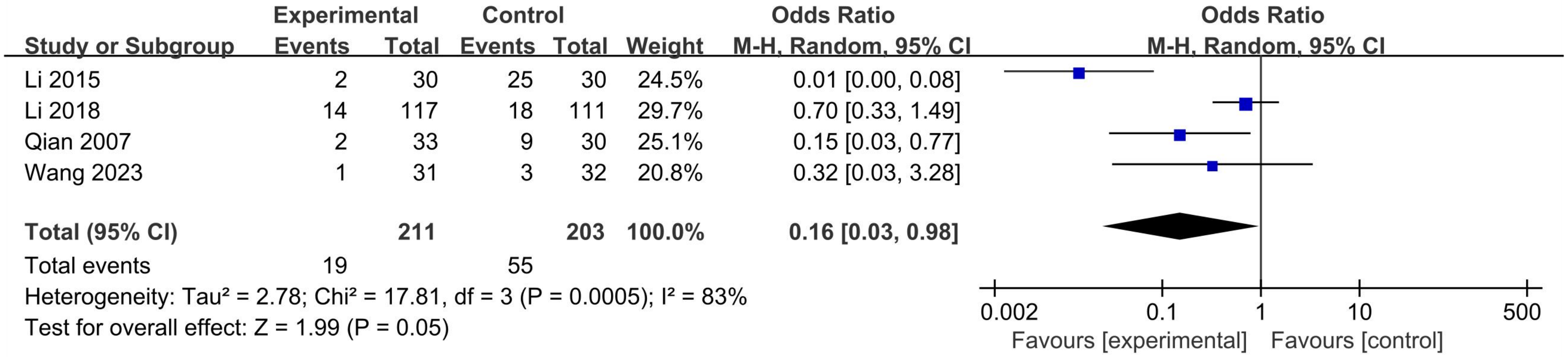

2

	Experimental		Control			Odds Ratio	Odds Ratio
**Study or Subgroup**	**Events**	**Total**	**Events**	**Total**	**Weight**	**M-H, Fixed, 95% CI**	**M-H, Fixed, 95% CI**
Li 2015	2	30	25	30	0.0%	0.01 [0.00, 0.08]	
Li 2018	14	117	18	111	58.1%	0.70 [0.33, 1.49]	
Qian 2007	2	33	9	30	31.7%	0.15 [0.03, 0.77]	
Wang 2023	1	31	3	32	10.2%	0.32 [0.03, 3.28]	
**Total (95% CI)**		**181**		**173**	**100.0%**	**0.49 [0.26, 0.92]**	
Total events	17		30				

Heterogeneity: Chi² = 3.02, df = 2 (P = 0.22); I² = 34%
Test for overall effect: Z = 2.21 (P = 0.03)

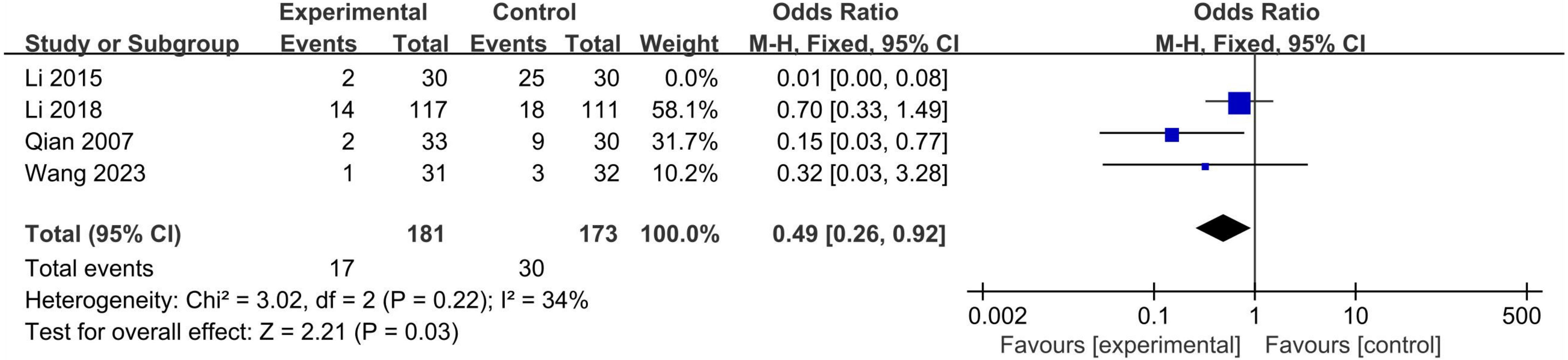

3

Study or Subgroup	Experimental Events	Experimental Total	Control Events	Control Total	Weight	Odds Ratio M-H, Random, 95% CI
**4.2.1 EA**						
Li 2015	2	30	25	30	24.5%	0.01 [0.00, 0.08]
Li 2018	14	117	18	111	29.7%	0.70 [0.33, 1.49]
**Subtotal (95% CI)**		**147**		**141**	**54.2%**	**0.11 [0.00, 5.18]**
Total events	16		43			
Heterogeneity: Tau² = 7.34; Chi² = 16.90, df = 1 (P < 0.0001); I² = 94%						
Test for overall effect: Z = 1.13 (P = 0.26)						
**4.2.2 MA**						
Qian 2007	2	33	9	30	25.1%	0.15 [0.03, 0.77]
Wang 2023	1	31	3	32	20.8%	0.32 [0.03, 3.28]
**Subtotal (95% CI)**		**64**		**62**	**45.8%**	**0.19 [0.05, 0.73]**
Total events	3		12			
Heterogeneity: Tau² = 0.00; Chi² = 0.28, df = 1 (P = 0.60); I² = 0%						
Test for overall effect: Z = 2.41 (P = 0.02)						
**Total (95% CI)**		**211**		**203**	**100.0%**	**0.16 [0.03, 0.98]**
Total events	19		55			
Heterogeneity: Tau² = 2.78; Chi² = 17.81, df = 3 (P = 0.0005); I² = 83%						
Test for overall effect: Z = 1.99 (P = 0.05)						
Test for subgroup differences: Chi² = 0.08, df = 1 (P = 0.78), I² = 0%						

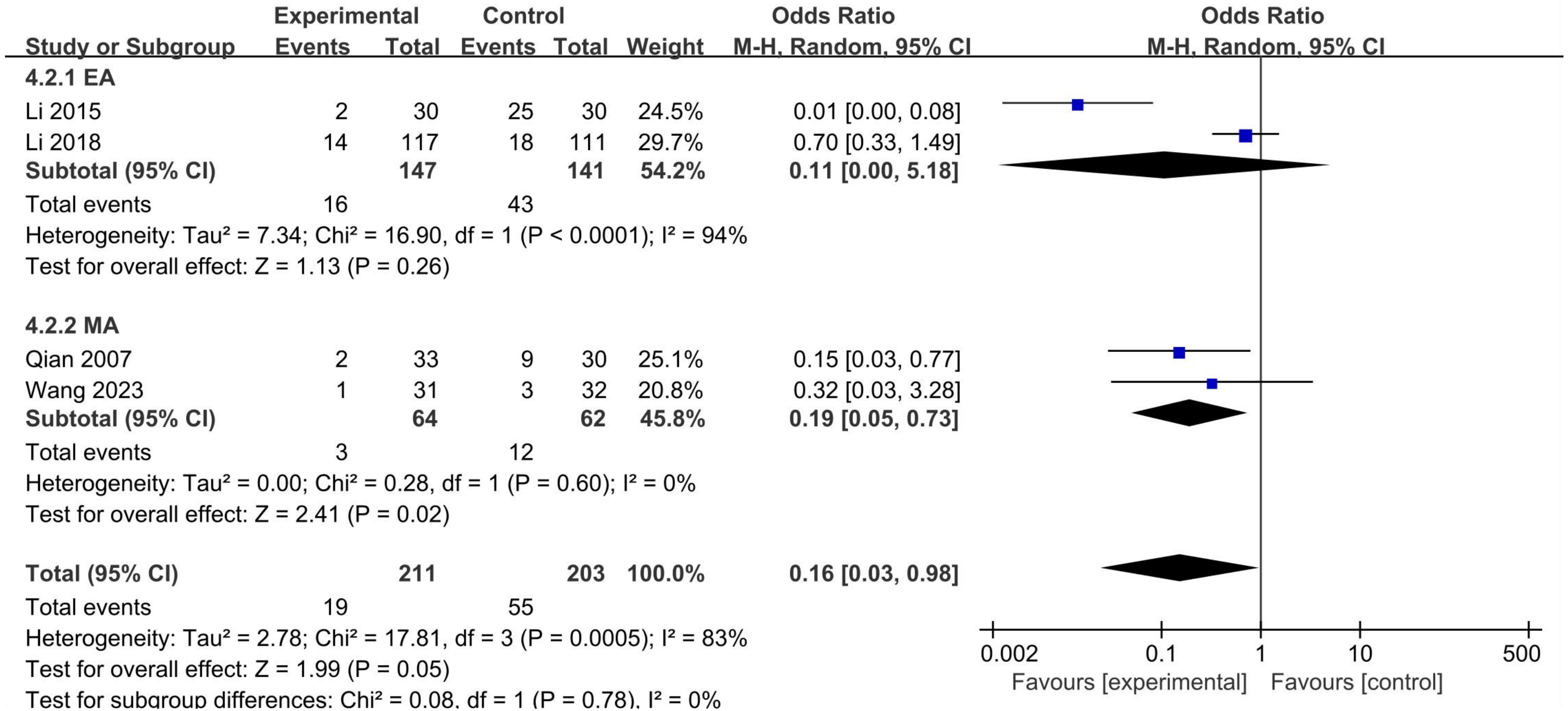

4

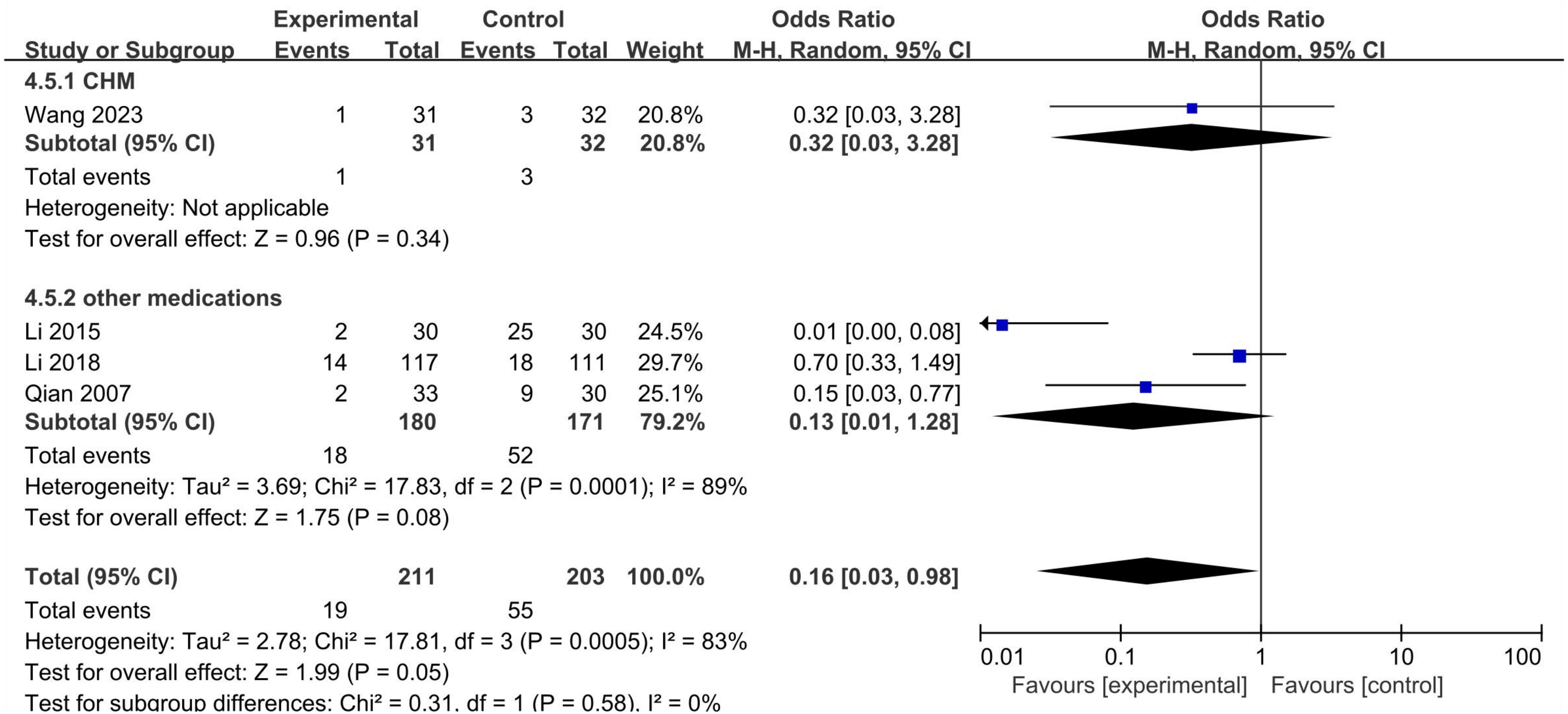
Experimental
Control
Odds Ratio
Odds Ratio
Study or Subgroup
Events
Total
Events
Total
Weight
M-H, Random, 95% CI
M-H, Random, 95% CI
4.5.1 CHM
Wang 2023 1 31 3 32 20.8% 0.32 [0.03, 3.28]
Subtotal (95% CI) 31 32 20.8% 0.32 [0.03, 3.28]
Total events 1 3
Heterogeneity: Not applicable
Test for overall effect: Z = 0.96 (P = 0.34)
4.5.2 other medications
Li 2015 2 30 25 30 24.5% 0.01 [0.00, 0.08]
Li 2018 14 117 18 111 29.7% 0.70 [0.33, 1.49]
Qian 2007 2 33 9 30 25.1% 0.15 [0.03, 0.77]
Subtotal (95% CI) 180 171 79.2% 0.13 [0.01, 1.28]
Total events 18 52
Heterogeneity: Tau² = 3.69; Chi² = 17.83, df = 2 (P = 0.0001); I² = 89%
Test for overall effect: Z = 1.75 (P = 0.08)
Total (95% CI) 211 203 100.0% 0.16 [0.03, 0.98]
Total events 19 55
Heterogeneity: Tau² = 2.78; Chi² = 17.81, df = 3 (P = 0.0005); I² = 83%
Test for overall effect: Z = 1.99 (P = 0.05)
Test for subgroup differences: Chi² = 0.31, df = 1 (P = 0.58), I² = 0%
0.01
0.1
1
10
100
Favours [experimental]
Favours [control]

5

Study or Subgroup	Experimental Events	Experimental Total	Control Events	Control Total	Weight	Odds Ratio M-H, Random, 95% CI
**4.4.1 Simple abdominal acupuncture**						
Wang 2023	1	31	3	32	20.8%	0.32 [0.03, 3.28]
**Subtotal (95% CI)**		**31**		**32**	**20.8%**	**0.32 [0.03, 3.28]**
Total events	1		3			
Heterogeneity: Not applicable						
Test for overall effect: Z = 0.96 (P = 0.34)						
**4.4.2 Non - simple abdominal acupuncture**						
Li 2015	2	30	25	30	24.5%	0.01 [0.00, 0.08]
Li 2018	14	117	18	111	29.7%	0.70 [0.33, 1.49]
Qian 2007	2	33	9	30	25.1%	0.15 [0.03, 0.77]
**Subtotal (95% CI)**		**180**		**171**	**79.2%**	**0.13 [0.01, 1.28]**
Total events	18		52			
Heterogeneity: $Tau^2 = 3.69$; $Chi^2 = 17.83$, df = 2 (P = 0.0001); $I^2 = 89\%$						
Test for overall effect: Z = 1.75 (P = 0.08)						
**Total (95% CI)**		**211**		**203**	**100.0%**	**0.16 [0.03, 0.98]**
Total events	19		55			
Heterogeneity: $Tau^2 = 2.78$; $Chi^2 = 17.81$, df = 3 (P = 0.0005); $I^2 = 83\%$						
Test for overall effect: Z = 1.99 (P = 0.05)						
Test for subgroup differences: $Chi^2 = 0.31$, df = 1 (P = 0.58), $I^2 = 0\%$						

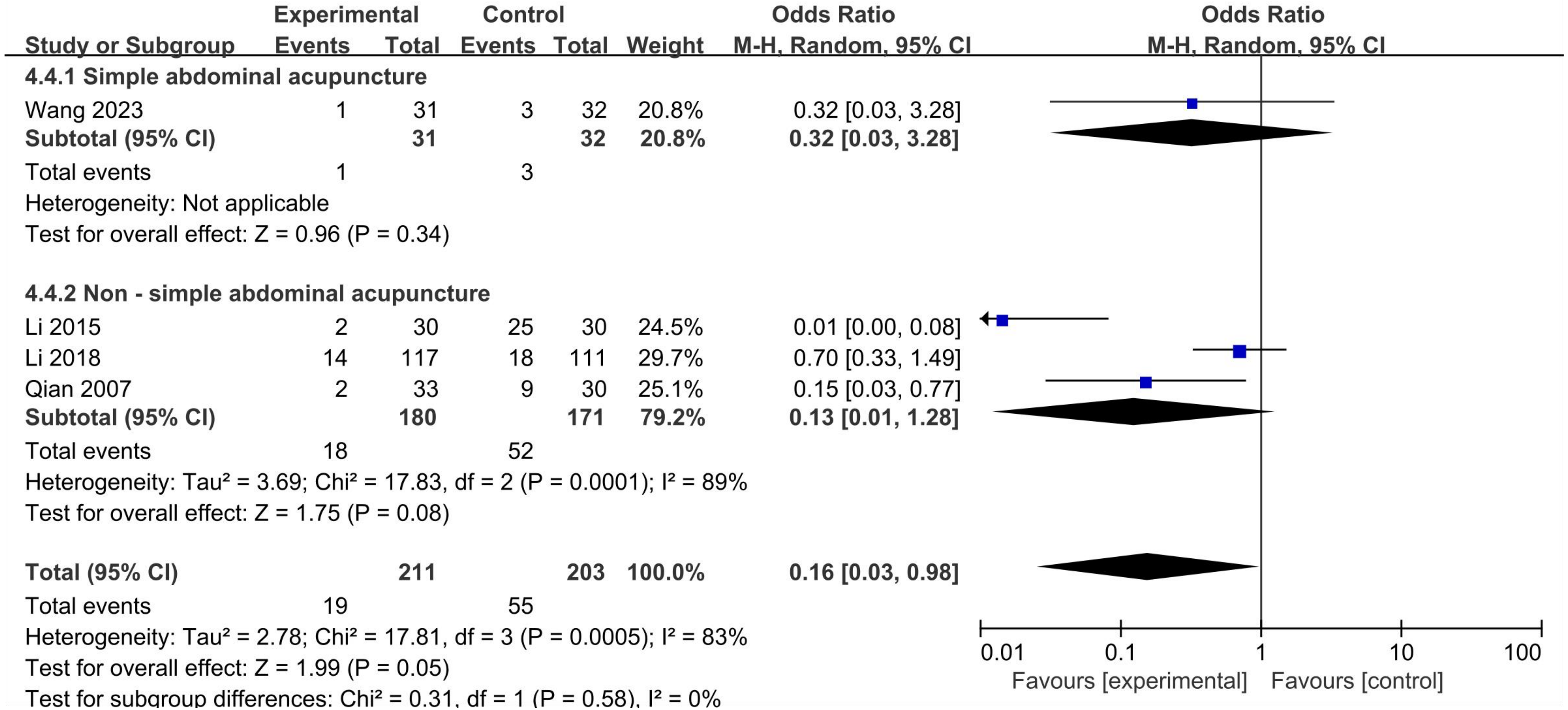

Supplement: Supplementary 1 — Clinical effectiveness rate. [file SupplementaryFile1.zip › Supplementary material 6.PDF]
