# Supplementary material for: Effect of acupuncture on menopausal depressive disorder and serum hormone levels: a systematic review and meta-analysis
Source: Front Psychiatry. 2025 Jul 14;16:1591389. doi: 10.3389/fpsyt.2025.1591389 (PMC12301320; doi:10.3389/fpsyt.2025.1591389)

Supplementary 5

Figure 1-3: The forest plot of FSH,E2,LH

Figure 4-6: The forest plot of FSH,E2,LH group analysis （The type of acupuncture in the experimental group）

Figure 7-9: The forest plot of FSH,E2,LH group analysis （Control Group Type）

1 FSH

	Experimental			Control				Std. Mean Difference
**Study or Subgroup**	**Mean**	**SD**	**Total**	**Mean**	**SD**	**Total**	**Weight**	**IV, Fixed, 95% CI**
Chen 2010	58.86	6.71	30	61.28	6.14	30	8.3%	-0.37 [-0.88, 0.14]
Li 2015	53.56	47.06	30	44.53	36.64	30	8.4%	0.21 [-0.30, 0.72]
Li 2018	41.46	32.73	116	42.2	100.7	105	31.0%	-0.01 [-0.27, 0.25]
Zhang 2010	38.18	21.1	44	39.88	24.14	46	12.6%	-0.07 [-0.49, 0.34]
Zhao 2023	39.97	20.12	35	42.67	23.01	35	9.8%	-0.12 [-0.59, 0.35]
Zhou 2022	44.04	35.65	108	44.32	35.78	104	29.8%	-0.01 [-0.28, 0.26]
**Total (95% CI)**			**363**			**350**	**100.0%**	**-0.04 [-0.19, 0.11]**

Heterogeneity: Chi² = 2.81, df = 5 (P = 0.73); I² = 0%
Test for overall effect: Z = 0.53 (P = 0.59)

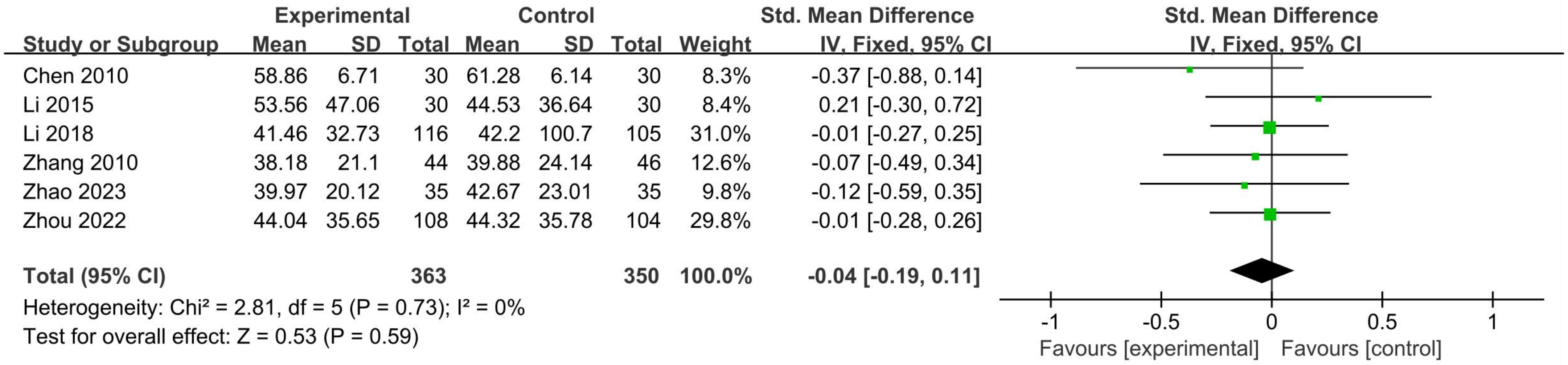

2E2

	Experimental			Control				Std. Mean Difference	Std. Mean Difference
**Study or Subgroup**	**Mean**	**SD**	**Total**	**Mean**	**SD**	**Total**	**Weight**	**IV, Fixed, 95% CI**	**IV, Fixed, 95% CI**
Chen 2010	246.37	23.71	30	238.12	24.45	30	8.3%	0.34 [-0.17, 0.85]	
Li 2015	172.38	270.96	30	167.95	169.75	30	8.4%	0.02 [-0.49, 0.53]	
Li 2018	146.21	246.95	116	146.58	260.33	105	31.0%	-0.00 [-0.27, 0.26]	
Zhang 2010	39.54	8.88	44	40.97	10.11	46	12.6%	-0.15 [-0.56, 0.27]	
Zhao 2023	154.98	83	35	160.78	76.38	35	9.8%	-0.07 [-0.54, 0.40]	
Zhou 2022	156.97	250.88	108	169.16	267.11	104	29.8%	-0.05 [-0.32, 0.22]	
**Total (95% CI)**			**363**			**350**	**100.0%**	**-0.01 [-0.16, 0.14]**	

Heterogeneity: Chi² = 2.38, df = 5 (P = 0.79); I² = 0%
Test for overall effect: Z = 0.14 (P = 0.89)

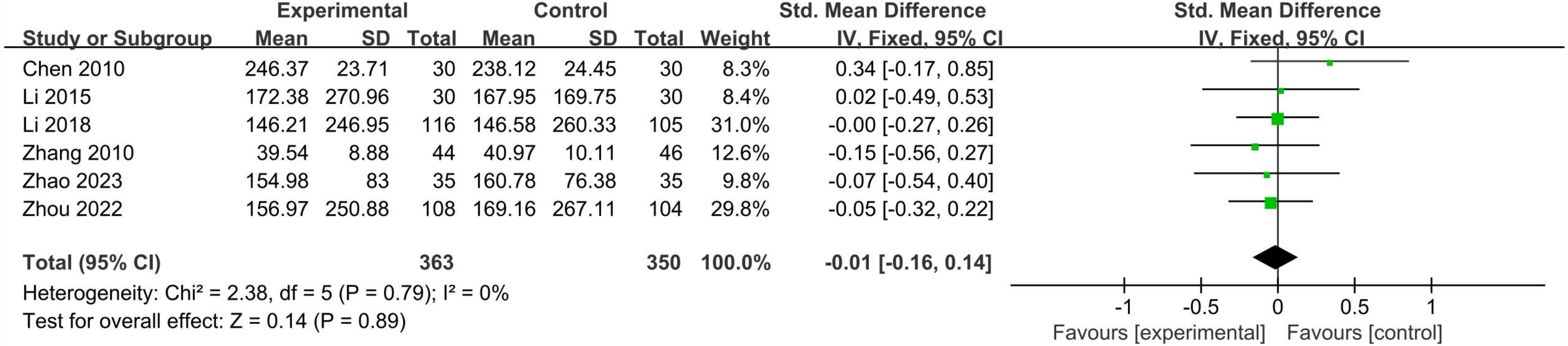

3LH

	Experimental			Control				Std. Mean Difference
Study or Subgroup	Mean	SD	Total	Mean	SD	Total	Weight	IV, Fixed, 95% CI
Li 2015	25.9	21.02	30	24.71	19.68	30	9.2%	0.06 [-0.45, 0.56]
Li 2018	22.4	16.24	116	21.33	47.31	105	33.8%	0.03 [-0.23, 0.29]
Zhang 2010	26.74	24.5	44	26.7	23.43	46	13.8%	0.00 [-0.41, 0.41]
Zhao 2023	20.39	8.82	35	20.17	9.35	35	10.7%	0.02 [-0.44, 0.49]
Zhou 2022	23.58	18.61	108	23.82	17.94	104	32.5%	-0.01 [-0.28, 0.26]
**Total (95% CI)**			**333**			**320**	**100.0%**	**0.01 [-0.14, 0.17]**

Heterogeneity: Chi² = 0.09, df = 4 (P = 1.00); I² = 0%

Test for overall effect: Z = 0.18 (P = 0.86)

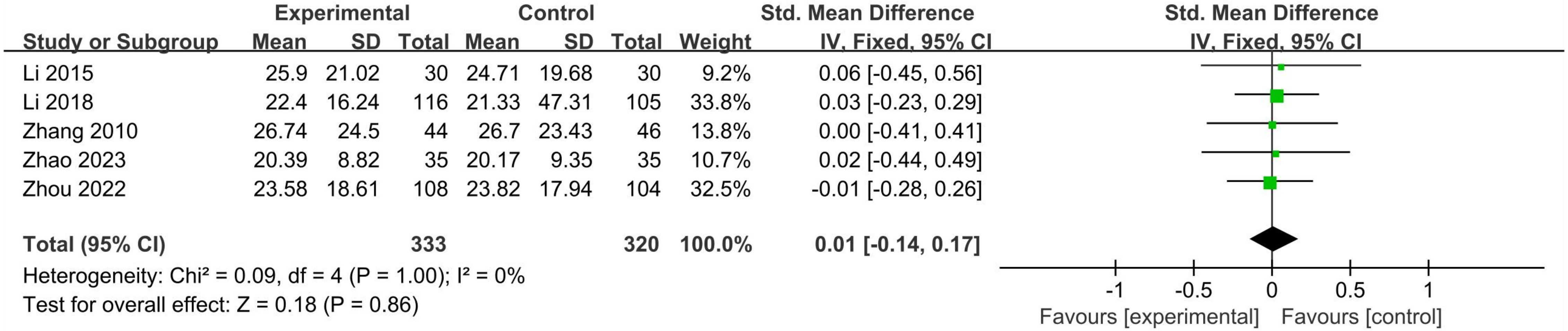

4 FSH

Study or Subgroup	Experimental Mean	Experimental SD	Experimental Total	Control Mean	Control SD	Control Total	Weight	Std. Mean Difference IV, Fixed, 95% CI
**8.2.1 EA**								
Li 2015	53.56	47.06	30	44.53	36.64	30	8.4%	0.21 [-0.30, 0.72]
Li 2018	41.46	32.73	116	42.2	100.7	105	31.0%	-0.01 [-0.27, 0.25]
Zhang 2010	38.18	21.1	44	39.88	24.14	46	12.6%	-0.07 [-0.49, 0.34]
Zhou 2022	44.04	35.65	108	44.32	35.78	104	29.8%	-0.01 [-0.28, 0.26]
**Subtotal (95% CI)**			**298**			**285**	**81.9%**	**0.00 [-0.16, 0.17]**
Heterogeneity: Chi² = 0.80, df = 3 (P = 0.85); I² = 0%								
Test for overall effect: Z = 0.04 (P = 0.97)								
**8.2.2 MA**								
Chen 2010	58.86	6.71	30	61.28	6.14	30	8.3%	-0.37 [-0.88, 0.14]
Zhao 2023	39.97	20.12	35	42.67	23.01	35	9.8%	-0.12 [-0.59, 0.35]
**Subtotal (95% CI)**			**65**			**65**	**18.1%**	**-0.24 [-0.58, 0.11]**
Heterogeneity: Chi² = 0.49, df = 1 (P = 0.48); I² = 0%								
Test for overall effect: Z = 1.34 (P = 0.18)								
**Total (95% CI)**			**363**			**350**	**100.0%**	**-0.04 [-0.19, 0.11]**
Heterogeneity: Chi² = 2.81, df = 5 (P = 0.73); I² = 0%								
Test for overall effect: Z = 0.53 (P = 0.59)								
Test for subgroup differences: Chi² = 1.52, df = 1 (P = 0.22), I² = 34.4%								

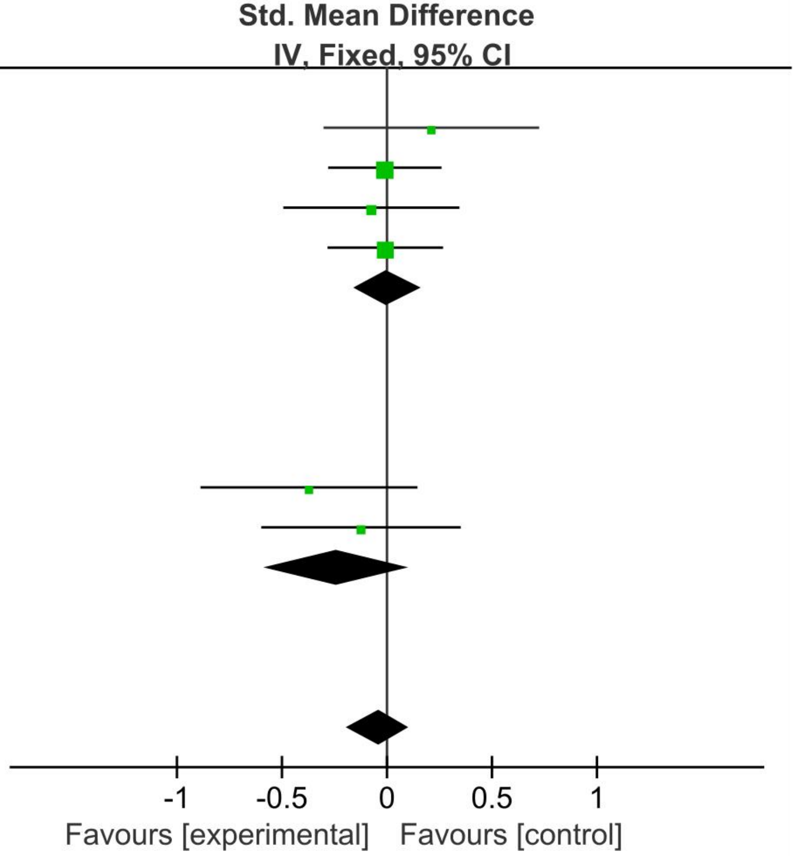

5E2

Study or Subgroup	Experimental Mean	Experimental SD	Experimental Total	Control Mean	Control SD	Control Total	Weight	Std. Mean Difference IV, Fixed, 95% CI
**9.2.1 EA**								
Zhou 2022	172.38	270.96	30	167.95	169.75	30	8.4%	0.02 [-0.49, 0.53]
Zhang 2010	146.21	246.95	116	146.58	260.33	105	31.0%	-0.00 [-0.27, 0.26]
Li 2018	39.54	8.88	44	40.97	10.11	46	12.6%	-0.15 [-0.56, 0.27]
Li 2015	156.97	250.88	108	169.16	267.11	104	29.8%	-0.05 [-0.32, 0.22]
**Subtotal (95% CI)**			**298**			**285**	**81.9%**	**-0.04 [-0.20, 0.12]**
Heterogeneity: Chi² = 0.40, df = 3 (P = 0.94); I² = 0%								
Test for overall effect: Z = 0.47 (P = 0.64)								
**9.2.2 MA**								
Zhao 2023	246.37	23.71	30	238.12	24.45	30	8.3%	0.34 [-0.17, 0.85]
Chen 2010	154.98	83	35	160.78	76.38	35	9.8%	-0.07 [-0.54, 0.40]
**Subtotal (95% CI)**			**65**			**65**	**18.1%**	**0.12 [-0.23, 0.46]**
Heterogeneity: Chi² = 1.35, df = 1 (P = 0.25); I² = 26%								
Test for overall effect: Z = 0.66 (P = 0.51)								
**Total (95% CI)**			**363**			**350**	**100.0%**	**-0.01 [-0.16, 0.14]**
Heterogeneity: Chi² = 2.38, df = 5 (P = 0.79); I² = 0%								
Test for overall effect: Z = 0.14 (P = 0.89)								
Test for subgroup differences: Chi² = 0.63, df = 1 (P = 0.43), I² = 0%								

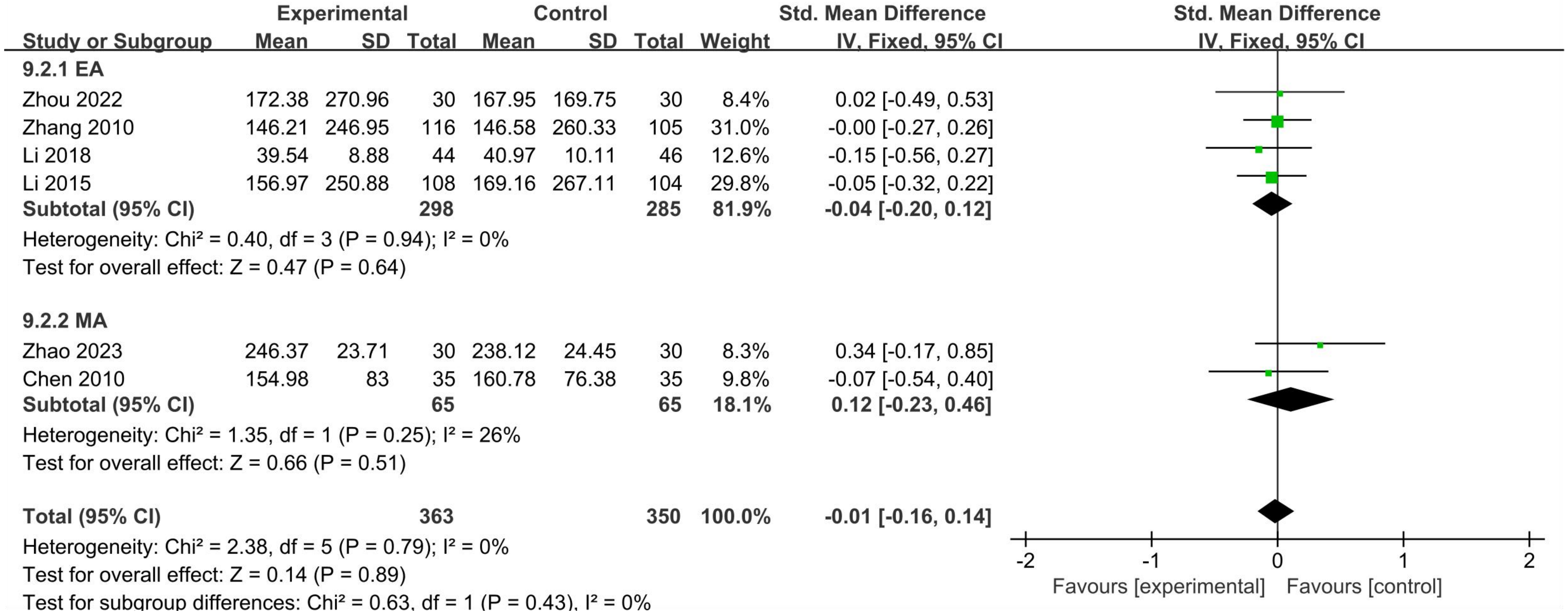

6 LH

Study or Subgroup	Experimental Mean	Experimental SD	Experimental Total	Control Mean	Control SD	Control Total	Weight	Std. Mean Difference IV, Fixed, 95% CI
**10.2.1 EA**								
Li 2015	25.9	21.02	30	24.71	19.68	30	9.2%	0.06 [-0.45, 0.56]
Li 2018	22.4	16.24	116	21.33	47.31	105	33.8%	0.03 [-0.23, 0.29]
Zhang 2010	26.74	24.5	44	26.7	23.43	46	13.8%	0.00 [-0.41, 0.41]
Zhou 2022	23.58	18.61	108	23.82	17.94	104	32.5%	-0.01 [-0.28, 0.26]
**Subtotal (95% CI)**			**298**			**285**	**89.3%**	**0.01 [-0.15, 0.18]**
Heterogeneity: Chi² = 0.09, df = 3 (P = 0.99); I² = 0%								
Test for overall effect: Z = 0.16 (P = 0.87)								
**10.2.2 MA**								
Zhao 2023	20.39	8.82	35	20.17	9.35	35	10.7%	0.02 [-0.44, 0.49]
**Subtotal (95% CI)**			**35**			**35**	**10.7%**	**0.02 [-0.44, 0.49]**
Heterogeneity: Not applicable								
Test for overall effect: Z = 0.10 (P = 0.92)								
**Total (95% CI)**			**333**			**320**	**100.0%**	**0.01 [-0.14, 0.17]**
Heterogeneity: Chi² = 0.09, df = 4 (P = 1.00); I² = 0%								
Test for overall effect: Z = 0.18 (P = 0.86)								
Test for subgroup differences: Chi² = 0.00, df = 1 (P = 0.97), I² = 0%								

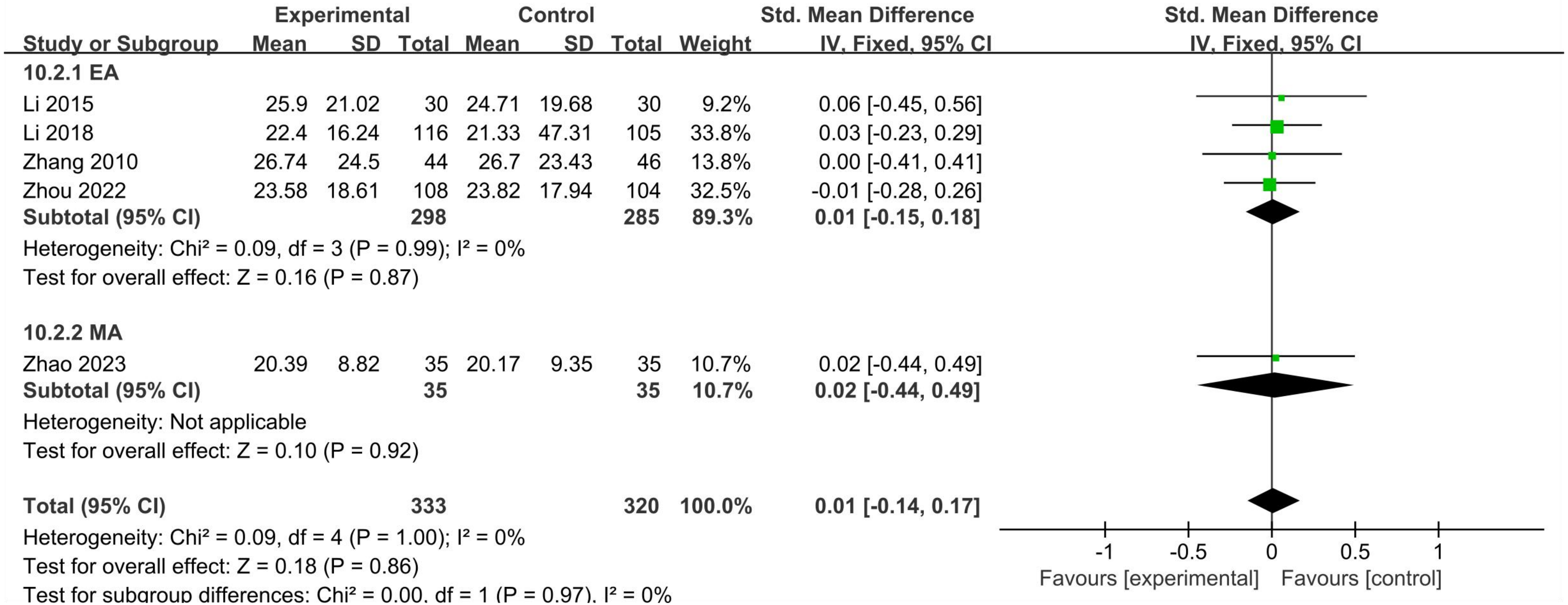

7 FSH

Study or Subgroup	Experimental Mean	Experimental SD	Experimental Total	Control Mean	Control SD	Control Total	Weight	Std. Mean Difference IV, Fixed, 95% CI
**8.4.1 CHM**								
Chen 2010	58.86	6.71	30	61.28	6.14	30	8.3%	-0.37 [-0.88, 0.14]
**Subtotal (95% CI)**			**30**			**30**	**8.3%**	**-0.37 [-0.88, 0.14]**
Heterogeneity: Not applicable								
Test for overall effect: Z = 1.43 (P = 0.15)								
**8.4.2 other medications**								
Li 2015	53.56	47.06	30	44.53	36.64	30	8.4%	0.21 [-0.30, 0.72]
Li 2018	41.46	32.73	116	42.2	100.7	105	31.0%	-0.01 [-0.27, 0.25]
Zhang 2010	38.18	21.1	44	39.88	24.14	46	12.6%	-0.07 [-0.49, 0.34]
Zhou 2022	44.04	35.65	108	44.32	35.78	104	29.8%	-0.01 [-0.28, 0.26]
**Subtotal (95% CI)**			**298**			**285**	**81.9%**	**0.00 [-0.16, 0.17]**
Heterogeneity: Chi² = 0.80, df = 3 (P = 0.85); I² = 0%								
Test for overall effect: Z = 0.04 (P = 0.97)								
**8.4.3 SA**								
Zhao 2023	39.97	20.12	35	42.67	23.01	35	9.8%	-0.12 [-0.59, 0.35]
**Subtotal (95% CI)**			**35**			**35**	**9.8%**	**-0.12 [-0.59, 0.35]**
Heterogeneity: Not applicable								
Test for overall effect: Z = 0.52 (P = 0.61)								
**Total (95% CI)**			**363**			**350**	**100.0%**	**-0.04 [-0.19, 0.11]**
Heterogeneity: Chi² = 2.81, df = 5 (P = 0.73); I² = 0%								
Test for overall effect: Z = 0.53 (P = 0.59)								
Test for subgroup differences: Chi² = 2.02, df = 2 (P = 0.37), I² = 0.8%								

Std. Mean Difference IV, Fixed, 95% CI

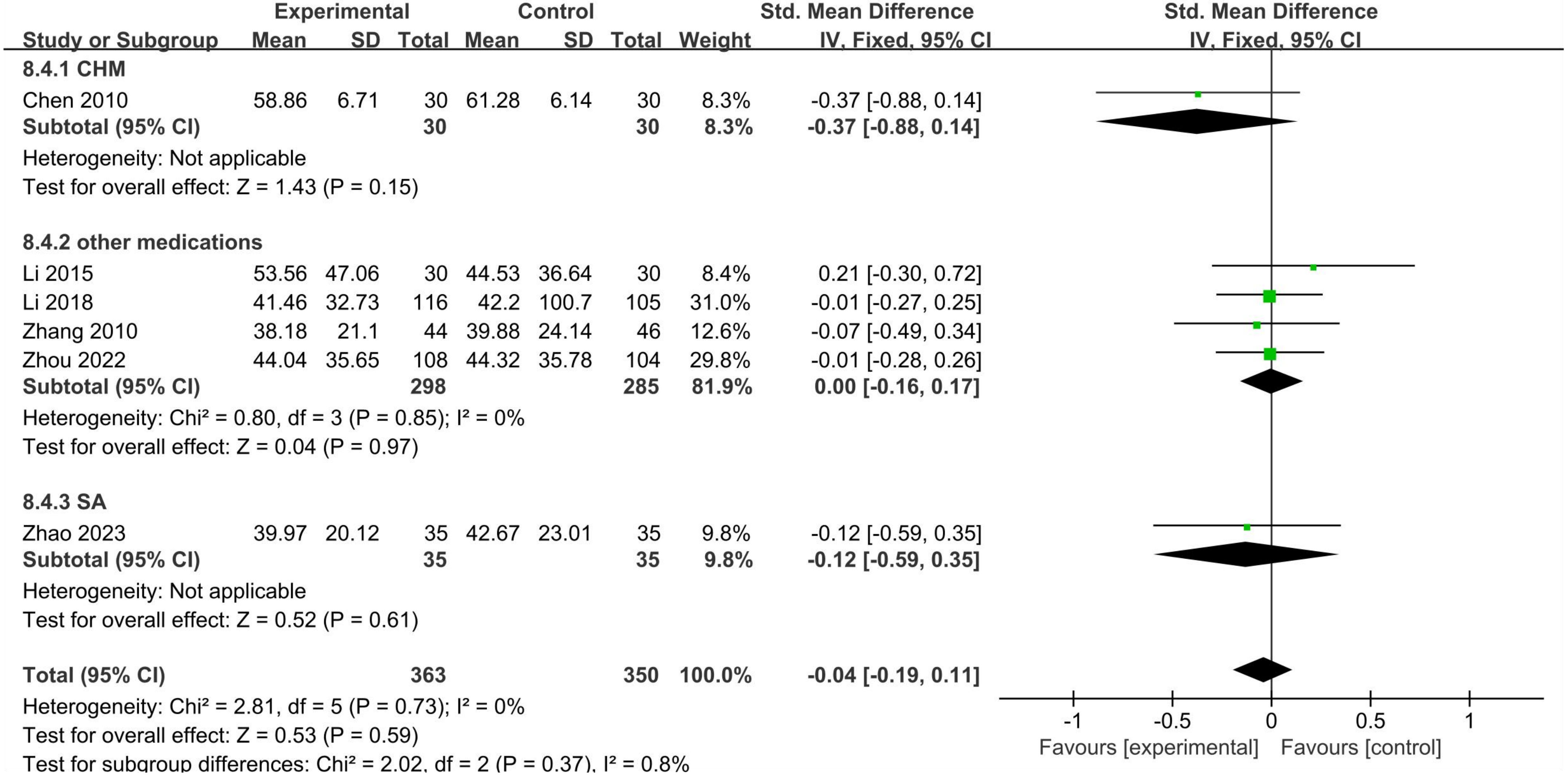

8E2

Study or Subgroup	Experimental Mean	Experimental SD	Experimental Total	Control Mean	Control SD	Control Total	Weight	Std. Mean Difference IV, Fixed, 95% CI
**9.4.1 CHM**								
Chen 2010	246.37	23.71	30	238.12	24.45	30	8.3%	0.34 [-0.17, 0.85]
**Subtotal (95% CI)**			**30**			**30**	**8.3%**	**0.34 [-0.17, 0.85]**
Heterogeneity: Not applicable								
Test for overall effect: Z = 1.30 (P = 0.19)								
**9.4.2 other medications**								
Li 2015	172.38	270.96	30	167.95	169.75	30	8.4%	0.02 [-0.49, 0.53]
Li 2018	146.21	246.95	116	146.58	260.33	105	31.0%	-0.00 [-0.27, 0.26]
Zhang 2010	39.54	8.88	44	40.97	10.11	46	12.6%	-0.15 [-0.56, 0.27]
Zhou 2022	156.97	250.88	108	169.16	267.11	104	29.8%	-0.05 [-0.32, 0.22]
**Subtotal (95% CI)**			**298**			**285**	**81.9%**	**-0.04 [-0.20, 0.12]**
Heterogeneity: Chi² = 0.40, df = 3 (P = 0.94); I² = 0%								
Test for overall effect: Z = 0.47 (P = 0.64)								
**9.4.3 SA**								
Zhao 2023	154.98	83	35	160.78	76.38	35	9.8%	-0.07 [-0.54, 0.40]
**Subtotal (95% CI)**			**35**			**35**	**9.8%**	**-0.07 [-0.54, 0.40]**
Heterogeneity: Not applicable								
Test for overall effect: Z = 0.30 (P = 0.76)								
**Total (95% CI)**			**363**			**350**	**100.0%**	**-0.01 [-0.16, 0.14]**
Heterogeneity: Chi² = 2.38, df = 5 (P = 0.79); I² = 0%								
Test for overall effect: Z = 0.14 (P = 0.89)								
Test for subgroup differences: Chi² = 1.98, df = 2 (P = 0.37), I² = 0%								

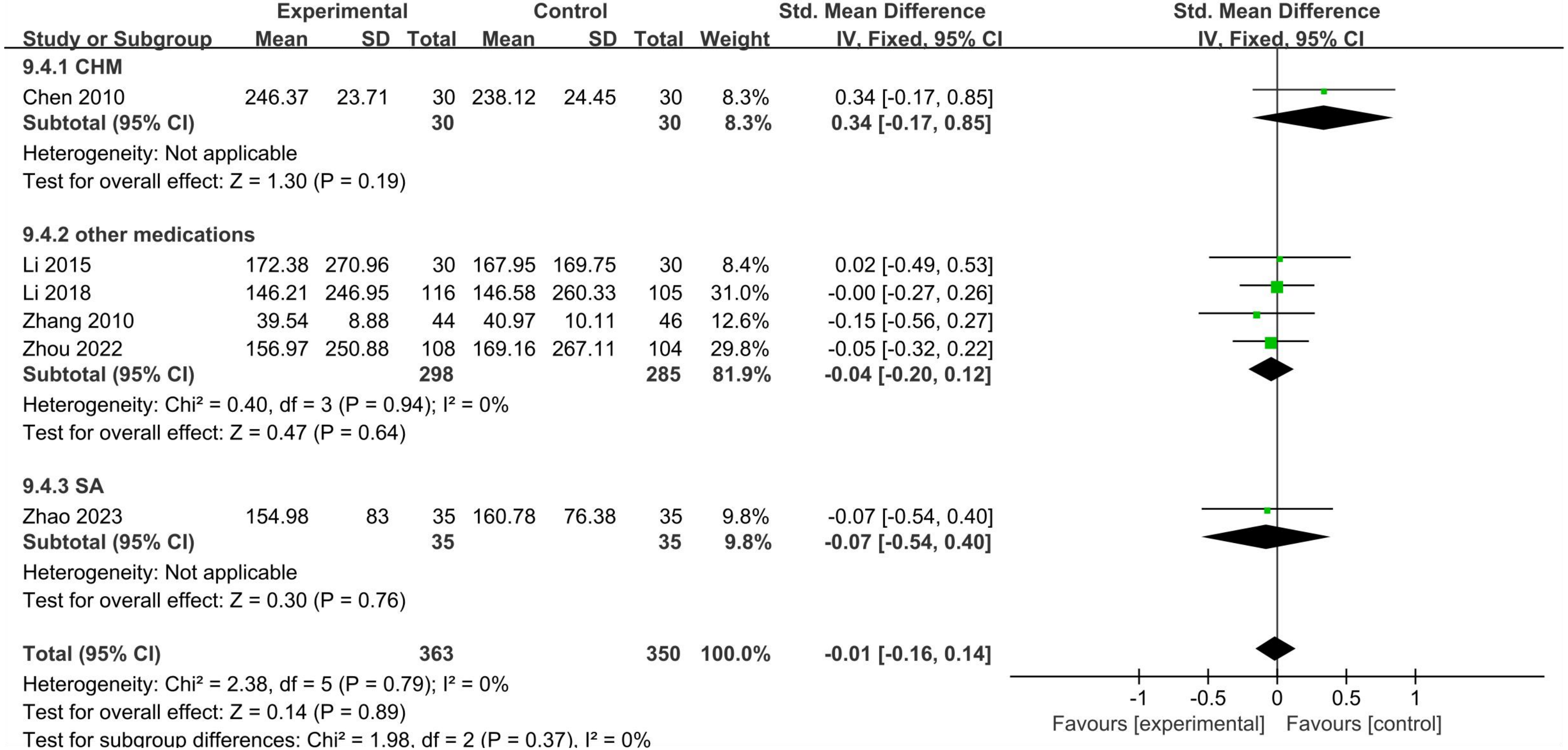

9 LH

Study or Subgroup	Experimental Mean	Experimental SD	Experimental Total	Control Mean	Control SD	Control Total	Weight	Std. Mean Difference IV, Fixed, 95% CI
**10.2.1 other medications**								
Li 2015	25.9	21.02	30	24.71	19.68	30	9.2%	0.06 [-0.45, 0.56]
Li 2018	22.4	16.24	116	21.33	47.31	105	33.8%	0.03 [-0.23, 0.29]
Zhang 2010	26.74	24.5	44	26.7	23.43	46	13.8%	0.00 [-0.41, 0.41]
Zhou 2022	23.58	18.61	108	23.82	17.94	104	32.5%	-0.01 [-0.28, 0.26]
**Subtotal (95% CI)**			**298**			**285**	**89.3%**	**0.01 [-0.15, 0.18]**
Heterogeneity: Chi² = 0.09, df = 3 (P = 0.99); I² = 0%								
Test for overall effect: Z = 0.16 (P = 0.87)								
**10.2.2 SA**								
Zhao 2023	20.39	8.82	35	20.17	9.35	35	10.7%	0.02 [-0.44, 0.49]
**Subtotal (95% CI)**			**35**			**35**	**10.7%**	**0.02 [-0.44, 0.49]**
Heterogeneity: Not applicable								
Test for overall effect: Z = 0.10 (P = 0.92)								
**Total (95% CI)**			**333**			**320**	**100.0%**	**0.01 [-0.14, 0.17]**
Heterogeneity: Chi² = 0.09, df = 4 (P = 1.00); I² = 0%								
Test for overall effect: Z = 0.18 (P = 0.86)								
Test for subgroup differences: Chi² = 0.00, df = 1 (P = 0.97), I² = 0%								

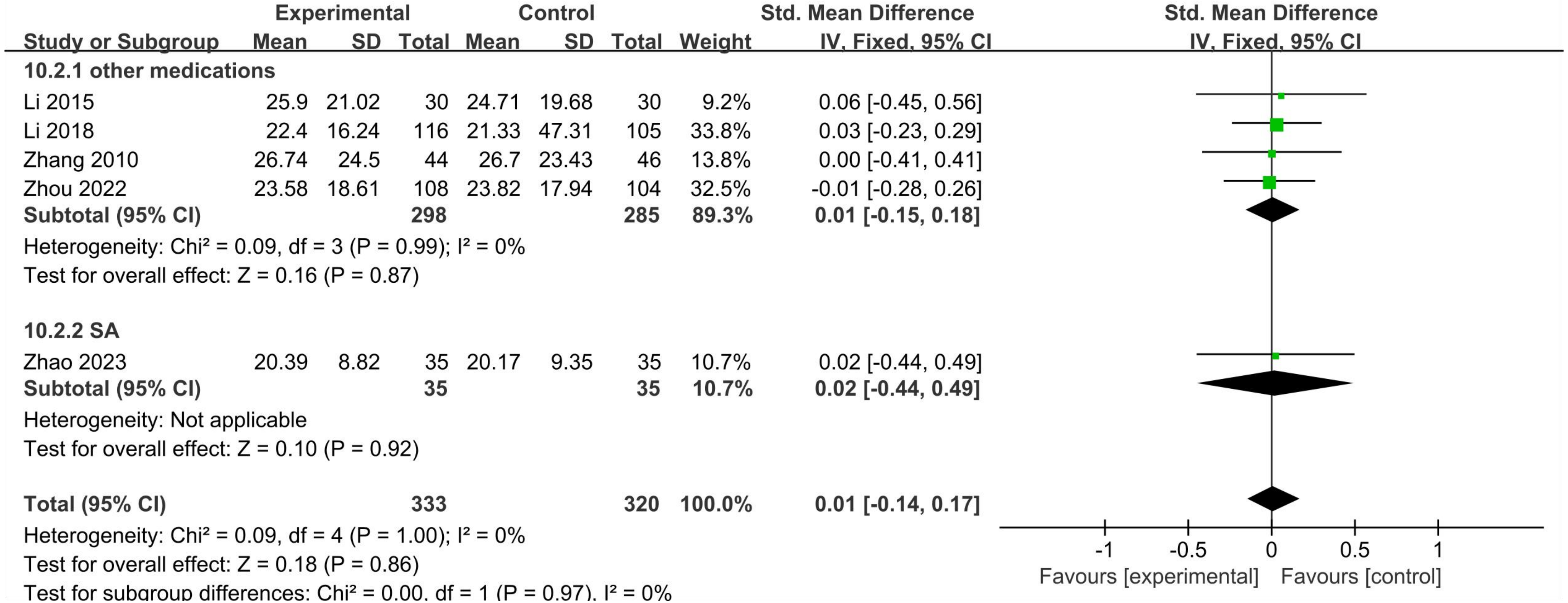

Supplement: Supplementary 1 — Clinical effectiveness rate. [file SupplementaryFile1.zip › Supplementary material 5.PDF]
