# Supplementary material for: Effect of acupuncture on menopausal depressive disorder and serum hormone levels: a systematic review and meta-analysis
Source: Front Psychiatry. 2025 Jul 14;16:1591389. doi: 10.3389/fpsyt.2025.1591389 (PMC12301320; doi:10.3389/fpsyt.2025.1591389)

Supplementary 4

Figure 1: The forest plot of SDS

Figure 2-4: The forest plot of SDS sensitivity analysis

Figure 5: The forest plot of SDS group analysis （Acupuncture sites）

1

Study or Subgroup	Experimental Mean	Experimental SD	Experimental Total	Control Mean	Control SD	Control Total	Weight	Std. Mean Difference IV, Random, 95% CI
Gu 2018	33.91	3.42	30	49.66	2.57	28	31.4%	-5.11 [-6.20, -4.02]
Wang 2015	44	3.03	33	49.15	2.31	34	34.2%	-1.89 [-2.48, -1.31]
Wang 2023	45.96	6.37	31	55.88	10.57	32	34.4%	-1.12 [-1.65, -0.58]
**Total (95% CI)**			**94**			**94**	**100.0%**	**-2.64 [-4.44, -0.84]**

Heterogeneity: $Tau^2 = 2.37$; $Chi^2 = 41.43$, df = 2 ($P < 0.00001$); $I^2 = 95\%$

Test for overall effect: $Z = 2.87$ ($P = 0.004$)

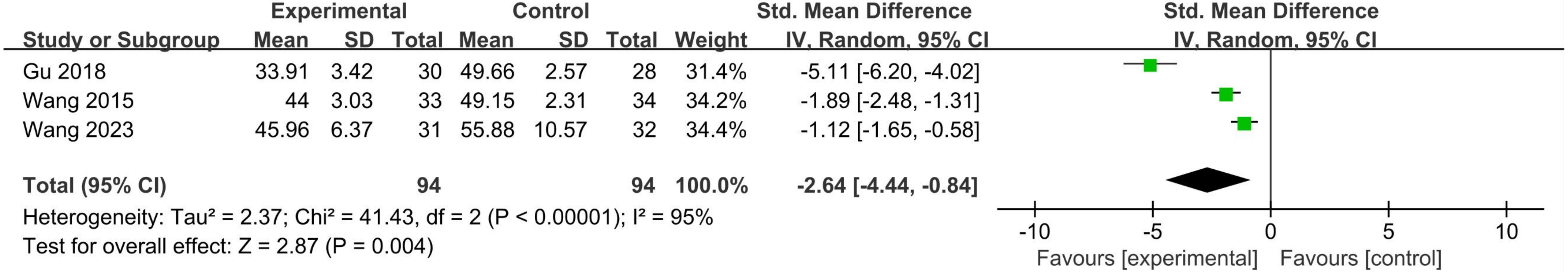

2

Study or Subgroup	Experimental Mean	Experimental SD	Experimental Total	Control Mean	Control SD	Control Total	Weight	Std. Mean Difference IV, Random, 95% CI
Gu 2018	33.91	3.42	30	49.66	2.57	28	0.0%	-5.11 [-6.20, -4.02]
Wang 2015	44	3.03	33	49.15	2.31	34	48.8%	-1.89 [-2.48, -1.31]
Wang 2023	45.96	6.37	31	55.88	10.57	32	51.2%	-1.12 [-1.65, -0.58]
**Total (95% CI)**			**64**			**66**	**100.0%**	**-1.50 [-2.26, -0.74]**

Heterogeneity: Tau² = 0.22; Chi² = 3.70, df = 1 (P = 0.05); I² = 73%
Test for overall effect: Z = 3.86 (P = 0.0001)

Std. Mean Difference
IV, Random, 95% CI

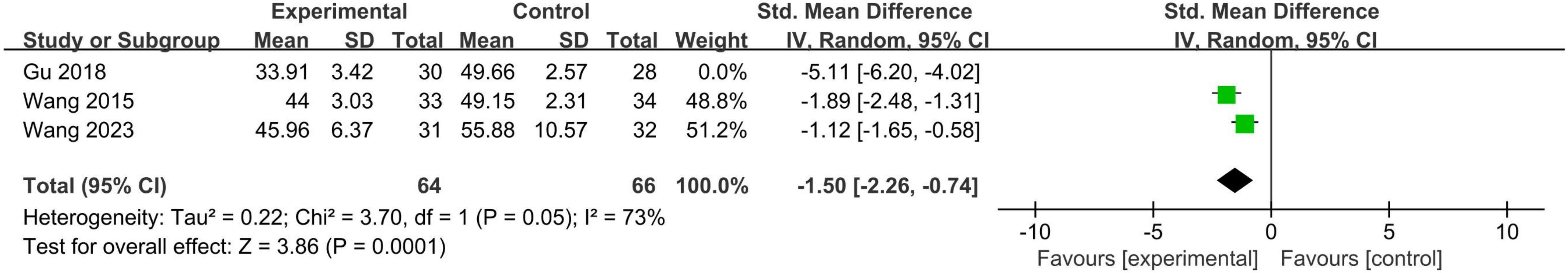

3

Study or Subgroup	Experimental Mean	Experimental SD	Experimental Total	Control Mean	Control SD	Control Total	Weight	Std. Mean Difference IV, Random, 95% CI
Gu 2018	33.91	3.42	30	49.66	2.57	28	49.3%	-5.11 [-6.20, -4.02]
Wang 2015	44	3.03	33	49.15	2.31	34	0.0%	-1.89 [-2.48, -1.31]
Wang 2023	45.96	6.37	31	55.88	10.57	32	50.7%	-1.12 [-1.65, -0.58]
**Total (95% CI)**			**61**			**60**	**100.0%**	**-3.09 [-7.00, 0.83]**

Heterogeneity: Tau² = 7.78; Chi² = 41.43, df = 1 (P < 0.00001); I² = 98%
Test for overall effect: Z = 1.55 (P = 0.12)

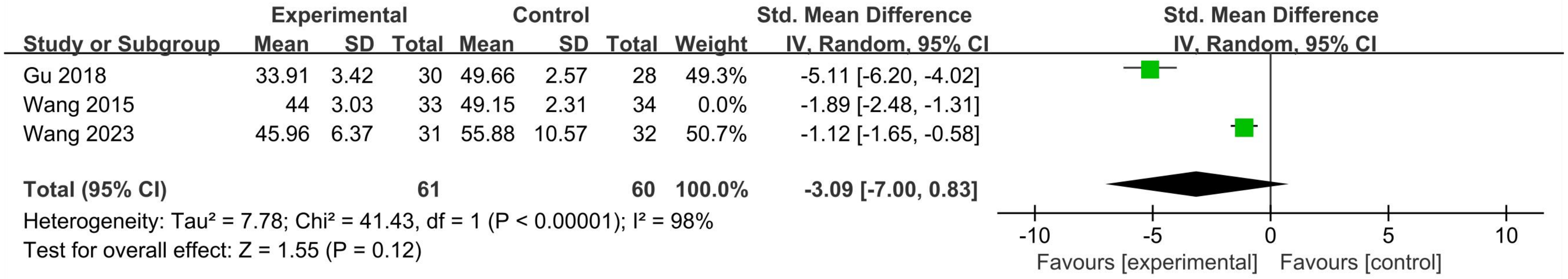

4

Study or Subgroup	Experimental Mean	Experimental SD	Experimental Total	Control Mean	Control SD	Control Total	Weight	Std. Mean Difference IV, Random, 95% CI
Gu 2018	33.91	3.42	30	49.66	2.57	28	48.9%	-5.11 [-6.20, -4.02]
Wang 2015	44	3.03	33	49.15	2.31	34	51.1%	-1.89 [-2.48, -1.31]
Wang 2023	45.96	6.37	31	55.88	10.57	32	0.0%	-1.12 [-1.65, -0.58]
**Total (95% CI)**			**63**			**62**	**100.0%**	**-3.47 [-6.62, -0.31]**

Heterogeneity: $Tau^2 = 4.98$; $Chi^2 = 25.97$, df = 1 ($P < 0.00001$); $I^2 = 96\%$

Test for overall effect: Z = 2.16 (P = 0.03)

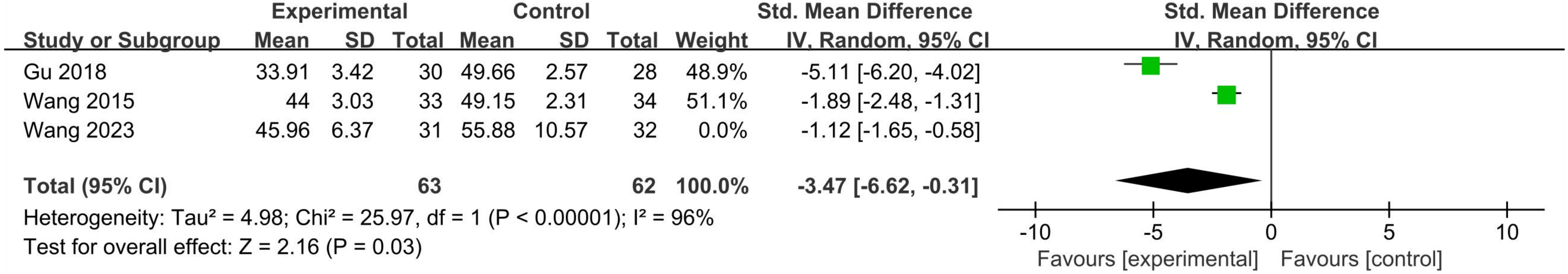

5

Study or Subgroup	Experimental Mean	Experimental SD	Experimental Total	Control Mean	Control SD	Control Total	Weight	Std. Mean Difference IV, Random, 95% CI
**7.4.1 Simple abdominal acupuncture**								
Wang 2023	45.96	6.37	31	55.88	10.57	32	34.4%	-1.12 [-1.65, -0.58]
**Subtotal (95% CI)**			**31**			**32**	**34.4%**	**-1.12 [-1.65, -0.58]**
Heterogeneity: Not applicable								
Test for overall effect: Z = 4.11 (P < 0.0001)								
**7.4.2 Non - simple abdominal acupuncture**								
Gu 2018	33.91	3.42	30	49.66	2.57	28	31.4%	-5.11 [-6.20, -4.02]
Wang 2015	44	3.03	33	49.15	2.31	34	34.2%	-1.89 [-2.48, -1.31]
**Subtotal (95% CI)**			**63**			**62**	**65.6%**	**-3.47 [-6.62, -0.31]**
Heterogeneity: $Tau^2 = 4.98$; $Chi^2 = 25.97$, df = 1 (P < 0.00001); $I^2 = 96\%$								
Test for overall effect: Z = 2.16 (P = 0.03)								
**Total (95% CI)**			**94**			**94**	**100.0%**	**-2.64 [-4.44, -0.84]**
Heterogeneity: $Tau^2 = 2.37$; $Chi^2 = 41.43$, df = 2 (P < 0.00001); $I^2 = 95\%$								
Test for overall effect: Z = 2.87 (P = 0.004)								
Test for subgroup differences: $Chi^2 = 2.07$, df = 1 (P = 0.15), $I^2 = 51.8\%$								

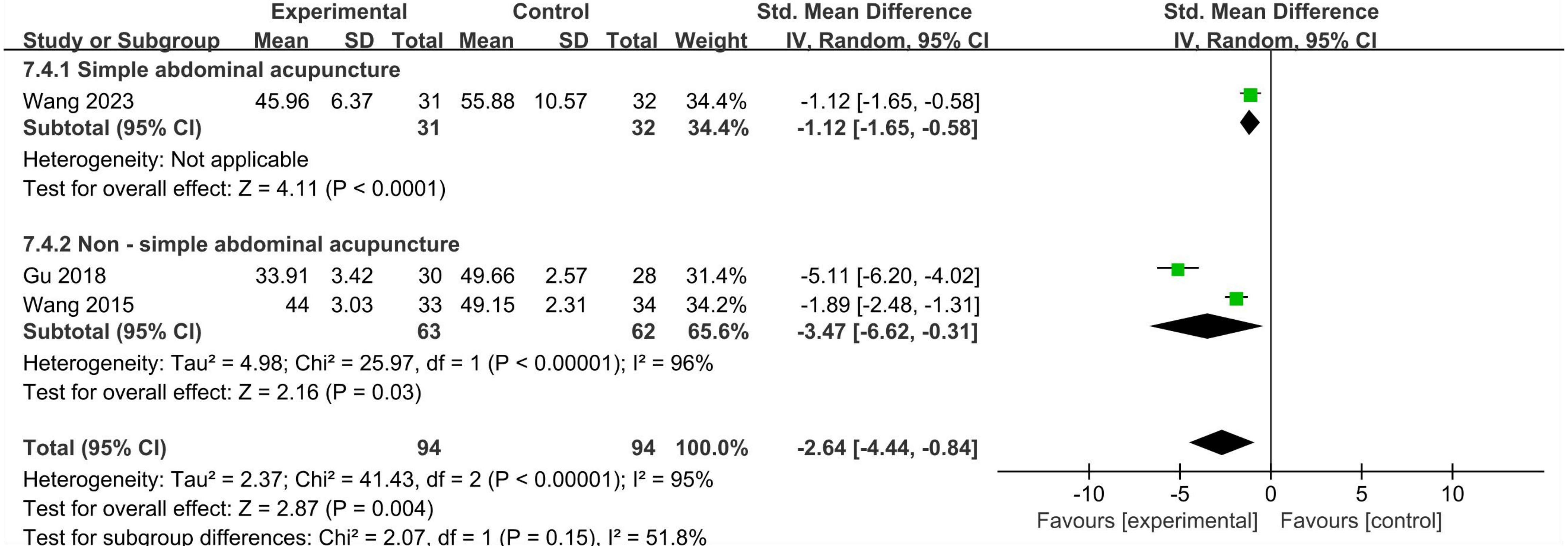

Supplement: Supplementary 1 — Clinical effectiveness rate. [file SupplementaryFile1.zip › Supplementary material 4.PDF]
