# Supplementary material for: Effect of acupuncture on menopausal depressive disorder and serum hormone levels: a systematic review and meta-analysis
Source: Front Psychiatry. 2025 Jul 14;16:1591389. doi: 10.3389/fpsyt.2025.1591389 (PMC12301320; doi:10.3389/fpsyt.2025.1591389)

Supplementary 3

Figure 1: The forest plot of HAMD-24

Figure 2: The forest plot of HAMD-24 sensitivity analysis

Figure 3: The forest plot of HAMD-24 group analysis （The type of acupuncture in the experimental group）

Figure 4: The forest plot of HAMD-24 group analysis （Control Group Type）

1

Study or Subgroup	Experimental Mean	Experimental SD	Experimental Total	Control Mean	Control SD	Control Total	Weight	Std. Mean Difference IV, Random, 95% CI
Chen 2010	6.47	3.65	30	8.92	4.05	30	32.4%	-0.63 [-1.15, -0.11]
Gu 2018	5.15	1.29	30	7.02	1.93	28	30.8%	-1.13 [-1.69, -0.57]
Zhang 2010	7.42	2.8	44	8.02	2.08	46	36.9%	-0.24 [-0.66, 0.17]
**Total (95% CI)**			**104**			**104**	**100.0%**	**-0.64 [-1.15, -0.13]**

Heterogeneity: $Tau^2 = 0.14$; $Chi^2 = 6.34$, df = 2 (P = 0.04); $I^2 = 68\%$

Test for overall effect: Z = 2.47 (P = 0.01)

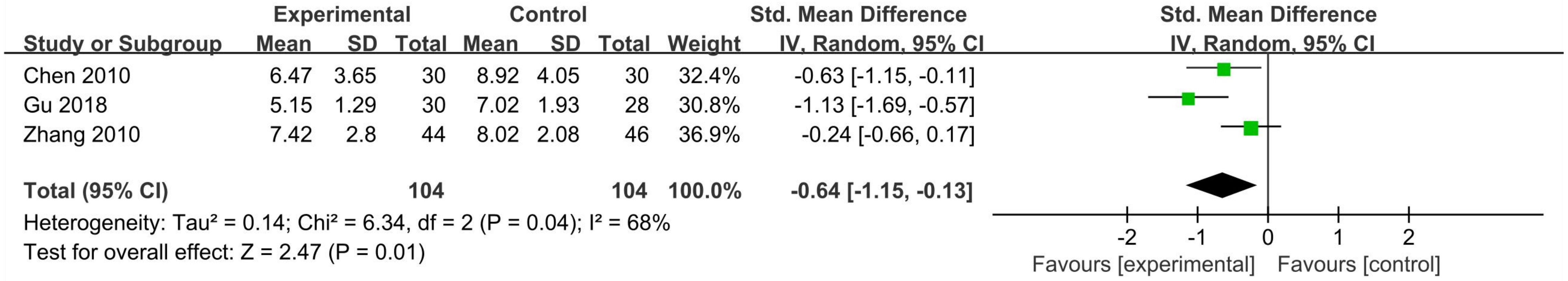

2

Study or Subgroup	Experimental Mean	Experimental SD	Experimental Total	Control Mean	Control SD	Control Total	Weight	Std. Mean Difference IV, Fixed, 95% CI
Chen 2010	6.47	3.65	30	8.92	4.05	30	39.0%	-0.63 [-1.15, -0.11]
Gu 2018	5.15	1.29	30	7.02	1.93	28	0.0%	-1.13 [-1.69, -0.57]
Zhang 2010	7.42	2.8	44	8.02	2.08	46	61.0%	-0.24 [-0.66, 0.17]
**Total (95% CI)**			**74**			**76**	**100.0%**	**-0.39 [-0.72, -0.07]**

Heterogeneity: Chi² = 1.29, df = 1 (P = 0.26); I² = 23%

Test for overall effect: Z = 2.37 (P = 0.02)

Std. Mean Difference
IV, Fixed, 95% CI

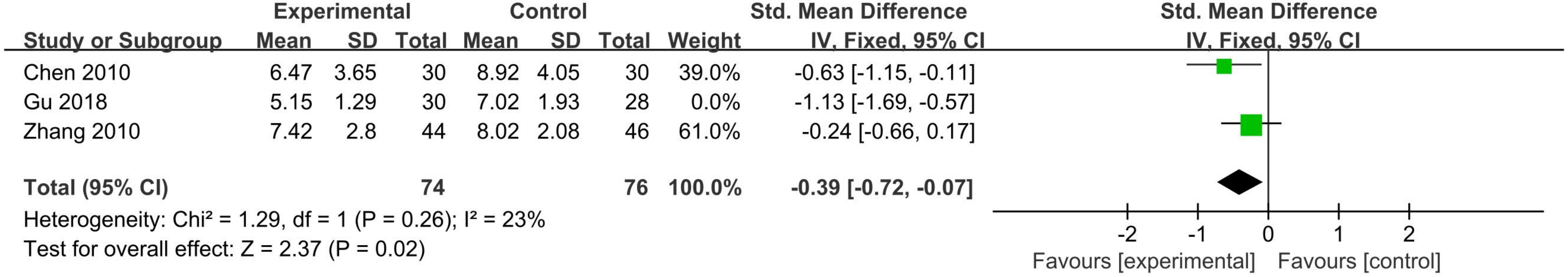

3

Study or Subgroup	Experimental Mean	Experimental SD	Experimental Total	Control Mean	Control SD	Control Total	Weight	Std. Mean Difference IV, Random, 95% CI
**2.3.1 EA**								
Zhang 2010	7.42	2.8	44	8.02	2.08	46	36.9%	-0.24 [-0.66, 0.17]
**Subtotal (95% CI)**			**44**			**46**	**36.9%**	**-0.24 [-0.66, 0.17]**
Heterogeneity: Not applicable								
Test for overall effect: Z = 1.14 (P = 0.25)								
**2.3.2 MA**								
Chen 2010	6.47	3.65	30	8.92	4.05	30	32.4%	-0.63 [-1.15, -0.11]
Gu 2018	5.15	1.29	30	7.02	1.93	28	30.8%	-1.13 [-1.69, -0.57]
**Subtotal (95% CI)**			**60**			**58**	**63.1%**	**-0.87 [-1.36, -0.37]**
Heterogeneity: Tau² = 0.05; Chi² = 1.68, df = 1 (P = 0.19); I² = 41%								
Test for overall effect: Z = 3.45 (P = 0.0006)								
**Total (95% CI)**			**104**			**104**	**100.0%**	**-0.64 [-1.15, -0.13]**
Heterogeneity: Tau² = 0.14; Chi² = 6.34, df = 2 (P = 0.04); I² = 68%								
Test for overall effect: Z = 2.47 (P = 0.01)								
Test for subgroup differences: Chi² = 3.63, df = 1 (P = 0.06), I² = 72.4%								

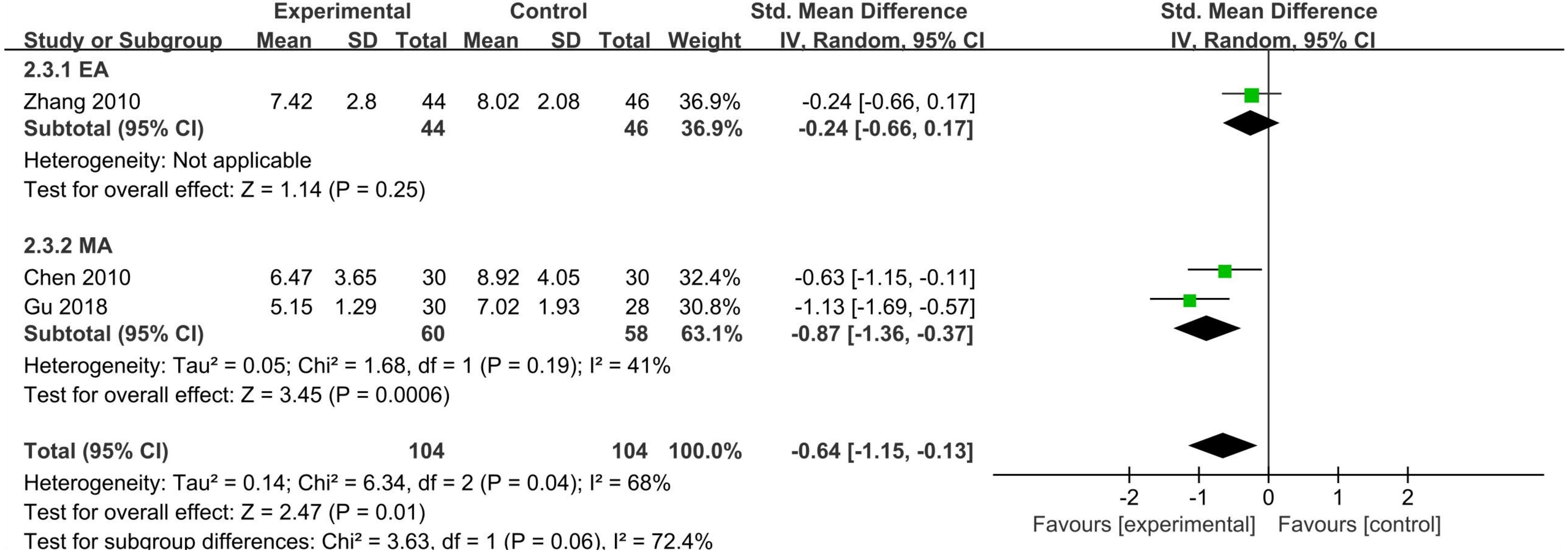

4

Study or Subgroup	Experimental Mean	Experimental SD	Experimental Total	Control Mean	Control SD	Control Total	Weight	Std. Mean Difference IV, Random, 95% CI
**2.2.1 CHM**								
Chen 2010	6.47	3.65	30	8.92	4.05	30	32.4%	-0.63 [-1.15, -0.11]
Gu 2018	5.15	1.29	30	7.02	1.93	28	30.8%	-1.13 [-1.69, -0.57]
**Subtotal (95% CI)**			**60**			**58**	**63.1%**	**-0.87 [-1.36, -0.37]**
Heterogeneity: Tau² = 0.05; Chi² = 1.68, df = 1 (P = 0.19); I² = 41%								
Test for overall effect: Z = 3.45 (P = 0.0006)								
**2.2.2 other medications**								
Zhang 2010	7.42	2.8	44	8.02	2.08	46	36.9%	-0.24 [-0.66, 0.17]
**Subtotal (95% CI)**			**44**			**46**	**36.9%**	**-0.24 [-0.66, 0.17]**
Heterogeneity: Not applicable								
Test for overall effect: Z = 1.14 (P = 0.25)								
**Total (95% CI)**			**104**			**104**	**100.0%**	**-0.64 [-1.15, -0.13]**

Heterogeneity: Tau² = 0.14; Chi² = 6.34, df = 2 (P = 0.04); I² = 68%
Test for overall effect: Z = 2.47 (P = 0.01)
Test for subgroup differences: Chi² = 3.63, df = 1 (P = 0.06), I² = 72.4%

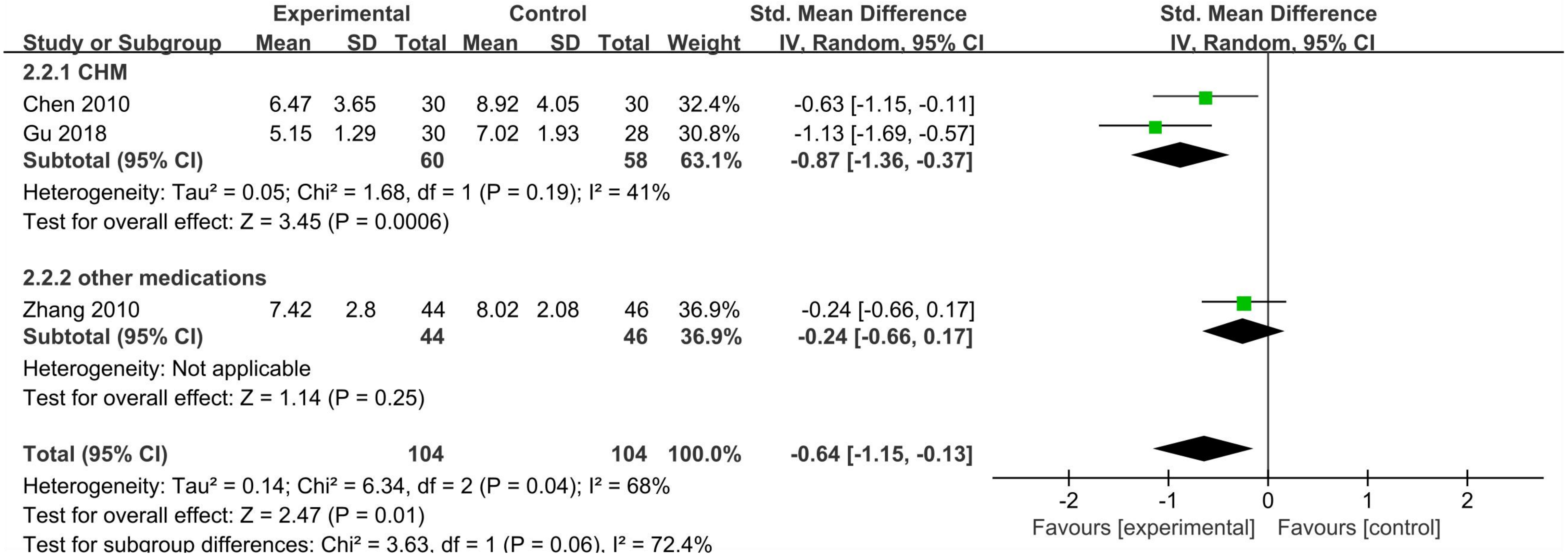

Supplement: Supplementary 1 — Clinical effectiveness rate. [file SupplementaryFile1.zip › Supplementary material 3.PDF]
