# Supplementary material for: Effect of acupuncture on menopausal depressive disorder and serum hormone levels: a systematic review and meta-analysis
Source: Front Psychiatry. 2025 Jul 14;16:1591389. doi: 10.3389/fpsyt.2025.1591389 (PMC12301320; doi:10.3389/fpsyt.2025.1591389)

Supplementary 2

Figure 1: The forest plot of HAMD-17

Figure 2: The forest plot of HAMD-17 group analysis （The type of acupuncture in the experimental group） Acupuncture sites

Figure 3: The forest plot of HAMD-17 group analysis （Control Group Type）

Figure 4: The forest plot of HAMD-17 group analysis （Acupuncture sites）

1

Study or Subgroup	Experimental Mean	Experimental SD	Experimental Total	Control Mean	Control SD	Control Total	Weight	Std. Mean Difference IV, Fixed, 95% CI
Deng 2008	8.1	3.53	29	10.31	6.33	29	7.1%	-0.43 [-0.95, 0.10]
Li 2015	9.33	3	30	8.75	3.27	30	7.5%	0.18 [-0.32, 0.69]
Li 2018	9.65	4.39	116	11.06	4.1	105	27.3%	-0.33 [-0.60, -0.06]
Liu 2022	8.63	3.15	32	10.66	3.87	32	7.7%	-0.57 [-1.07, -0.07]
Qian 2007	10.58	4.18	33	11.24	4.15	30	7.9%	-0.16 [-0.65, 0.34]
Wang 2010	8.6	3.9	30	10.6	6.9	30	7.4%	-0.35 [-0.86, 0.16]
Zhao 2023	10.14	3.85	35	10.49	2.37	35	8.8%	-0.11 [-0.58, 0.36]
Zhou 2022	9.61	4.63	108	10.97	4.34	104	26.3%	-0.30 [-0.57, -0.03]
**Total (95% CI)**			**413**			**395**	**100.0%**	**-0.28 [-0.42, -0.14]**

Heterogeneity: Chi² = 5.76, df = 7 (P = 0.57); I² = 0%

Test for overall effect: Z = 3.92 (P < 0.0001)

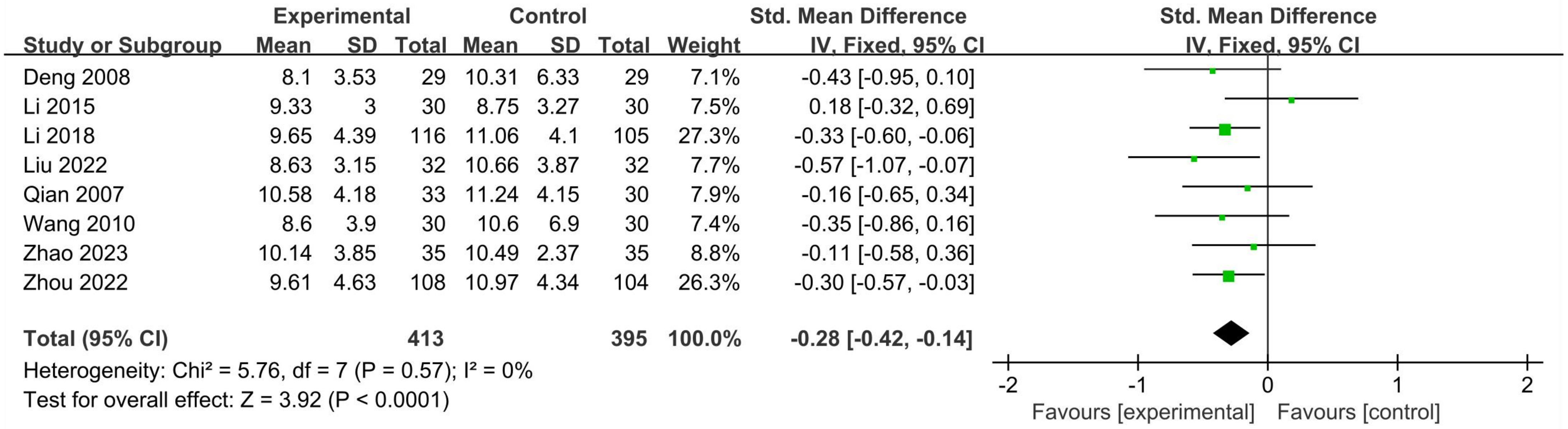

2

Study or Subgroup	Experimental Mean	Experimental SD	Experimental Total	Control Mean	Control SD	Control Total	Weight	Std. Mean Difference IV, Fixed, 95% CI
**1.2.1 EA**								
Li 2015	9.33	3	30	8.75	3.27	30	7.5%	0.18 [-0.32, 0.69]
Li 2018	9.65	4.39	116	11.06	4.1	105	27.3%	-0.33 [-0.60, -0.06]
Zhou 2022	9.61	4.63	108	10.97	4.34	104	26.3%	-0.30 [-0.57, -0.03]
**Subtotal (95% CI)**			**254**			**239**	**61.1%**	**-0.26 [-0.43, -0.08]**
Heterogeneity: Chi² = 3.28, df = 2 (P = 0.19); I² = 39%								
Test for overall effect: Z = 2.81 (P = 0.005)								
**1.2.2 MA**								
Deng 2008	8.1	3.53	29	10.31	6.33	29	7.1%	-0.43 [-0.95, 0.10]
Liu 2022	8.63	3.15	32	10.66	3.87	32	7.7%	-0.57 [-1.07, -0.07]
Qian 2007	10.58	4.18	33	11.24	4.15	30	7.9%	-0.16 [-0.65, 0.34]
Wang 2010	8.6	3.9	30	10.6	6.9	30	7.4%	-0.35 [-0.86, 0.16]
Zhao 2023	10.14	3.85	35	10.49	2.37	35	8.8%	-0.11 [-0.58, 0.36]
**Subtotal (95% CI)**			**159**			**156**	**38.9%**	**-0.31 [-0.54, -0.09]**
Heterogeneity: Chi² = 2.32, df = 4 (P = 0.68); I² = 0%								
Test for overall effect: Z = 2.76 (P = 0.006)								
**Total (95% CI)**			**413**			**395**	**100.0%**	**-0.28 [-0.42, -0.14]**
Heterogeneity: Chi² = 5.76, df = 7 (P = 0.57); I² = 0%								
Test for overall effect: Z = 3.92 (P < 0.0001)								
Test for subgroup differences: Chi² = 0.16, df = 1 (P = 0.69), I² = 0%								

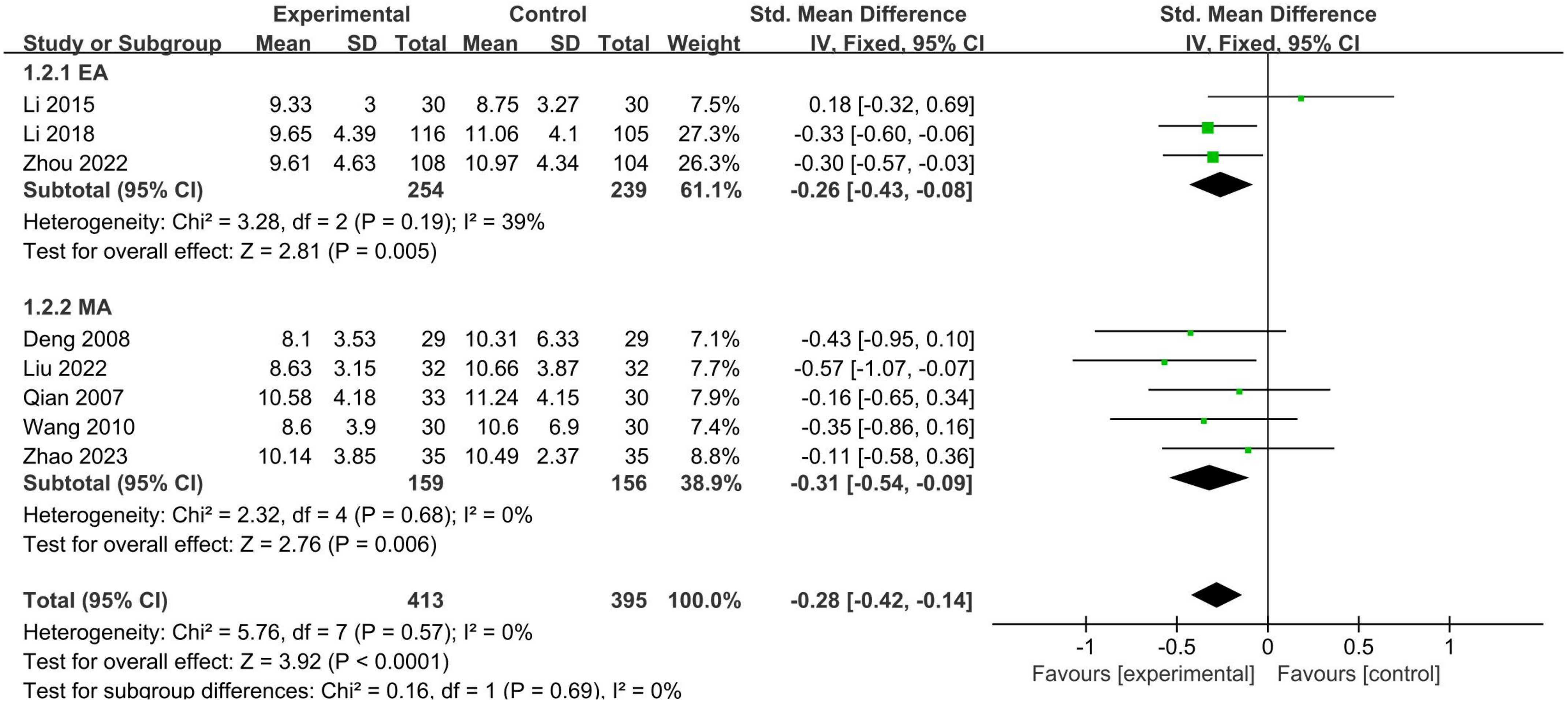

3

Study or Subgroup	Experimental Mean	Experimental SD	Experimental Total	Control Mean	Control SD	Control Total	Weight	Std. Mean Difference IV, Fixed, 95% CI
**1.5.1 CHM**								
Liu 2022	8.63	3.15	32	10.66	3.87	32	7.7%	-0.57 [-1.07, -0.07]
**Subtotal (95% CI)**			**32**			**32**	**7.7%**	**-0.57 [-1.07, -0.07]**
Heterogeneity: Not applicable								
Test for overall effect: Z = 2.23 (P = 0.03)								
**1.5.2 other medicine**								
Deng 2008	8.1	3.53	29	10.31	6.33	29	7.1%	-0.43 [-0.95, 0.10]
Li 2015	9.33	3	30	8.75	3.27	30	7.5%	0.18 [-0.32, 0.69]
Li 2018	9.65	4.39	116	11.06	4.1	105	27.3%	-0.33 [-0.60, -0.06]
Qian 2007	10.58	4.18	33	11.24	4.15	30	7.9%	-0.16 [-0.65, 0.34]
Wang 2010	8.6	3.9	30	10.6	6.9	30	7.4%	-0.35 [-0.86, 0.16]
Zhou 2022	9.61	4.63	108	10.97	4.34	104	26.3%	-0.30 [-0.57, -0.03]
**Subtotal (95% CI)**			**346**			**328**	**83.5%**	**-0.27 [-0.42, -0.12]**
Heterogeneity: Chi² = 3.95, df = 5 (P = 0.56); I² = 0%								
Test for overall effect: Z = 3.47 (P = 0.0005)								
**1.5.3 sham acupuncture**								
Zhao 2023	10.14	3.85	35	10.49	2.37	35	8.8%	-0.11 [-0.58, 0.36]
**Subtotal (95% CI)**			**35**			**35**	**8.8%**	**-0.11 [-0.58, 0.36]**
Heterogeneity: Not applicable								
Test for overall effect: Z = 0.45 (P = 0.65)								
**Total (95% CI)**			**413**			**395**	**100.0%**	**-0.28 [-0.42, -0.14]**
Heterogeneity: Chi² = 5.76, df = 7 (P = 0.57); I² = 0%								
Test for overall effect: Z = 3.92 (P < 0.0001)								
Test for subgroup differences: Chi² = 1.81, df = 2 (P = 0.40), I² = 0%								

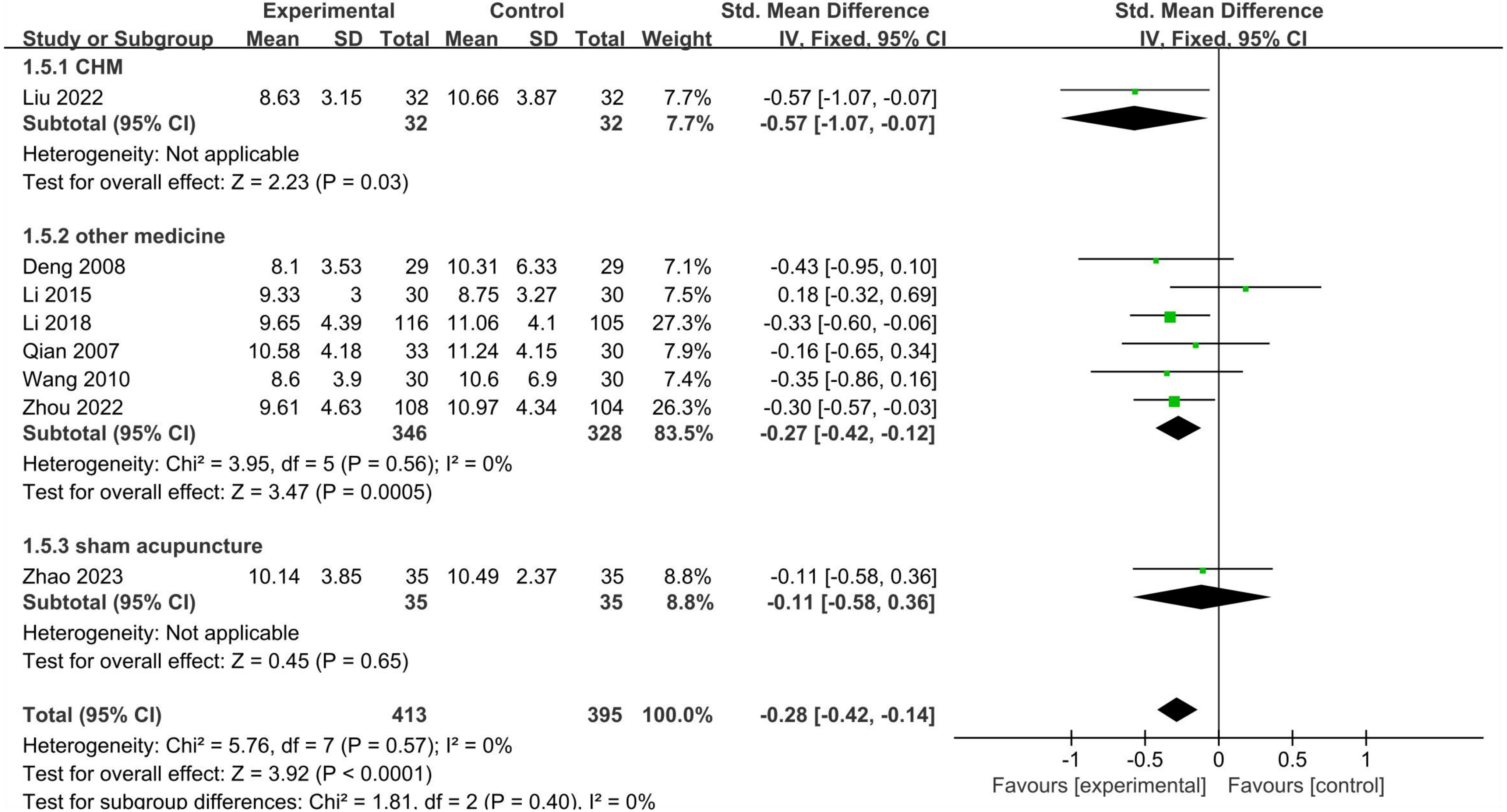

4

Study or Subgroup	Experimental Mean	Experimental SD	Experimental Total	Control Mean	Control SD	Control Total	Weight	Std. Mean Difference IV, Fixed, 95% CI
**1.3.1 Simple abdominal acupuncture**								
Deng 2008	8.1	3.53	29	10.31	6.33	29	7.1%	-0.43 [-0.95, 0.10]
Wang 2010	8.6	3.9	30	10.6	6.9	30	7.4%	-0.35 [-0.86, 0.16]
**Subtotal (95% CI)**			**59**			**59**	**14.5%**	**-0.39 [-0.75, -0.02]**
Heterogeneity: Chi² = 0.04, df = 1 (P = 0.84); I² = 0%								
Test for overall effect: Z = 2.09 (P = 0.04)								
**1.3.2 Non - simple abdominal acupuncture**								
Li 2015	9.33	3	30	8.75	3.27	30	7.5%	0.18 [-0.32, 0.69]
Li 2018	9.65	4.39	116	11.06	4.1	105	27.3%	-0.33 [-0.60, -0.06]
Liu 2022	8.63	3.15	32	10.66	3.87	32	7.7%	-0.57 [-1.07, -0.07]
Qian 2007	10.58	4.18	33	11.24	4.15	30	7.9%	-0.16 [-0.65, 0.34]
Zhao 2023	10.14	3.85	35	10.49	2.37	35	8.8%	-0.11 [-0.58, 0.36]
Zhou 2022	9.61	4.63	108	10.97	4.34	104	26.3%	-0.30 [-0.57, -0.03]
**Subtotal (95% CI)**			**354**			**336**	**85.5%**	**-0.26 [-0.41, -0.11]**
Heterogeneity: Chi² = 5.31, df = 5 (P = 0.38); I² = 6%								
Test for overall effect: Z = 3.38 (P = 0.0007)								
**Total (95% CI)**			**413**			**395**	**100.0%**	**-0.28 [-0.42, -0.14]**
Heterogeneity: Chi² = 5.76, df = 7 (P = 0.57); I² = 0%								
Test for overall effect: Z = 3.92 (P < 0.0001)								
Test for subgroup differences: Chi² = 0.41, df = 1 (P = 0.52), I² = 0%								

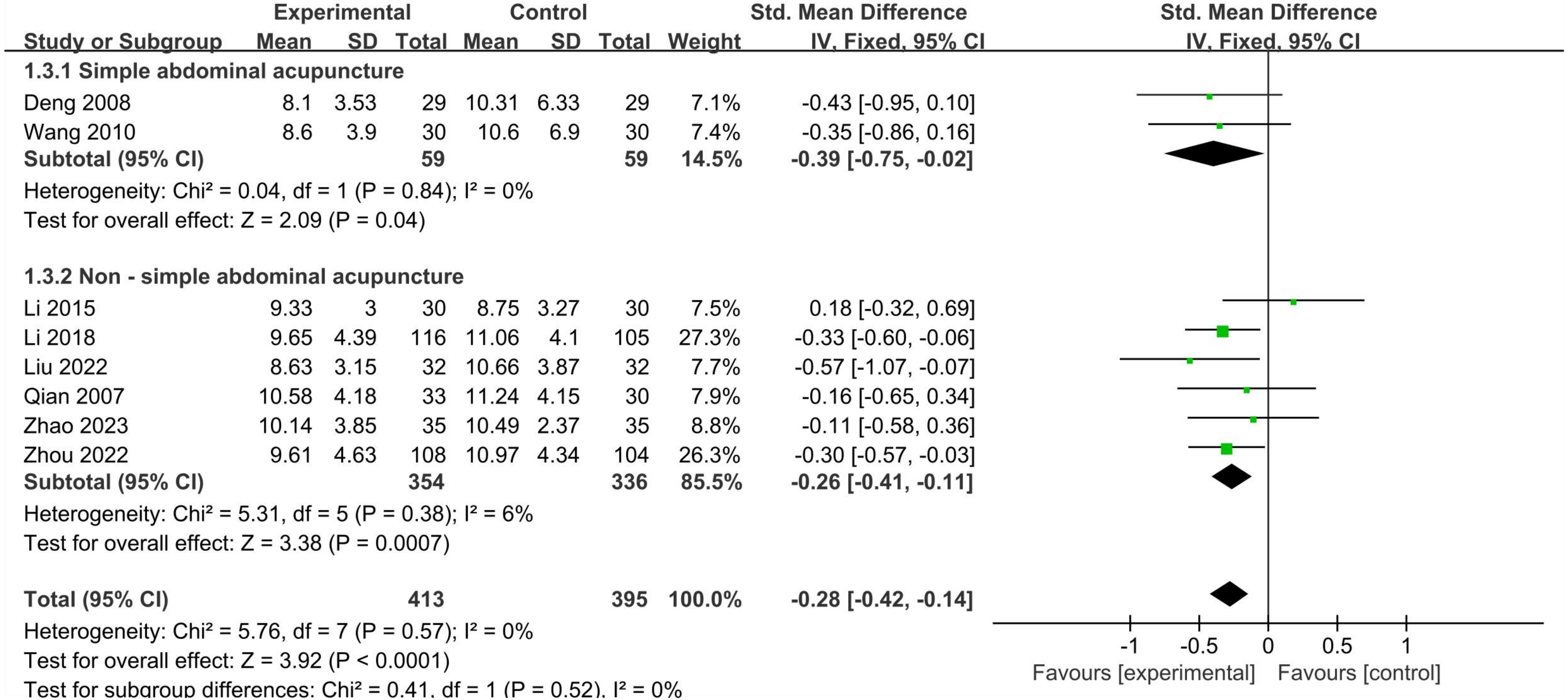

Supplement: Supplementary 1 — Clinical effectiveness rate. [file SupplementaryFile1.zip › Supplementary material 2.PDF]
